# Characterization of the Secretome, Transcriptome, and Proteome of Human β Cell Line EndoC-βH1

**DOI:** 10.1016/j.mcpro.2022.100229

**Published:** 2022-04-02

**Authors:** Maria Ryaboshapkina, Kevin Saitoski, Ghaith M. Hamza, Andrew F. Jarnuczak, Séverine Pechberty, Claire Berthault, Kaushik Sengupta, Christina Rye Underwood, Shalini Andersson, Raphael Scharfmann

**Affiliations:** 1 Translational Science and Experimental Medicine, Research and Early Development, Cardiovascular, Renal and Metabolism (CVRM), BioPharmaceuticals R&D, AstraZeneca, Gothenburg, Sweden; 2 Université de Paris, Institut Cochin, INSERM U1016, CNRS UMR 8104, Paris, France; 3 Discovery Sciences, AstraZeneca, Boston, Massachusetts, USA; 4 Molecular, Cellular and Biomedical Sciences, University of New Hampshire, Durham, New Hampshire, USA; 5 Quantitative Biology, Discovery Sciences, BioPharmaceuticals R&D, AstraZeneca, Cambridge, United Kingdom; 6 Alliance Management, Business Development, Licensing and Strategy, Biopharmaceuticals R&D, Astra Zeneca, Gothenburg, Sweden; 7 Bioscience Metabolism, Research and Early Development, Cardiovascular, Renal and Metabolism (CVRM), BioPharmaceuticals R&D, AstraZeneca, Cambridge, United Kingdom; 8 Oligonucleotide Discovery, Discovery Sciences, BioPharmaceuticals R&D, AstraZeneca, Gothenburg, Sweden

**Keywords:** pancreatic beta cell, EndoC-bH1, *in vitro* model validation, secretome, ACN, acetonitrile, BSA, bovine serum albumin, DIA, Data-Independent Acquisition, EV, Extracellular Vesicle, FA, formic acid, FACS, Fluorescence-activated cell sorting, FDR, False Discovery Rate, GLP1R, glucagon-like peptide 1 receptor, GPCR, G-protein–coupled receptor, TMT, Tandem Mass Tag, TPM, transcripts per million

## Abstract

Early diabetes research is hampered by limited availability, variable quality, and instability of human pancreatic islets in culture. Little is known about the human β cell secretome, and recent studies question translatability of rodent β cell secretory profiles. Here, we verify representativeness of EndoC-βH1, one of the most widely used human β cell lines, as a translational human β cell model based on omics and characterize the EndoC-βH1 secretome. We profiled EndoC-βH1 cells using RNA-seq, data-independent acquisition, and tandem mass tag proteomics of cell lysate. Omics profiles of EndoC-βH1 cells were compared to human β cells and insulinomas. Secretome composition was assessed by data-independent acquisition proteomics. Agreement between EndoC-βH1 cells and primary adult human β cells was ∼90% for global omics profiles as well as for β cell markers, transcription factors, and enzymes. Discrepancies in expression were due to elevated proliferation rate of EndoC-βH1 cells compared to adult β cells. Consistently, similarity was slightly higher with benign nonmetastatic insulinomas. EndoC-βH1 secreted 783 proteins in untreated baseline state and 3135 proteins when stressed with nontargeting control siRNA, including known β cell hormones INS, IAPP, and IGF2. Further, EndoC-βH1 secreted proteins known to generate bioactive peptides such as granins and enzymes required for production of bioactive peptides. EndoC-βH1 secretome contained an unexpectedly high proportion of predicted extracellular vesicle proteins. We believe that secretion of extracellular vesicles and bioactive peptides warrant further investigation with specialized proteomics workflows in future studies.

## Highlights

• We validate EndoC-βH1 as a translational human β cell model using omics.
• We present the first unbiased proteomics composition of human β cell line secretome.
• The secretome of human β cells is more extensive than previously thought.
• Untreated cells secreted 783 proteins, and stressed cells secreted 3135 proteins.
• Secreted extracellular vesicles and bioactive peptides are subject of future work.

## Graphical Abstract

**Figure:**
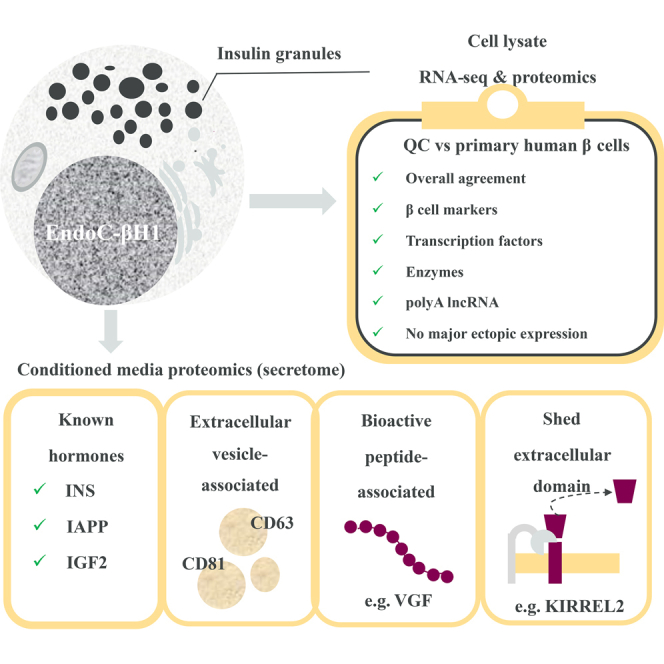

## In Brief

Our knowledge of β cell secretome originates predominantly from rodent studies. Recently, the translatability of rodent β cell secretory profiles has been questioned, emphasizing the need to characterize human β cell secretome in an unbiased manner. Here, we verify that EndoC-βH1 cell line is a representative human β cell model based on RNA-seq and cell lysate proteomics and present the first global secretome proteomics of EndoC-βH1 cells.

Close to half a billion people are living with diabetes worldwide, and this number is expected to increase by 25% in 2030 (1). Despite diabetes being a global health burden, the mechanisms underlying normal β cell function and failure are not yet fully elucidated. This is partly due to limited availability of high-quality human pancreatic islets for research purposes combined with variable quality and variation in relative cell type composition of islet preparations (2, 3). Moreover, primary human islets rapidly lose functional expression pattern in culture (4). To overcome these limitations, pancreatic islets from rodents have been used to study β cell biology for decades leading to significant advances in our understanding of the disrupted pathways causing impaired β cell function. However, the cellular architecture and secretory profile of rodent islets is substantially different from that of human islets (5, 6), manifesting the need for human β cell lines, which mirror the expression and secretory pattern of primary human β cells.

Several human β cell lines are currently available—1.1B4, 1.4E7 and 1.1E7 (7), T6PNE (8), EndoC-βH1 (9), EndoC-βH2 (10), EndoC-βH3 (11) and ECN90 (12). The corresponding full-text searches for these cells lines in Google Scholar retrieved 774 (22.5%), 993 (28.8%), 947 (27.5%), 13 (0.4%), 598 (17.4%), 67 (1.9%), 41(1.2%), and 11(0.3%) entries as of March 2021, respectively. Thus, EndoC-βH1 cells remain one of the most commonly used human β cell lines despite availability of next-generation cell lines EndoC-βH2 (10) and EndoC-βH3 (11) with inducible growth arrest. Taken together, these three generations of EndoC-βH cells are used by over 150 research labs world-wide (https://www.humanbetacelllines.com/) (13).

Validity of EndoC-βH1 cells as a human β cell model has been previously established based on functional experiments such as glucose-stimulated insulin secretion (14, 15, 16) and marker expression profiling by qPCR (9, 16). Recently, EndoC-βH1 open chromatin, transcriptomics, and miRNA landscapes were reported to be most similar to adult human β cells or islets compared to α cells, adipocytes, and muscle and peripheral blood cells (17). Two proteomics studies investigated the response to interferons in EndoC-βH1 cells (18, 19). Yet, a thorough systematic comparison of EndoC-βH1 omics profiles to primary β cells and evaluation of potential ectopic expression patterns have not been performed. It remains unclear what are the molecular discrepancies of EndoC-βH1 with adult β cells that could further inform the scope/applicability of this cell line.

The aim of this study is to complement the prior functional studies (13, 14, 15, 16) and validate EndoC-βH1 as an *in vitro* human β cell model based on omics. We use control samples from a larger study investigating effects of PCSK9 knockdown (20) to evaluate the EndoC-βH1 cell transcriptome and proteome against corresponding omics profiles of primary β cells and insulinoma samples. Due to their proliferative nature, EndoC-βH1 cells are more similar to human primary insulinomas than to adult human β cells which have an extremely low proliferation rate. We show that EndoC-βH1 cells belong to the β cell lineage and recapitulate features of adult primary β cells well. We observe that EndoC-βH1 cells exhibit ∼90% similarity with reference adult human β cell transcriptome and proteome. Similarity exceeding 90% was noted for β cell markers, transcription factors, and enzymes. We also extensively studied negative control markers of other cell and tissue lineages and confirm lack of major expression abnormalities.

Further, we address a major knowledge gap: β cells are a crucial endocrine cell type, yet little is known about the β cell secretome, especially in humans. Few studies reported secretome composition of rodent insulinoma cell lines (21, 22, 23), while the secretome of human immune cell–depleted islet tissue has been profiled using the SOMAscan 1300 proteomics assay only in 2021 (24). The SOMAscan study suggests that the human and murine secretomes may be quite distinct (24), making it unclear to what extent findings of secretome studies in murine β cells can be extrapolated to human. In this study, we present the first characterization of human β cell line EndoC-βH1 secretome by data-independent acquisition (DIA) proteomics. These novel data indicate that the secretome of β cells may be more extensive than previously thought and highlights surprising abundance of proteins predicted to be secreted *via* extracellular vesicles (EVs). We subsequently refer to these proteins as EV-associated proteins.

## Experimental Procedures

### Experimental Design and Statistical Rationale

The experiment series was designed to investigate the effects of PCSK9 knockdown on β cell transcriptome, proteome, and secretome. We used nongrowth-arrested EndoC-βH1 cell line because it is one of the most commonly used human β cell lines, and it enables generation of a large number of cells needed for secretome proteomics (in total, >100 million cells for our samples). In total, one RNA-seq and five proteomics experiments were conducted with at least three biological replicates per condition (supplemental Table S1). Nontargeting control siRNA and untransfected samples were used to characterize EndoC-βH1 and are presented in this manuscript. Effects of PCSK9 knockdown are presented in a follow-up study (20).

For all experiments, the PCSK9 knockdown was confirmed at the mRNA level by RT-qPCR and at the protein level by Western Blot in both cell extracts and conditioned media prior to omics data acquisition. However, PCSK9 knockdown in tandem mass tag (TMT) experiment PXD027898 could not be detected due to small effect size *versus* plex-effect. We used PXD027898 only to verify lack of major impact of control siRNA treatment on proteome composition. All other experiments were used to support both manuscripts.

#### Sample Size Calculation

GSE182016, PXD027921, PXD027898, and PXD027920 were unpowered pilot experiments with three biological replicates per condition. Sample size for PXD027911 and PXD027913 was calculated based on PCSK9 knockdown in the pilot cell lysate TMT experiment PXD027921 as 12 nontargeting control siRNA and 12 siPCSK9 samples (power 80%, alpha 0.05, N proteins expected to be detected 9,000, alpha corrected for multiple testing 5.6e-6, Cohen d for PCSK9 knockdown -2.93, distribution of normalized protein intensities assumed to be approximately normal, two-tailed *t* test).

### EndoC-βH1 Cell Culture

EndoC-βH1 cells were cultivated in low glucose Dulbecco's modified Eagle's medium (5,6 mmol/l; Sigma-Aldrich) supplemented with 2% bovine serum albumin (BSA) fraction V (Roche Diagnostics), 50 μmol/L 2-β-mercaptoethanol (Sigma-Aldrich), 10 mmol/L nicotinamide (Calbiochem), 5.5 μg/ml transferrin (Sigma-Aldrich), 6.7 ng/ml sodium selenite (Sigma-Aldrich), 100 units/ml penicillin, and 100 μg/ml streptomycin (ThermoFisher Scientific). The cells were seeded on plates coated with 1.2% Matrigel containing-3 μg/ml fibronectin (both from Sigma-Aldrich) and cultured at 37 °C and 5% CO2 as previously described (9).

For siRNA transfection, EndoC-βH1 cells were passaged and seeded at 10^5^ cells/cm^2^. Twenty-four hours later, cells were transfected in OptiMEM using Lipofectamine RNAiMAX (Life Technologies) with siRNA SMARTpools (Horizon Discovery LTD): nontargeting control (siCTRL, D-001810–01–20) or siRNA PCSK9 (M-005989–01–0005) at the final concentration of 80 nM as described (25). Medium was replaced 2.5 h later with fresh EndoC-βH1 culture medium.

For proteome experiments, EndoC-βH1 cells were preincubated for 2 h in BSA/phenol red-free culture medium before transfection. After transfection, the cells were grown in fresh culture medium without BSA and without phenol red for 3 days. Cell pellets were collected by trypsinization and washed three times in PBS. Conditioned media were collected, treated with protease and phosphatase inhibitors (Roche Diagnostics, 05892791001; 04906837001), and the debris was eliminated by centrifugation at 1500 rpm at 4 °C for 5 min and filtered through 0.22 μm-diameter filters. All the samples were stored at −80 °C.

### Fluorescence-Activated Cell Sorting

EndoC-βH1 cells were trypsinized and washed three times in PBS. Cell death was assessed by incubating the cells with annexin-V antibody (APC, #*350520,* Biolegend) for 15 min at room temperature in the dark in annexin-V buffer (#*422201,* Biolegend). Propidium iodide was added before fluorescence-activated cell sorting (FACS) analysis. For proliferation studies, cells were fixed and permeabilized with a transcription factor staining buffer set (#*00–5523–00,* Thermo Fisher Scientific) according to the manufacturer’s instructions. Cells were then incubated with Ki67 antibody (PerCP/Cy5.5, #*B238642,* Biolegend) for 30 min at room temperature in the dark in permeabilization buffer and then with DAPI (BD Biosciences) for 10 min at room temperature in the dark. FACS analysis was carried out using a FACS Aria III (BD Biosciences). Data were analyzed using FlowJo 10.7 software (RRID:SCR_008520).

### RT-qPCR

A RNeasy Micro Kit (Qiagen) was used to extract total RNA from EndoC-βH1 cells (25). Genomic DNA was removed by DNAse treatment following the RNeasy Micro Kit protocol. RNAs were reverse transcribed by using the Maxima First Strand cDNA kit (Thermo Fisher Scientific). RT-qPCR was performed using Power SYBR Green mix (Applied Biosystems) with a QuantStudio 3 analyzer (Thermo Fisher Scientific). Custom primers (supplemental Table S2) were designed with Primer-Blast online, and their efficiency and specificity were determined for each pair by RT-qPCR on a serial dilution of cDNA samples. Relative quantification (2ˆ-dCt method) was used to calculate the expression levels of each target gene, normalized to CYCLOPHILIN-A transcripts.

### Insulin Content

EndoC-βH1 cells were transfected with control siRNA and cultured with 2% BSA or without BSA. Three days later, cells were washed three times with PBS and collected following trypsin treatment. Intracellular insulin content was measured by ELISA (Mercodia AB) as previously described (9).

### RNA Sequencing

RNeasy Micro Kit (Qiagen) was used to extract total RNA from EndoC-βH1 cells. Genomic DNA was removed by DNAse treatment following the RNeasy Micro Kit protocol. RNA quality (RNA integrity number) was determined by electrophoresis using the Agilent 2100 Bioanalyzer (Agilent Technologies) as per the manufacturer’s instructions. To construct the libraries, 600 ng of high-quality total RNA sample (RIN >8.5) was processed using TruSeq Stranded mRNA kit (Illumina) according to manufacturer’s instructions. Briefly, after purification of poly-A containing mRNA molecules, mRNA molecules were fragmented and reverse-transcribed using random primers. Replacement of dTTP by dUTP during the second strand synthesis enabled strand specificity. The addition of a single A base to the cDNA was followed by ligation of Illumina adapters. Then, the libraries were quantified with Qubit fluorometer DNA HS kit (Invitrogen). The library profiles were assessed using the DNA High Sensitivity LabChip kit on an Agilent Bioanalyzer. Libraries were sequenced on an Illumina Nextseq 500 instrument using 75 base-length reads V2.5 chemistry in a paired-end mode. After sequencing, a primary analysis based on AOZAN software (Ecole Normale Supérieure) was applied to demultiplex and control the quality of the raw data (based on FastQC modules/version 0.11.9). Raw reads were aligned to human genome GRCh38 release 96 using STAR, version 2.7.1a (26). Transcript quantification was performed with RSEM (27). Quality control metrics were generated with Picard [“Picard Toolkit.” 2019. Broad Institute, GitHub Repository. http://broadinstitute.github.io/picard/; Broad Institute] and STAR (26) and examined visually in addition to clustering and principal component analysis. All samples passed quality control. TPM (transcript per million) normalization was applied for descriptive analysis in this study.

### Cell Proteome Sample Preparation

Cell pellets were thawed on ice and subjected to sample preparation with the PreOmics iST NHS or iST kit (PreOmics) according to manufacturer's protocols. Briefly, iST or iST-NHS lysis buffer was added directly to the cell pellet and incubated at 95 °C for 10 min for cell lysis, reduction, and alkylation of proteins. Cell lysate was normalized using BCA assay (Thermo Fisher Scientific), and 50 μg from each condition was subjected to enzymatic cleavage for 3 h by adding equal amounts of endoproteinase Lys-C and trypsin (ThermoFisher Scientific, # A40009) in a 1:50 (wt/wt) enzyme:protein ratio. For experiments utilizing TMT, TMT reagents were reconstituted in anhydrous acetonitrile (ACN) and added to peptides at a 4:1 reagent:peptide. De-salting and purification were performed according to the PreOmics iST or PreOmics iST-NHS protocol on a styrene divinylbenzene reversed-phase sulfonate sorbent. IST Purified peptides were vacuum-centrifuged to dryness and reconstituted in double-distilled water with 2 vol% ACN and 0.1 vol% formic acid (FA) for single-run LC-MS analysis. IST-NHS TMT-labeled peptides were reconstituted in pH 10.0, 20 mM ammonium hydroxide, and combined evenly to create a single sample.

### High pH Reversed-Phase Fractionation

TMT-labeled peptides were fractionated into 96 fractions across a 96-well plate, using high pH reverse phase chromatography on an Agilent 1100 system and Phenomenex Gemini 5 μm C18 250 mm × 2 mm column. The linear gradient profile consisted of 2% to 90% mobile phase B in 70 min at flow rate of 0.20 ml/min. Mobile phase A consisted of pH 10.0, 20 mM ammonium hydroxide in water, and mobile phase B consisted of 20 mM ammonium hydroxide in ACN. The 96 fractions were combined within each column orthogonally into a total of 12 concatenated analytical samples. Samples were vacuum-centrifuged to dryness and were then reconstituted with double-distilled water with 2 vol% ACN and 0.1 vol% FA for LC-MS analysis.

### Secretome Sample Preparation

Cell conditioned media were thawed on ice and were concentrated down to ∼100 μl using Amicon Ultra-15 3k molecular weight cut off (MWCO) centrifugal filter units (Millipore Sigma). Buffer exchange into PreOmics iST lysis buffer was performed, followed by incubation at 95 °C for 10 min for reduction and alkylation of proteins. Protein amounts were normalized using BCA assay (Thermo Fisher Scientific), and equal amounts from each condition were subjected to enzymatic cleavage for 3 h by adding equal amounts of endoproteinase Lys-C and trypsin (ThermoFisher Scientific, # A40009) in a 1:50 (wt/wt) enzyme:protein ratio. De-salting and purification were performed according to the PreOmics iST protocol on a styrene divinylbenzene reversed-phase sulfonate sorbent. Purified peptides were vacuum-centrifuged to dryness and reconstituted in double-distilled water with 2 vol% ACN and 0.1 vol% FA for single-run LC-MS analysis.

### LC-MS/MS Measurement

#### Orbitrap Exploris 480 Mass Spectrometer (PXD027911 and PXD027920)

Peptides were loaded onto a 25 cm IonOpticks Aurora Series emitter column (25 cm × 75u ID, 1.6um C18; IonOpticks) performed by a Dionex Ultimate 3000 coupled online to an Exploris 480 Mass Spectrometer equipped with a Nanospray Flex Ion Source, integrated with a column oven (PRSO-V1, Sonation) maintained at 50 °C. Peptides were separated using a nonlinear gradient. Mobile phase A was 0.1 vol% FA and 3 vol% ACN in water, while Mobile phase B was 90 vol% can and 0.1 vol % FA. The gradient was operated at 400 nl/min flowing 3 vol% B for 25 min, 5 to 17 vol% B over 72 min, 17 to 24 vol % B over 18 min, 24 to 30 vol % B over 10 min, 30 to 85 vol % B over 3 min, hold at 85 vol% B for 7 min, 85 to 3 vol% B over 0.1 min, and hold at 3 vol% B for 15 min. Orbitrap Exploris 480 was operated in BoxCar DIA-positive mode where spray voltage was set to 1600V, funnel RF at 40%, and heated capillary temperature at 275 °C. Method timeline experiment consisted of one MS1 scan, one tSIM scan, and one tMS2 scan. MS1 scan was operated at 120k resolution, 400 to 1200 m/z scan range, 40% RF lens, 300% AGC target, and 54 ms IT. MS1 tSIM was operated with multiplexed ions enabled (12 ions), at 120k resolution, 300% AGC target, 20 ms IT with a set loop control of 2N (number of spectra). BoxCar windows spanned 400 to 1200 m/z space. A total of 48 MS2 DIA variable windows were operated at 15k resolution with normalized collision energy of 28%, 1000% AGC target with 22 ms IT spanning 400 to 1200 m/z range taken into account heavily dense peptide regions.

#### Orbitrap Fusion Lumos Mass Spectrometer (PXD027913)

Peptides were loaded onto a ReproSil-Pur 120 C18AQ 1.9um in-house packed to a 5um tip 75u ID × 360u × 50 cm column using a Thermo EASY-nLC1200 connected through a Nanospray Flex Ion Source, integrated with a Column Oven (PRSO-V1, Sonation) maintained at 50 °C. Mobile phase A was 0.1 vol% FA and 3 vol% ACN in water, while Mobile phase B was 90 vol% can and 0.1 vol% FA. The gradient which had a flow of 300 nl/min consisted of a nonlinear ramp of 3 to 24 vol% B in 85 min, 24 to 30 vol% B for 15 min, 30 to 95 vol% B in 5 min, 95 vol% B hold for 5 min, and reequilibration to 3 vol% B for 10 min. The LC was connected to an Orbitrap Fusion Lumos Mass Spectrometer operated in DIA-positive mode. Briefly, spray voltage was set to 2500V, ion transfer tube set to 300 °C, MS1 resolution at 120k, MS2 resolution at 30k, and scan range of 350 to 1650 m/z for MS1 and 350 to 1650 m/z for MS2. RF lens was set to 30%, MS1 IT to 20 ms, and MS2 IT to 60 ms. MS1 AGC target set to 3e6, MS2 AGC target set to 1.5e6 with 28% higher-energy C-trap dissociation collision energy. DIA variable windows covered 400 to 1650 m/z space taken into account heavily dense peptide regions.

#### Orbitrap Fusion Lumos Mass Spectrometer (PXD027898 and PXD027921)

Peptides were loaded onto a ReproSil-Pur 120 C18AQ 1.9um in-house packed to a 5um tip 75u ID × 360u × 50 cm bed volume column using a Thermo EASY-nLC1200 connected through a Nanospray Flex Ion Source, integrated with a Column Oven (PRSO-V1, Sonation) maintained at 50 °C. Mobile phase A was 0.1 vol% FA, 3 vol% ACN in water, while Mobile phase B was 90 vol% ACN and 0.1 vol% FA. The gradient which had a flow of 250 nl/min consisted of nonlinear ramp of 3 to 5 vol% B in 5 min, 5 to 20 vol% B for 80 min, 20 to 32 vol% B in 30 min, 32 to 95 vol% B in 1 min, and hold at 95 vol% B for 14 min. The LC was connected to an Orbitrap Fusion Lumos Mass Spectrometer operated in data-dependent acquisition–positive mode. Briefly, spray voltage was set to 2200V, ion transfer tube set to 275 °C, MS1 resolution at 60k, MS2 resolution at 15k, scan range was set to 375 to 2000 m/z for MS1, first mass of 100 m/z for MS2, MS1 IT to 50 ms, MS2 IT to 22 ms, MS1 AGC target set to 1e6, and MS2 AGC target set to 1e6 with 38% higher-energy C-trap dissociation collision energy. Quadrupole isolation window was set to 1 m/z with 30 s exclusion duration. Monoisotopic precursor selection filter was set to peptide, filter intensity threshold was activated with maximum intensity set to 1E20, minimum intensity set to 4E4, and intensity filter type set to intensity threshold.

### Mass Spectrometry Analysis

DIA experiments were analyzed using Spectronaut V15.5 (Biognosys AG) direct DIA analysis using a combined human, mouse (UniProt downloaded 22.02.2021) and MaxQuant contaminants (28) database utilizing the Pulsar search engine. Analysis settings were maintained to factory settings where identification was set to 1% false discovery rate (FDR) for precursor and protein level. FDR was calculated with Spectronaut algorithm based on fraction of negative control/synthetic decoy peptides in the samples (29). Quantification was conducted on MS2 level for specific digest type of Trypsin/P. Static modifications of carbamidomethyl (+57.021 Da) and dynamic modification N-terminal acetylation (+42.011 Da) were used.

TMT experiments were analyzed using Thermo Proteome Discoverer 2.4. Briefly, the raw files were searched through Sequest HT using a combined human, mouse (UniProt downloaded 22.02.2021) and MaxQuant contaminants (28) database with the following parameters: trypsin (full), max missed cleavage of 2, minimum peptide length of 6, max peptide length of 144, precursor mass tolerance of 10 ppm, fragment mass tolerance of 0.02 Da, dynamic modification: oxidation (+15.995 Da) of methionine, deamidated asparagine and glutamine (+0.984 Da), N-terminal acetylation (+42.011 Da), N-terminal Met-loss (−131.040 Da), N-terminal Met-loss + Acetyl (−89.030), static modification: TMT (+229.163 Da) on peptide N-terminus and lysine, PreOmics cystine modification (+113.084 Da). FDR was estimated based on target/decoy principle (30). FDR on peptide and protein level was 1%. TMT reporter ion quantification was carried out using 50% co-isolation threshold and average reporter S/N threshold of 10.

### Downstream Analysis

TMT proteomics data were normalized using scaling factor normalization and log2 transformed. DIA proteomics data were normalized using quantile normalization as implemented in limma (31) and log2-transformed. DIA proteomics data contained small number of missing values: 0.8 (0.5–1.7)% missing protein intensity values per sample in cell lysate PXD027911 and 1.6 (1.1–2.2)% in secretome PXD027913 experiments. Missing values were imputed using sequential imputation method (32, 33). Approximately normal distribution of normalized protein intensities were confirmed by visual examination of quantile–quantile plots. Ambiguously annotated proteins (*e.g*., tryptic peptides aligning to several immunoglobulin kappa chain proteins) were excluded prior to statistical analysis. Differential protein expression analysis of siRNA control *versus* untreated cells was performed with mixed-effect linear model with condition as fixed effect term and TMT plex as random intercept. Functional classification of the proteins (34) was evaluated using AmiGO2 (35) and PANTHER (36). Proteins may have more than one functional annotation (*e.g.,* be involved in several pathways); therefore, the sum of percentages of proteins with different annotations may exceed 100%. Figures were made in R version 4.0.2 (37) with ggplot2 (38) and ggVennDiagram https://github.com/gaospecial/ggVennDiagram. EV-associated proteins were annotated based on ExoPred (39), Gene Ontology annotation (34) and known markers (40).

## Results

### Quality Control of the Biological Material

**Fig. 1:**
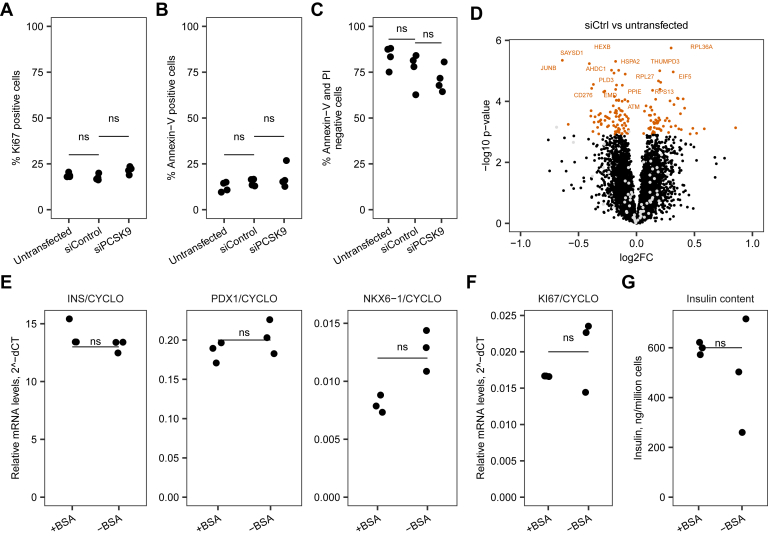
**Quality control of the cells.** Panels *A–D*, show lack of effect of transfection with nontargeting control siRNA on proliferation, apoptosis and cell identity. Ki67/Dapi staining for cell proliferation (*A*) and Annexin-V/PI staining for apoptosis (*B*), and live cells (*C*) were assessed by FACS in untransfected day 0 cells compared to 72 h post transfection with control siRNA or siPCSK9. *D*, cell lysate protein expression in EndoC-βH1 cells treated with control siRNA compared to untransfected day 0 cells in PXD027898. Each dot corresponds to a protein. Proteins in *orange* are differentially expressed. Proteins in *black* are not differentially expressed, among them β cell markers are highlighted in *gray*. Panels *E–G*, show lack of effect of BSA withdrawal on top of transfection with control nontargeting siRNA. EndoC-βH1 cells were transfected with control siRNA and then cultured in medium containing BSA (+BSA) or without BSA (-BSA). Analyses were performed 3 days later in biological triplicate per condition. *E*, expression of β cell markers by RT-qPCR. *F*, expression of proliferation marker KI67 by RT-qPCR. *G*, cells insulin content quantified by ELISA in cell pellets. BSA, bovine serum albumin; FACS, fluorescence-activated cell sorting.

One of the biggest challenges in secretome studies is determining whether the proteins are truly secreted by viable cells or are observed as a result of cell death or leakage. Therefore, we verified that treatment with nontargeting control siRNA did not drastically affect cell viability, cell type identity, or functional state. We confirmed that treatment with control siRNA did not affect cell proliferation (Fig. 1*A*) or viability (Fig 1, *B* and *C*, gating supplemental Fig. S1), which are important for secretome assessment as these factors could affect membrane permeability. We assessed the differences in protein expression between untransfected day 0 cells and control nontargeting siRNA–treated EndoC-βH1 cells in PXD027898. Treatment with control siRNA altered expression of 1.9% of proteins at Benjamini–Hochberg FDR <0.05. The cells treated with control siRNA displayed signs of altered lipid metabolism and stress (Fig. 1*D*). However, qualitative protein composition did not differ between untransfected day 0 and control siRNA–treated cells, and the differentially expressed proteins did not overlap with β cell markers. Thus, treatment with control siRNA did not change cell type identity.

We maintained EndoC-βH1 cells in the absence of serum, which is standard practice for this cell line (9), and did not glucose-starve the cells. 2% BSA was initially present in culture media but was removed 3 days prior to collection of conditioned media to avoid contaminants in the secretome experiments. We assessed key markers (Fig. 1, *E*–*G*) to test if withdrawal of BSA impacted the functional state of EndoC-βH1 cells. We observed no differences between cells cultured with *versus* without BSA.

### Computational Quality Control

We cultured the cells on mouse cell culture matrix Matrigel, so it was important to remove potential contaminants. We excluded mouse proteins and proteins from the contaminant library from further analysis. Some proteins could be either human or mouse due to shared sequence similarity. We estimated that 31% of unique tryptic peptide sequences could belong to either human or mouse using *in silico* digestion of our combined human, mouse, and contaminant database with Skyline (41). The fractions of ambiguous human/mouse protein species (*e.g.,* 26.1% proteins in PXD027913 and 15.0% PXD027911) were comparable or below the Skyline estimate. The ambiguous proteins had ∼20% overlap with known Matrigel composition, but the distributions of normalized intensities and missing values for ambiguous proteins were similar to human-unique proteins and not mouse-unique proteins or contaminants (supplemental Fig. S2). Therefore, we considered ambiguous proteins as human and retained them in subsequent analysis.

Next, we cross-referenced coordinates of peptides detected in the secretome experiments and mapped to membrane proteins with topology of proteins in the human surfaceome (42). If a secretome preparation is high-quality, peptides should map to extracellular regions that can be cleaved-off or shed. By contrast, contamination with peptides that map to transmembrane or intracellular regions indicates lysis or mechanical cell damage. The ratio of combined lengths of extracellular *versus* intracellular regions in the reference database was 2.4:1. This expected ratio was preserved in the cell lysate DIA experiment PXD027911 with 66.9% peptides mapped to extracellular regions and 29.8 % peptides mapped to intracellular regions. By contrast, 94.8% peptides mapped to extracellular regions in PXD027913 and 94.2% in PXD027920 secretome experiments. Cell lysate proteome in PXD027911 and secretome in PXD027913 were generated based on the same EndoC-βH1 cell cultures. Expression abundances of proteins with cytoplasmic subcellular location according to UniProt (43) did not correlate between cell lysate and the corresponding secretome samples—median IQR Spearman correlation 0.02 (−0.12–0.19)—indicating that leakage was unlikely. Proglucagon was not detected in the secretome experiments. Somatostatin was secreted by cells treated with control siRNA but not by untransfected EndoC-βH1 cells.

**Fig. 2:**
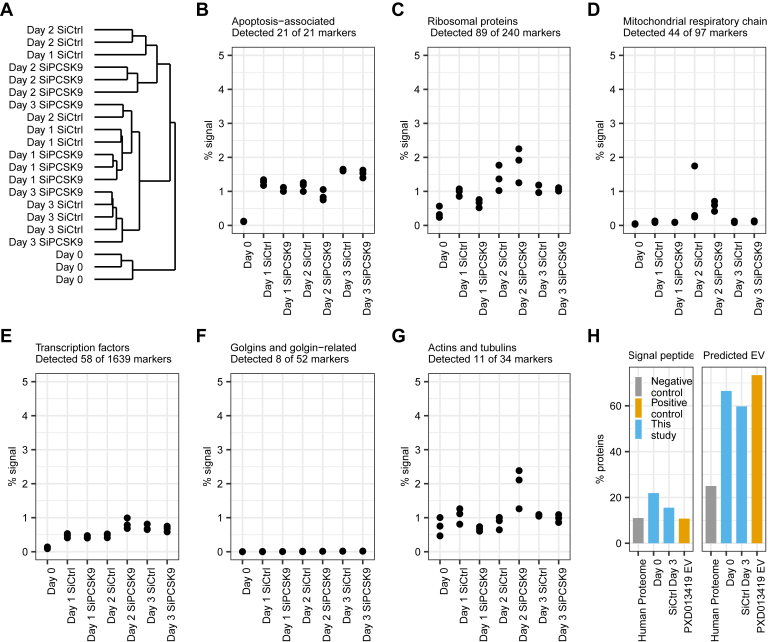
**Computational quality control of the secretome time course experiment****PXD027920****.***A*, hierarchical clustering. *B*–*G*, relative abundance of selected negative control marker sets. Relative abundance was calculated as sum of raw protein group intensities corresponding to the markers *versus* total in each sample. *H*, percentages of proteins containing signal peptide and proteins that can be secreted *via* extracellular vesicles (EV) predicted with ExoPred. Overall percentages of these protein categories in the human proteome were used as negative control and indicated based on UniProt and ExoCarta (*gray bars*).

Secretome time course experiment PXD027920 contained both untransfected day 0 samples and samples treated with siRNA. We followed the approach by Villarreal *et al.* (44) to assess if transfection had a negative impact on the quality of the secretome preparation. Untransfected day 0 samples clustered separately from siRNA-transfected samples (Fig. 2*A*), indicating a substantial difference of secretomes collected from cells stressed with a siRNA. The fraction of signal corresponding to secreted apoptosis markers (44) increased in siRNA-treated cells (Fig. 2*B*) but did not increase with time and, importantly, stayed under 2% (44) in agreement with FACS data showing no increase in apoptosis (Fig. 1, *B* and *C*). We selected several negative control marker sets corresponding to different intracellular structures based on HGNC annotation (45). The fraction of signal corresponding to these negative control markers was within 0 to 2% (Fig. 2, *C*–*G*). Thus, some of the negative control markers were detected albeit at low level. This led us to test if noncanonical secretion *via* EVs was a plausible explanation. We assessed proteins detected in ≥ 2 untransfected day 0 samples and ≥ 2 siControl samples at 72 h with ExoPred (39). Positive control was the proteome of EVs isolated by ultracentrifugation and with confirmed enrichment of CD63, CD81 and CD9 in the isolate by Western blot PXD013419 (46). Background distribution in the human proteome based on UniProt (43) and ExoCarta (47) was used as negative control. The percentages of proteins with predicted signal peptide (conventional secretion) and predicted EV-associated proteins (nonconventional secretion) were higher in our samples than in the negative control (Fig. 2*H*). The proportion of predicted EV-associated proteins were similar in untransfected day 0 and samples treated with control siRNA for 72h, and only slightly below the positive control.

In theory, secretomes containing EVs should contain substantial proportion of tryptic peptides corresponding to intracellular or transmembrane domains of membrane proteins. Therefore, we investigated if the predicted percentage of EV-associated proteins contradicts that tryptic peptides in our secretome samples aligned predominantly to the extracellular domains of transmembrane proteins. Positive control experiment PXD013419 had 75.7% of the tryptic peptides aligning to extracellular domains of transmembrane proteins (46). We analyzed an additional study (48), in which EV were isolated with different methods leading to different particle concentration, to obtain more context. High speed centrifugation reference samples had ∼e9 particles/ml and 77.8 ± 1.9% tryptic peptides aligning to extracellular domains, peptide-affinity precipitation samples had ∼e10 particles/ml and 91.5 ± 2.2% tryptic peptides aligning to extracellular domains, and size exclusion chromatography samples had ∼e11 particles/ml and 86.0 ± 1.6% of tryptic peptides aligning to extracellular domains of transmembrane proteins. Interestingly, tryptic peptides were representing CD9, CD81, and CD63 in our secretome samples and the reference EV studies aligned to the extracellular domains, *i.e.,* it was not possible to unambiguously determine from tryptic peptides if these proteins were EV markers or part of the sheddome. We still considered CD9/63/81 as EV markers because they were detected not in isolation but together with other predicted EV-associated proteins. In summary, high percentage of peptides representing extracellular domains did not contradict the prediction of EV-associated proteins. Also, based on external reference data, this metric did not correlate with EV particle concentration.

### Validation of EndoC-βH1 as Human β Cell Model Based On Omics

We compared the transcriptome and cell lysate proteome of EndoC-βH1 both globally and across selected positive control and negative control marker categories to published human primary β cells and insulinomas. The reference human β cell preparations were selected based on purity of the tissue: RNA-seq of adult primary human β cells from seven nondiabetic donors, isolated by FACS sorting with at least 97% β cell purity in GSE67543 (49) and proteomics of four nondiabetic human islet cultures on nanostructured zirconia with 80% ± 10% purity of β cells in PXD007569 (50). The reference insulinoma samples were selected from tumors with typical presentation and without metastases: RNA-seq samples WT_MK27 and WT_MK8 in GSE118014 (51) and proteomics by Song *et al.* (52). The reference transcriptome and proteome contained less molecular species than the EndoC-βH1 transcriptome and proteome acquired in this study. Hence, we calculated percentage of the reference transcriptome/proteome that was recapitulated by EndoC-βH1 cells.

**Fig. 3:**
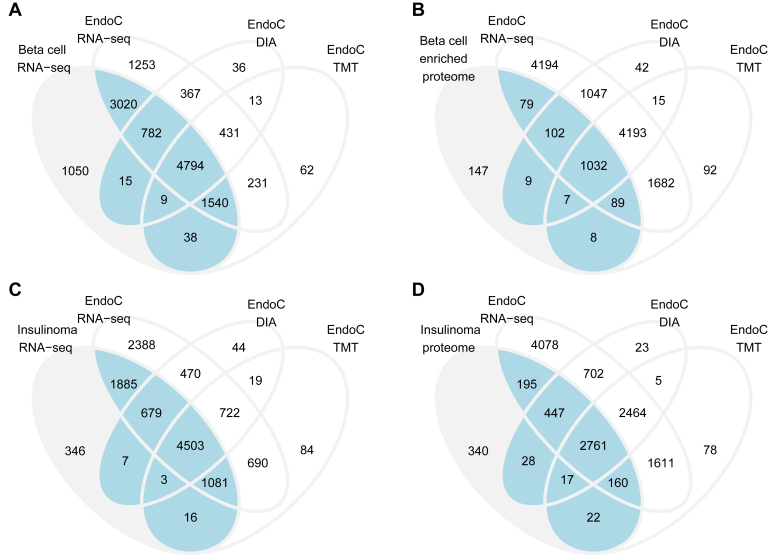
**Transcriptome and proteome of EndoC-βH1 cells compared to adult human β cells and insulinomas.***A*, Venn diagram showing the number of quantified protein-coding genes and proteins in EndoC-βH1 cells *versus* adult nondiabetic β cells (GSE67543). *B*, comparison to human β cell–enriched islet proteomics (PXD007569). *C*, comparison to protein-coding genes with expression >1 TPM in human low-grade insulinomas without lymph node and distant metastases (GSE118014). *D*, comparison to proteome of human insulinomas with typical tumor presentation and without lymph node and distant metastases (52). EndoC-βH1 cells were treated with control nontargeting siRNA and harvested at 72 h. Expression cut-off for genes: median expression ≥ 1 TPM. Expression cut-off for proteins: quantified in ≥ 3 samples and unambiguously annotated. Sample sizes in EndoC-βH1 experiments: RNA-seq N = 3, TMT proteomics = 3, cell lysate DIA proteomics = 12 replicates. *Blue area* indicates protein-coding genes/proteins recapitulated in EndoC-βH1 cells. *Blue + gray area* indicates total number of protein-coding genes/proteins in reference β cell or insulinoma samples. DIA, data-independent acquisition; TMT, tandem mass tag, TPM, transcripts per million.

First, we compared EndoC-βH1 cells to primary human adult β cells. Overall, transcriptome and proteome of EndoC-βH1 cells resembled adult human β cells (Fig. 3, *A* and *B*). EndoC-βH1 recapitulated 90.7% of β cell protein-coding transcriptome and 90% of the β cell-enriched islet proteome. The reference human β cell proteomics data originated from an older study and contained limited number of protein species. Therefore, the agreement between marker gene sets was calculated based on transcriptomics. Good agreement between 69% and 91% *versus* adult β cells was observed across individual gene/protein categories such as β cell markers, genes associated with diabetes, transcription factors, enzymes, ion channels, and lncRNA (supplemental Table S3, supplemental Figs. S3–S8 and S10). The lowest agreement was obtained for G-protein–coupled receptors (GPCRs) with 52% of β cell GPCRs detected on mRNA level and only a few species confirmed by proteomics (supplemental Fig. S9). In particular, median glucagon-like peptide 1 receptor (GLP1R) expression in control siRNA-treated EndoC-βH1 cells was 0.8 TPM, whereas median expression in primary adult β cells was 61.7 TPM. We also verified low GLP1R expression with median 5 TPM in an external experiment with untransfected EndoC-βH1 cells deep-sequenced at 200 million reads per sample in GSE133218 (18).

**Fig. 4:**
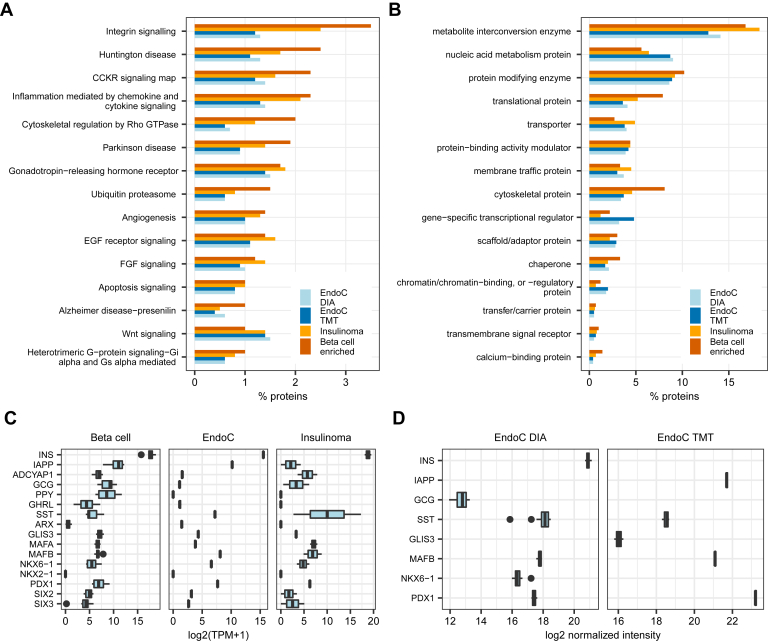
**Selected gene and protein expression in EndoC-βH1 cells compared to β cells and insulinomas.***A*, top 15 PANTHER pathways. *B*, top 15 PANTHER Protein classes. The percentages indicate the number of proteins annotated with a given PANTHER term *versus* the total number of proteins quantified in each experiment. *C*, mRNA expression of endocrine hormones and selected transcription factors. *D*, protein expression of endocrine hormones and selected transcription factors. β cell proteomics from PXD007569 and RNA-seq from GSE67543. Insulinoma proteomics from (52) and RNA-seq from GSE118014. EndoC-βH1 cells were treated with control nontargeting siRNA and harvested at 72 h Expression cutoff for proteins: quantified in ≥ 3 samples and unambiguously annotated. Sample sizes in EndoC-βH1 experiments: RNA-seq N = 3, TMT proteomics = 3, cell lysate DIA proteomics = 12 replicates. DIA, data-independent acquisition; TMT, tandem mass tag.

Interestingly, the EndoC-βH1 transcriptome and proteome were more similar to insulinomas than to primary human β cells (Fig. 3, *C* and *D*). EndoC-βH1 cells recapitulated 96% of the reference insulinoma transcriptome and 91.4% of the reference insulinoma proteome. Improved concordance was also observed for individual protein categories (Fig. 4, *A* and *B*). Overall, the genes and proteins quantified in EndoC-βH1 cells but not in primary β cells were annotated with cell cycle, DNA repair, proliferation, ATP generation, and mitochondrial function Gene Ontology terms. This ectopic expression pattern was consistent with proliferative phenotype. EndoC-βH1 cells double in number every 174 h (14) whereas proliferation of adult human β cells is an extremely rare event (53). EndoC-βH1 expressed β cell hormones INS and IAPP at gene and protein levels and ADCYAP1 (also known as PACAP) at mRNA level (Fig. 4*C*). EndoC-βH1 expressed mRNA of other islet endocrine hormones (Fig. 4*C*) which can also be observed in primary β cells and insulinomas (Figs. 4*C* and S11). However, detection of proglucagon and somatostatin on protein level (Fig. 4*D*) is atypical for adult human β cells. Somatostatin expression in EndoC-βH1 cells has been noted previously (16) and is observed in insulinomas (52). Proglucagon protein was detected based on a single peptide DFINWLIQTK aligning to amino acid positions 166 to 175 and corresponding to glucagon-like peptide 2. Proglucagon co-expression with insulin is a feature of insulinomas (52, 54). EndoC-βH1 cells expressed β cell transcription factors on both gene and protein level (Fig. 4, *C* and *D*). By contrast, α cell transcription factor ARX was detected only on mRNA level and with low expression abundance (Fig. 4*C*). More in-depth evaluation of negative control marker sets indicated no ectopic expression of other α cell markers (supplemental Fig. S12). EndoC-βH1 cells were derived from fetal islet cells at 9 to 11 weeks of gestation (9). Interestingly, expression of endocrine progenitor markers was considerably reduced in EndoC-βH1 cells compared to human fetal pancreas at 9 weeks gestation (supplemental Fig. S13). Expression of NEUROG3 was lost. Markers of fetal β cell at 12 to 18 weeks gestation were not expressed (supplemental Fig. S14). We also observed no ectopic expression of markers of exocrine pancreas acinar (supplemental Fig. S15) and ductal cells (supplemental Fig. S16) and markers of other tissues (supplemental Fig. S17).

### Secretome Composition of EndoC- βH1 Cells

**Fig. 5:**
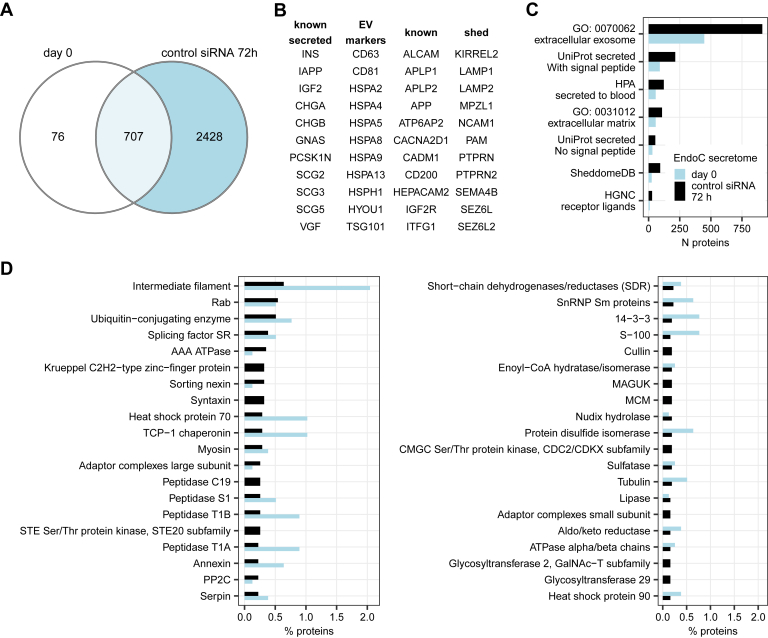
**Composition of the EndoC-βH1 cell secretome.***A*, secretome of untransfected day 0 cells (PXD027920, N = 3) compared to secretome of cells treated with control nontargeting siRNA for 72 h (PXD027913, N = 12). *B*, known positive control proteins that were quantified in EndoC-βH1 cell secretome. *C*, number of proteins by mode of secretion. Proteins can belong to more than one category (*e.g.,* secreted based on both UniProt and HPA annotation). *D*, top 25 PANTHER protein families. Expression cutoff for proteins: quantified in min two out of three untransfected day 0 samples, quantified in min three out of 12 samples at 72 h.

Higher number of proteins were detected in the secretome of EndoC-βH1 cells treated with control nontargeting siRNA than in untransfected day 0 cells (Fig. 5*A*). This observation was true both within the secretome PXD027920 (triplicates per condition) and in comparison to the 12 control secretome samples from PXD027913, *i.e.,* not linked to discrepancies in sample size. As previously shown in Figure 2, these differences were related to transfection with siRNA. Nonetheless, both secretomes of siRNA-stressed and untransfected cells contained four main categories of proteins: (1) known β cell hormones and granins, (2) predicted EV-associated proteins, (3) proteins with signal peptide, and (4) sheddome.

EndoC-βH1 cells secreted known β cell hormones INS and IAPP and autocrine regulator IGF2 (55) (Fig. 5*B*). The secretome contained proteins known to generate bioactive peptides such as all eight proteins of the granin family (Fig. 5*B*), among which chromogranins A and B, secretogranins 2 and 3, and VGF have been previously reported in insulinomas (56). Secretome proteomics indicated potential presence of several bioactive peptides (supplemental Figs. S18–S52) including chromogranin A peptides pancreastatin and vasostatins, chromogranin B peptides GAWK and CCB, and secretogranin-2 peptide manserin and VGF peptides. Some peptides were established β cell secretion products: IGF2 peptide preptin is co-secreted with insulin (57) while VGF peptides neuroendocrine regulatory peptide-2 (58) and TLQP-62 (59) regulate glucose-induced insulin secretion. The secretome also contained CPE, PAM, and proprotein convertases PCSK1 and PCSK2—the enzymes involved in generation of bioactive peptides from prohormones. Interestingly, EndoC-βH1 cells also secreted PCSK9, a member of proprotein convertase family without known enzymatic function but involved in LDL receptor clearance in hepatocytes. We further investigated the function of PCSK9 in EndoC-βH1 cells in a separate follow-up study (20).

We quantified EV-associated markers (40) in the secretome samples—CD63, CD81, heat shock 70 kDa proteins, and TSG101 (Fig. 5*B*) and members of the Rab family (Fig. 5*D*). EV-associated proteins constituted the majority of unique protein species of the secretome, both in untransfected day 0 cells and in cells transfected with control siRNA (Fig. 5*C*). This analysis (Fig. 5*C*) was based on an orthogonal method and different sample subset to previously presented in Figure 2*H*.

The second most common category of proteins in the secretome was classically secreted proteins containing signal peptide (Fig. 5*C*). For example, both untreated and control siRNA-treated EndoC-βH1 cells secreted factors regulating cell differentiation and survival NENF, MANF, MYDGF, and CREG1. EndoC-βH1 cells also secreted proteins that are measurable in circulation (60) such as annexins, apolipoproteins, serpins, IGF-binding proteins, and VEGFA.

In addition, we captured known proteins with shed extracellular domain in islets or rodent cell lines (22, 61, 62) in the EndoC-βH1 cell secretome (Fig. 5*B*). Sheddases ADAM10, ADAM17, ADAM22, and ADAM9 were quantified in the EndoC-βH1 secretome. Sheddases BACE1 and BACE2 were not quantified in the secretome samples but were quantified in the DIA and TMT cell lysate experiments.

Finally, we investigated the most abundantly secreted proteins in the untransfected day 0 cells (supplemental Table S4) and in control siRNA-treated samples (Table 1). β cell hormones were among the most abundant secreted proteins confirming β cell line identity.

**Table 1:** Top 150 secreted proteins

Rank	UniProt	Symbol	Rank	UniProt	Symbol	Rank	UniProt	Symbol
1	Q5TEC6	H3-2	51	P62851	RPS25	101	P07237	P4HB
2	Q9H161	ALX4	52	P13533	MYH6	102	O43405	COCH
3	Q6ZNA1	ZNF836	53	Q6ZVC0	NYAP1	103	P62249	RPS16
4	Q96JB3	HIC2	54	P30049	ATP5F1D	104	P11142	HSPA8
5	P25398	RPS12	55	P60660	MYL6	105	Q9Y2S6	TMA7
6	P61769	B2M	56	Q92820	GGH	106	Q16769	QPCT
7	O75037	KIF21B	57	Q06830	PRDX1	107	P60900	PSMA6
8	Q8N4Y2	CRACR2B	58	P06733	ENO1	108	Q8IWT0	ZBTB8OS
9	P01308	INS	59	P28072	PSMB6	109	Q15084	PDIA6
10	P10997	IAPP	60	Q96M02	C10ORF90	110	P30040	ERP29
11	P05386	RPLP1	61	P01857	IGHG1	111	O43617	TRAPPC3
12	P04080	CSTB	62	Q9Y2V2	CARHSP1	112	P49458	SRP9
13	Q99797	MIPEP	63	Q9UHG2	PCSK1N	113	P40926	MDH2
14	P00441	SOD1	64	Q16555	DPYSL2	114	Q05519	SRSF11
15	P13473	LAMP2	65	P61088	UBE2N	115	P09651	HNRNPA1
16	P06753	TPM3	66	P62937	PPIA	116	P06703	S100A6
17	Q8NGI1	OR56B2P	67	Q13522	PPP1R1A	117	Q8WXD2	SCG3
18	Q14011	CIRBP	68	Q14103	HNRNPD	118	O00264	PGRMC1
19	Q13795	ARFRP1	69	Q9BVA1	TUBB2B	119	Q9Y5S9	RBM8A
20	P68133	ACTA1	70	P23528	CFL1	120	P26641	EEF1G
21	P63279	UBE2I	71	P23284	PPIB	121	P61956	SUMO2
22	P02790	HPX	72	P55145	MANF	122	P29401	TKT
23	Q92526	CCT6B	73	P30046	DDT	123	P30044	PRDX5
24	P14854	COX6B1	74	Q8NBP7	PCSK9	124	Q9NXJ5	PGPEP1
25	P00352	ALDH1A1	75	P16870	CPE	125	Q9Y3C8	UFC1
26	P02766	TTR	76	P41567	EIF1	126	P62304	SNRPE
27	Q96FJ2	DYNLL2	77	P01859	IGHG2	127	O43598	DNPH1
28	P10599	TXN	78	P01034	CST3	128	Q00688	FKBP3
29	Q9H3M0	KCNF1	79	P63261	ACTG1	129	Q99729	HNRNPAB
30	P20674	COX5A	80	P23526	AHCY	130	P16401	H1-5
31	P07737	PFN1	81	P35030	PRSS3	131	P31946	YWHAB
32	P62318	SNRPD3	82	Q9H299	SH3BGRL3	132	P07305	H1-0
33	P62805	H4C1	83	Q9BUJ0	ABHD14A	133	Q9NQG5	RPRD1B
34	O75093	SLIT1	84	O95336	PGLS	134	Q9NPF5	DMAP1
35	P07108	DBI	85	Q9GZX9	TWSG1	135	P62857	RPS28
36	P32119	PRDX2	86	P07900	HSP90AA1	136	P35527	KRT9
37	P61457	PCBD1	87	Q15631	TSN	137	Q9BRA2	TXNDC17
38	P26583	HMGB2	88	Q13526	PIN1	138	P68036	UBE2L3
39	Q04760	GLO1	89	Q13404	UBE2V1	139	P25789	PSMA4
40	P60866	RPS20	90	P62316	SNRPD2	140	P63173	RPL38
41	P00492	HPRT1	91	P62861	FAU	141	P04075	ALDOA
42	P24666	ACP1	92	Q9Y5Q9	GTF3C3	142	O94985	CLSTN1
43	P60174	TPI1	93	Q15819	UBE2V2	143	O75367	MACROH2A1
44	P25391	LAMA1	94	P30101	PDIA3	144	O76038	SCGN
45	P09936	UCHL1	95	Q15413	RYR3	145	P43251	BTD
46	P10645	CHGA	96	Q92520	FAM3C	146	P07205	PGK2
47	P62942	FKBP1A	97	Q08380	LGALS3BP	147	O00299	CLIC1
48	P62258	YWHAE	98	Q8IUH3	RBM45	148	P55327	TPD52
49	Q86TJ2	TADA2B	99	Q9UJ72	ANXA10	149	Q14061	COX17
50	Q92688	ANP32B	100	P63104	YWHAZ	150	P30041	PRDX6

Proteins are ordered by decreasing median normalized expression abundance in EndoC-βH1 cells treated with control nontargeting siRNA at 72 h in the DIA secretome experiment (PXD027913). N = 12.

## Discussion

All β cell models have advantages and limitations (reviewed in detail in (13)). EndoC-βH1 cells have the advantage of being a pure cell population of human origin, which can be proliferated to yield large numbers of cells needed for *in vitro* and omics experiments. Unsurprisingly, EndoC-βH1 cells are among the most commonly used human β cell lines for early-stage diabetes research. Yet, despite extensive functional, morphological, and electrophysiological characterization (13), EndoC-βH1 cells have not been validated as an *in vitro* human β cell model based on omics.

Here, we confirm that EndoC-βH1 are cells of β cell lineage using transcriptomics and proteomics data. The observed discrepancies with adult human β cells and increased similarity with insulinomas were due to elevated proliferation rate (14). Therefore, inducible growth arrest will likely ameliorate the observed discrepancies in gene and protein expression pattern. In fact, next-generation EndoC-βH2 and EndoC-βH3 cells display a dramatic increase in insulin content and secretion capacity after the immortalization cassette is excised (10, 11). Increased expression of β cell markers postexcision has also been shown in EndoC-βH2 cells (10).

The limited detection of GPCRs in our proteomics experiments might be of technical nature. In general, membrane proteins are hydrophobic, but the lysis buffers used in these proteomics experiments are likely not harsh enough to solubilize GPCRs. In addition, the hydrophobic nature of GPCRS leads to low frequency of polar amino acids (*i.e.,* very few lysin and arginine residues), which affects tryptic digestion and subsequent detection by mass spectrometry (63, 64). By contrast, low GLP1R expression appeared to be a true biological finding based on our data and validation in GSE133218. GLP1R expression may also be remedied by using next-generation cell lines. For example, improved response to GLP-1 mimetics has been reported by vendors in EndoC-βH5 cells (https://www.humancelldesign.com/human-beta-cells-endoc-bh5/).

In summary, growth-arrested next-generation EndoC cells may be more suitable for some research questions such as studies of factors influencing proliferation of adult β cells, GLP1R-mediated drug delivery, or effects of GLP-1 mimetics. We believe that systematic comparison of growth-arrested and nongrowth–arrested next generation EndoC-βH2 to EndoC-βH5 cells to primary human β cells constitutes an important direction of future research.

As EndoC-βH1 cells proved to be a reasonable translational human β cell model based on our omics analysis and prior functional studies (9, 13, 14, 15, 16), we used EndoC-βH1 cells to uncover the β cell secretome. EndoC-βH1 cells secreted β cell hormones INS and IAPP. Both adult human β cells and insulinoma cells secrete low amounts of insulin under basal conditions and not only after glucose or KCl challenge (65, 66). Hence, presence of insulin and co-secreted products IAPP and IGF2/preptin in the secretome of unstimulated EndoC-βH1 is not abnormal.

Our secretome proteomics workflow was aimed at identification of secreted proteins and contained a tryptic digestion step. Thus, we could not establish full-length sequences of endogenously cleaved peptides. However, we could make inference based on the spectra and prior knowledge of protein families generating bioactive peptides. Our secretome data indicated that β cells may be capable of producing and secreting bioactive peptides. In fact, we confirmed the presence of preptin, neuroendocrine regulatory peptide-2, and TLQP-62 (57, 58, 59) in conditioned medium from EndoC-βH1 cells. We also found that EndoC-βH1 cells secrete proteins of the granin family and possess the enzymatic machinery necessary to produce bioactive peptides, which suggests that the endogenous bioactive peptide repertoire of β cells might be richer than previously anticipated. Bioactive peptides may play an important role in both autocrine and endocrine signaling as well as crosstalk between β cells and other islets cell types. We believe that presence of full-length endogenous peptides and their secretion dynamics and function warrant further investigation in subsequent studies with specialized proteomics methods.

The secretome of EndoC-βH1 encompassed classically secreted proteins with signal peptide. However, we speculate that these secreted proteins may play a limited role as biomedically relevant biomarkers. Only a few proteins in the secretome had enriched expression in β cells (INS, IAPP) or nervous system and β cells (*e.g.* PTPRN, VGF) (67). In addition, the relative contribution of β cells to the plasma pool of broadly expressed proteins in human is unknown, and deconvoluting tissue-of-origin for such blood-borne biomarkers can be extremely challenging. By contrast, classically secreted proteins may play an autocrine or paracrine function. For example, the functions of NENF, MANF, MYDGF, and CREG1 as regulators of cell survival and differentiation were described in a different tissue context (43), but they may play similar roles in islets. In fact, MANF has been recently characterized as a β cell protecting factor (68).

Proteins, that are typically annotated as intracellular, can be found in conditioned media either due to unconventional secretion *via* EV or because of leakage. Computational predictions with two methods indicated high proportion of EV-associated protein species in both untransfected day 0 cells (not stressed) and cells treated with control siRNA (a nonspecific stressor). It was an unexpected finding because we did not specifically enrich for EV in our secretome proteomics workflow. However, we confirmed lack of effect of siRNA transfection on proliferation, apoptosis, and β cell markers defining the cell identity. Secreted apoptosis-associated proteins accounted for 0% to <2% of the protein intensities in the secretome of untransfected cells and cells treated with control siRNA, respectively. Furthermore, abundances of proteins annotated as cytoplasmic in UniProt did not correlate between paired cell lysate and secretome samples (PXD027911 *versus* PXD027913). Therefore, leakage was unlikely.

EVs are released from the plasma membrane *via* exocytosis. β cells store insulin in intracellular vesicles called insulin granules and secrete insulin by transporting insulin granules to the plasma membrane and subsequent exocytosis (69). However, it was not clear from current literature if the content of insulin granules is spilled into the extracellular space or if the granules retain membrane and persist as EVs after they are secreted. The proteomics studies on insulin granules isolated from whole-cell rodent β cell lines report only 50 to 140 proteins (70) and do not correlate with the repertoire of EV-associated proteins in our secretome samples. Therefore, we speculate that our secretome preparations represented the total EndoC-βH1 secretome including a diluted EV fraction that is not identical to insulin granules.

EV may offer additional opportunities to discover and noninvasively monitor biomarkers reflecting β cell state. Therefore, future studies need to further elucidate composition of β cell EV with dedicated EV isolation and proteomics workflows, to establish EV secretion dynamics and if it is possible to enrich β cell derived EV from plasma, serum, or fecal samples based on additional biomarkers.

## Data Availability

RNA-seq data have been deposited to NCBI GEO (71) with accession GSE182016.

The mass spectrometry proteomics data have been deposited to the ProteomeXchange Consortium *via* the PRIDE (72) partner repository with the following identifiers: PXD027898, PXD027921, PXD027920, PXD027911, and PXD027913. Protein quantification tables for each experiment and a summary table are provided as Supplemental Data in this article.

The previously published datasets used as reference primary human biological material were GTEx (67), Human Protein Atlas (HPA) (60), GSE67543 (49), GSE57973 (73), GSE118014 (51), GSE84133 (74), GSE81608 (75), E−MTAB−5061 (76), GSE86469 (77), PXD007569 (50), and PXD013419 (46). Marker lists were obtained from HGNC (45), OMIM (78), GENECODE v36 (79) and supplementary datasets in (44, 52, 80, 81, 82, 83).

## Supplemental data

This article contains supplemental data.

## Conflict of interest

S. A., K. Se., M. R., A. J., C. R. U., and G. H. are employed by AstraZeneca. R. S. is a shareholder in and a consultant for Univercell-Biosolutions.

## Supplemental Data

Supplemental Tables S1–S4 and Figures S1–S52

PXD027898_TMT_lysate_siCtrl_vs_untreated

PXD027911_DIA_lysate_12x12

PXD027913_DIA_secretome_12x12

PXD027920_DIA_secretome_timecourse

PXD027921_TMT_lysate_3x3

summaryTable
