# Supplementary material for: Characterization of the Secretome, Transcriptome, and Proteome of Human β Cell Line EndoC-βH1
Source: Mol Cell Proteomics. 2022 Apr 2;21(5):100229. doi: 10.1016/j.mcpro.2022.100229 (PMC9062487; doi:10.1016/j.mcpro.2022.100229)
Supplement: Supplemental Tables S1–S4 and Figures S1–S52 [file mmc1.pdf]

Table S1. Data sets generated in this study. The table shows the number of independent cell culture ("biological") replicates per condition. $^{+}$ indicates paired lysate and secretome samples generated from the same cells.

PRIDE	Material	Type	Total N	Untransfected	siCtrl			siPCSK9		
				0h	24h	48h	72h	24h	48h	72h
GSE182016	cell lysate	RNA-seq	6				3			3
PXD027921	cell lysate	TMT	6				3			3
PXD027898	cell lysate	TMT	21	3	3	3	3	3	3	3
PXD027920	secretome	DIA	21	3	3	3	3	3	3	3
PXD027913^{+}	secretome	DIA	24				12			12
PXD027911^{+}	cell lysate	DIA	24				12			12

Table S2. Primer sequences for RT-qPCR.

Gene	Forward primer	Reverse primer
INS	TGTCCTTCTGCCATGGCCCT	TTCACAAAGGCTGCGGCTGG
PDX1	TACTGGATTGGCGTTGTTTGTGGC	AGGGAGCCTTCCAATGTGTATGGT
NKX6-1	GCCCTGGGAGAAGACTTTCGAACAA	TTCTGGAACAGACCTTGACCTGA
KI67	AGCACCAGAGGAAATTGTGGAGGA	ATGATGACCACGGGTTCGGATGAT
CYCLOPHILIN-A	ATGGCAAATGCTGGACCCAACA	ACATGCTTGCCATCCAACCACT

Figure S1. Gating strategy for FACS in Figure 1A-C.

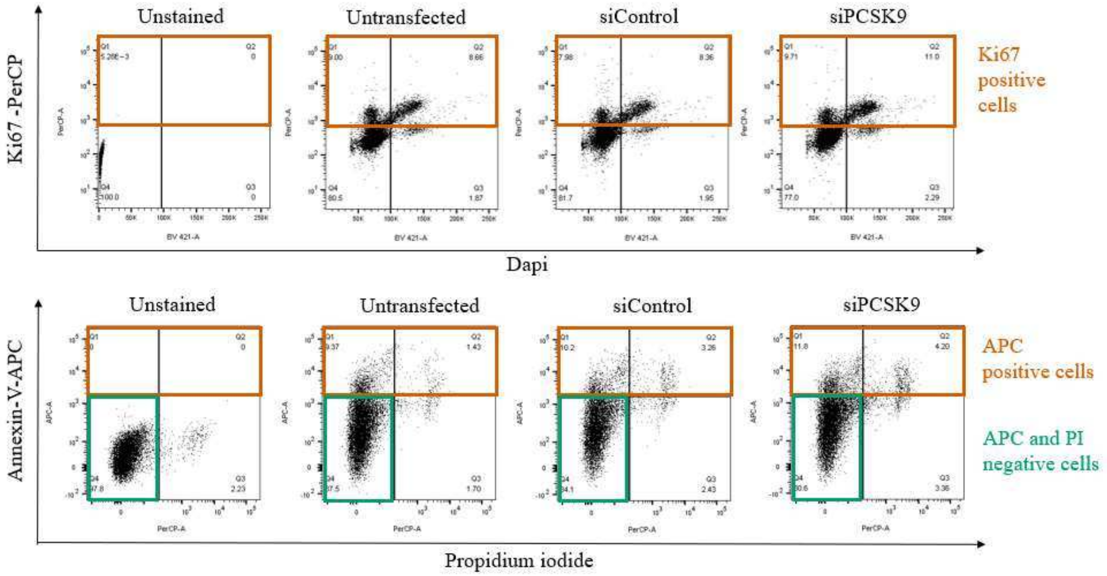

Fig. S2. Disambiguation of proteins that could be either human or mouse based on sequence similarity. The figure shows representative analysis in paired-sample cell lysate PXD027911 and secretome PXD027913 experiments. **(A)** Overlap with known Matrigel composition. **(B)** Distribution of missing values. **(C-D)** Distribution of normalized protein intensities. One curve corresponds to one sample. Vertical grey lines indicate median and interquartile range for human-unique protein groups.

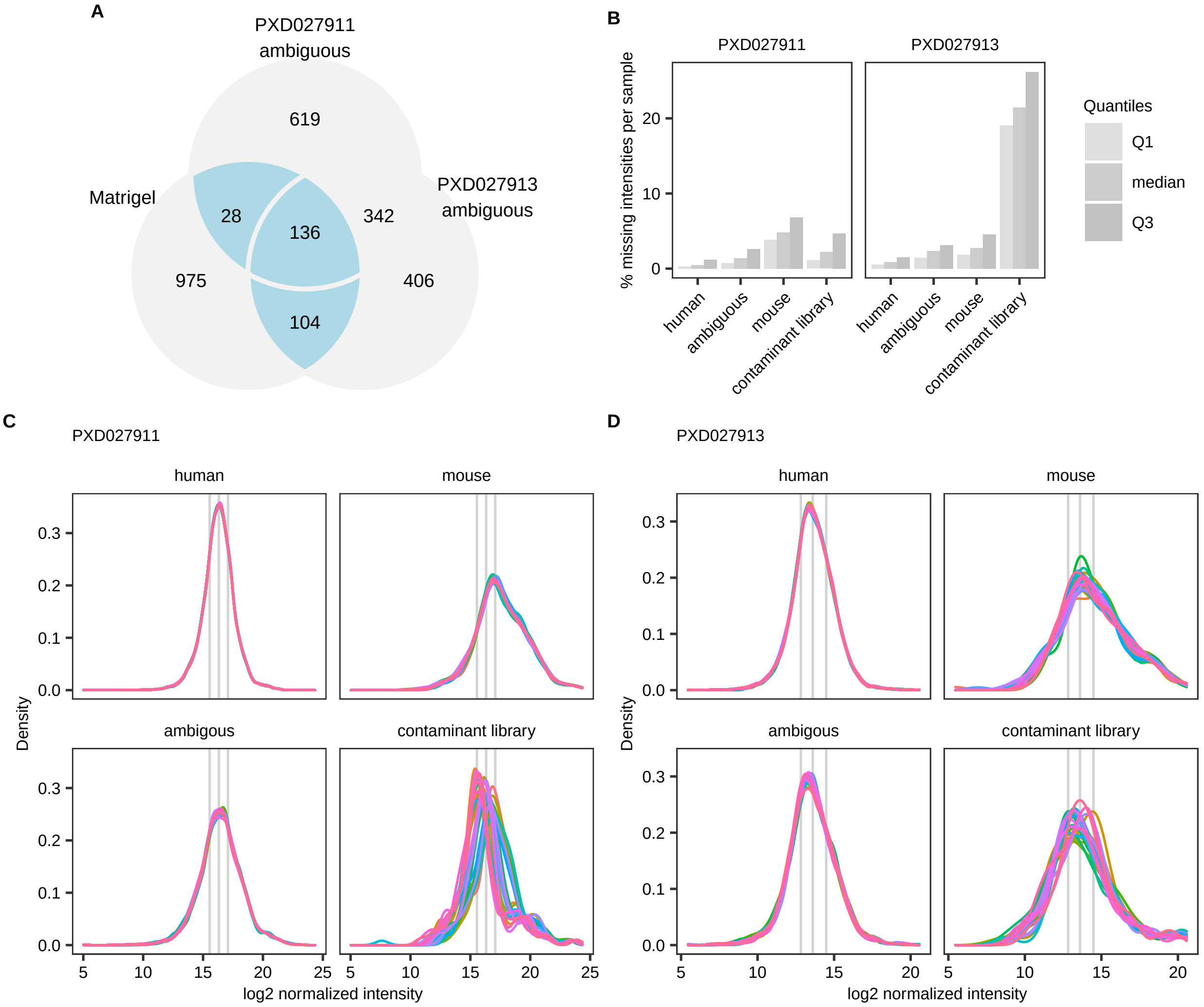

Table S3. Detectability of expression across gene categories. The table shows the number of markers with median expression >= 1 TPM in adult β cells (GSE67543, N=7, FACS sorted, at least 97% purity) and EndoC-βH1 cells. Percentage is given as the number of markers expressed above the threshold in both EndoC-βH1 cells and adult β cells vs in adult β cells. Total N markers is the total number of genes in the gene category. E.g., total number of human enzymes according to HGNC (December 2020).

Category	Total N markers	Adult β cells	EndoC	Both	Percent
β cell markers	128	128	116	116	90.62
OMIM MODY and/or familial hyperinsulinemic hypoglycemia	19	10	10	9	90
GWAS T2D or glycemic traits	56	41	37	32	78.05
Transcription factors	1639	1035	1104	932	90.05
Enzymes	1797	1240	1339	1131	91.21
Ion channels	328	126	134	99	78.57
GPCRs	1421	120	115	62	51.67
Polyadenylated lncRNA	2042	198	442	137	69.19

Fig. S3. β **cell markers.** Markers were defined based on elevated expression Z-score in β cells vs other cell types in at least two out of four single-cell RNA-seq studies GSE84133, GSE81608, E-MTAB-5061 and GSE86469. **(A)** Gene expression in adult β cells (GSE67543, N=7, FACS sorted, at least 97% purity). Markers are ordered by decreasing median expression. **(B)** Gene expression in EndoC-βH1 cells treated with control siRNA at 72 hours (N=3). **(C)** Protein expression in DIA proteomics on cell lysate of EndoC-βH1 cells treated with control siRNA at 72 hours (N=12). **(D)** Protein expression in TMT proteomics on cell lysate of EndoC-βH1 cells treated with control siRNA at 72 hours (N=3). No boxplot for a protein in panels C and D indicates that the protein was either not detected or quantified in < 3 samples.

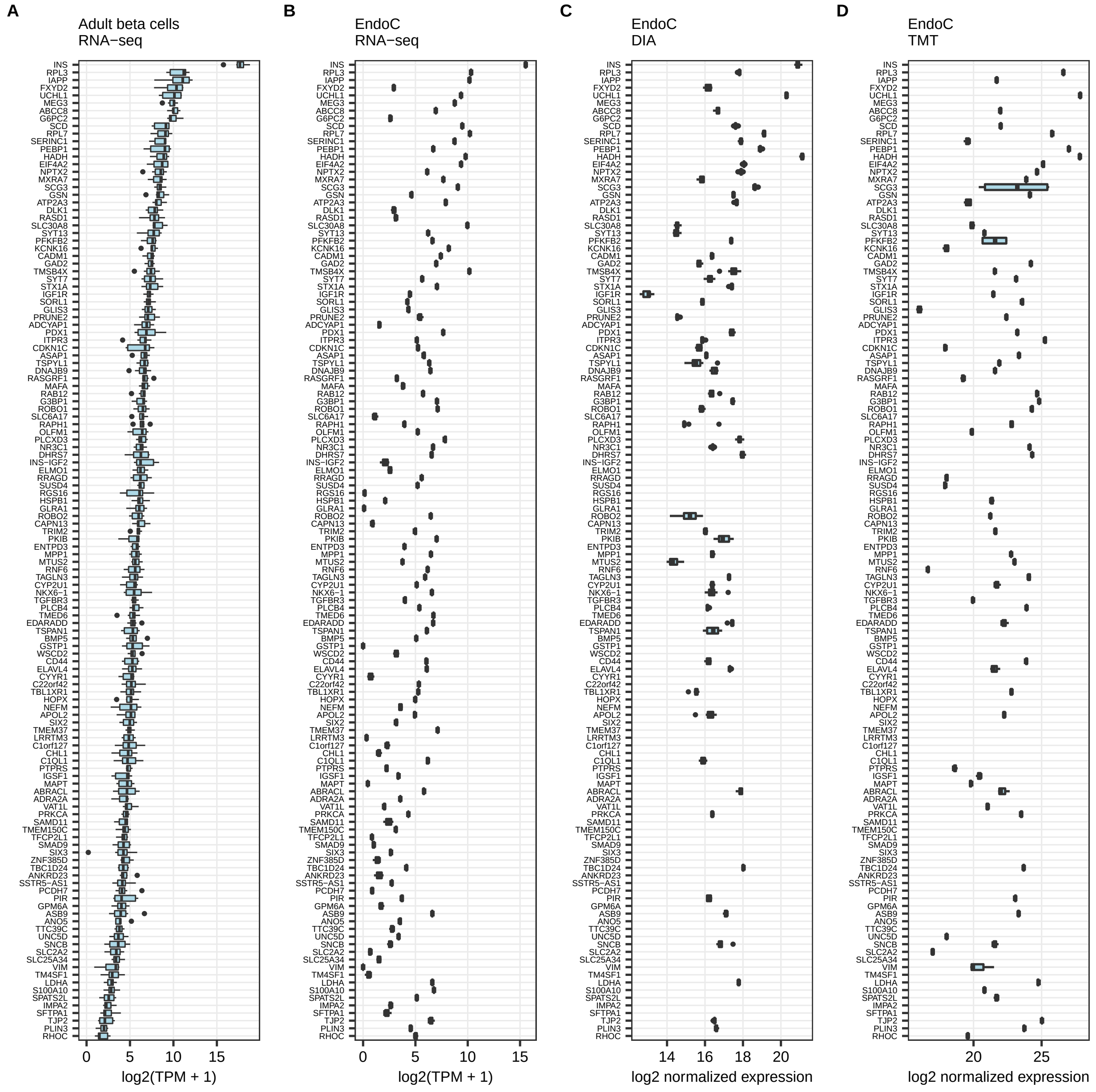

Fig. S4. Genes implicated in maturity-onset diabetes of the young and/or familial hyperinsulinemic hypoglycemia (OMIM). (A) Gene expression in adult β cells (GSE67543, N=7, FACS sorted, at least 97% purity). **(B)** Gene expression in EndoC-βH1 cells treated with control siRNA at 72 hours (N=3). **(C)** Protein expression in DIA proteomics on cell lysate of EndoC-βH1 cells treated with control siRNA at 72 hours (N=12). **(D)** Protein expression in TMT proteomics on cell lysate of EndoC-βH1 cells treated with control siRNA at 72 hours (N=3). No boxplot for a protein in panels C and D indicates that the protein was either not detected or quantified in $<$ 3 samples.

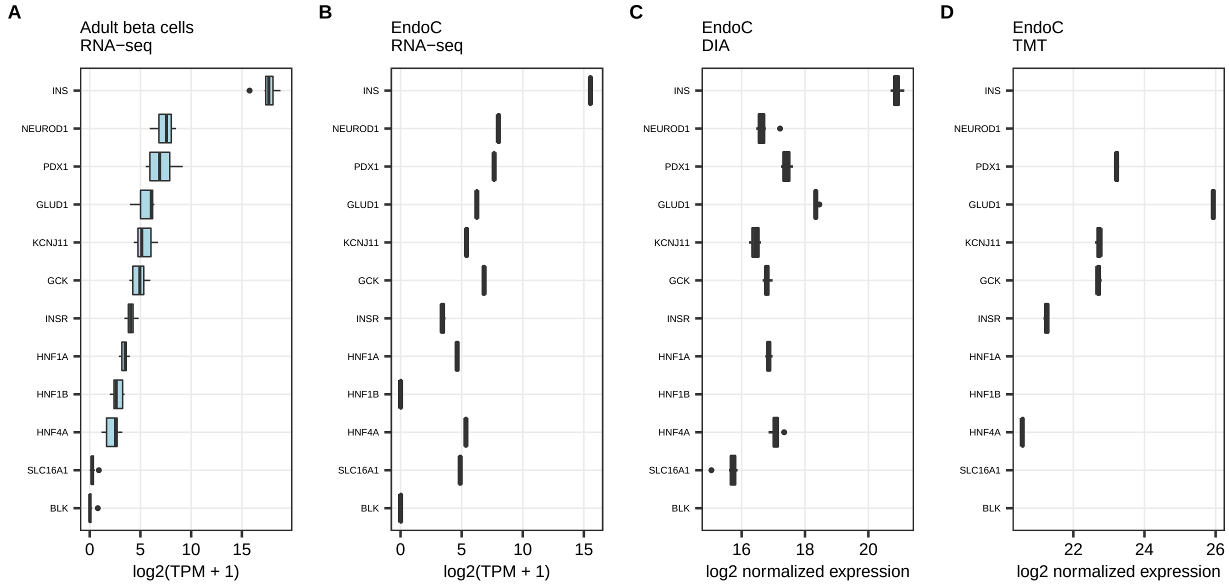

Fig. S5. GWAS genes associated with T2D and glycemic traits. Markers were extracted from Supplementary Data 15 in Viñuela et al. 2020 (GWAS-to-eQTL co-localization in islets). **(A)** Gene expression in adult β cells (GSE67543, N=7, FACS sorted, at least 97% purity). **(B)** Gene expression in EndoC-βH1 cells treated with control siRNA at 72 hours (N=3). **(C)** Protein expression in DIA proteomics on cell lysate of EndoC-βH1 cells treated with control siRNA at 72 hours (N=12). **(D)** Protein expression in TMT proteomics on cell lysate of EndoC-βH1 cells treated with control siRNA at 72 hours (N=3). No boxplot for a protein in panels C and D indicates that the protein was either not detected or quantified in $<$ 3 samples.

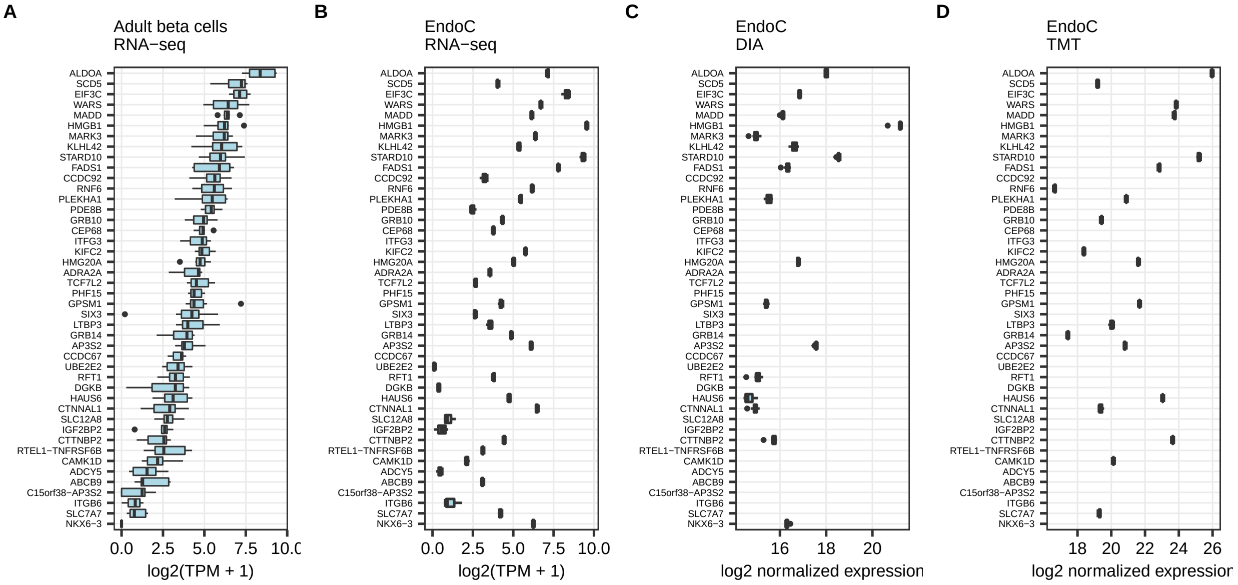

Fig. S6. Transcription factors. Genes were extracted from supplementary data in Lambert et al. 2018. **(A)** Gene expression in adult β cells (GSE67543, N=7, FACS sorted, at least 97% purity). Top 130 genes by median expression. **(B)** Gene expression in EndoC-βH1 cells treated with control siRNA at 72 hours (N=3). **(C)** Protein expression in DIA proteomics on cell lysate of EndoC-βH1 cells treated with control siRNA at 72 hours (N=12). **(D)** Protein expression in TMT proteomics on cell lysate of EndoC-βH1 cells treated with control siRNA at 72 hours (N=3). No boxplot for a protein in panels C and D indicates that the protein was either not detected or quantified in $<$ 3 samples.

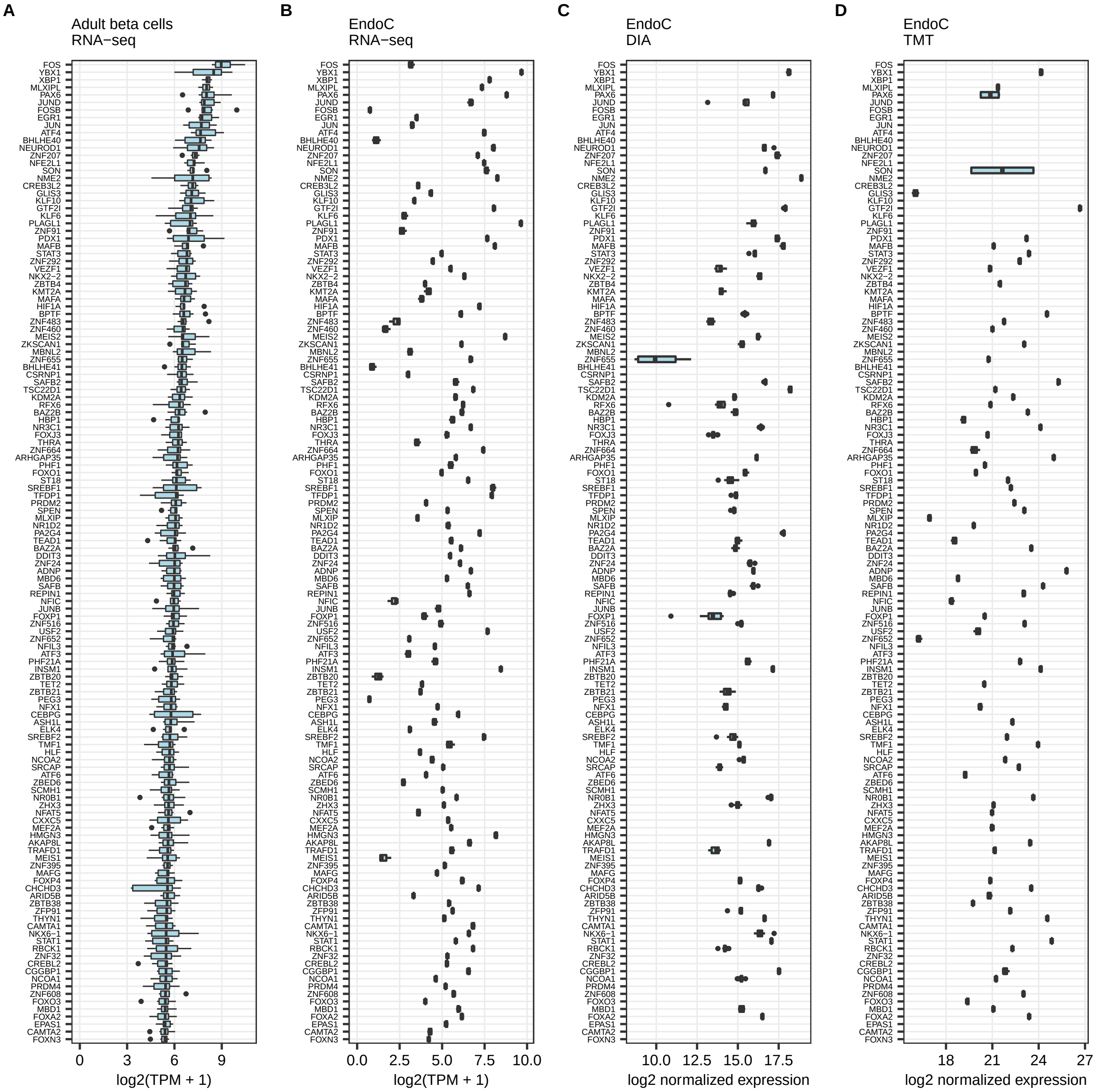

Fig. S7. Enzymes. Gene list was obtained from HGNC. **(A)** Gene expression in adult β cells (GSE67543, N=7, FACS sorted, at least 97% purity). Top 130 genes by median expression. **(B)** Gene expression in EndoC-βH1 cells treated with control siRNA at 72 hours (N=3). **(C)** Protein expression in DIA proteomics on cell lysate of EndoC-βH1 cells treated with control siRNA at 72 hours (N=12). **(D)** Protein expression in TMT proteomics on cell lysate of EndoC-βH1 cells treated with control siRNA at 72 hours (N=3). No boxplot for a protein in panels C and D indicates that the protein was either not detected or quantified in $<$ 3 samples.

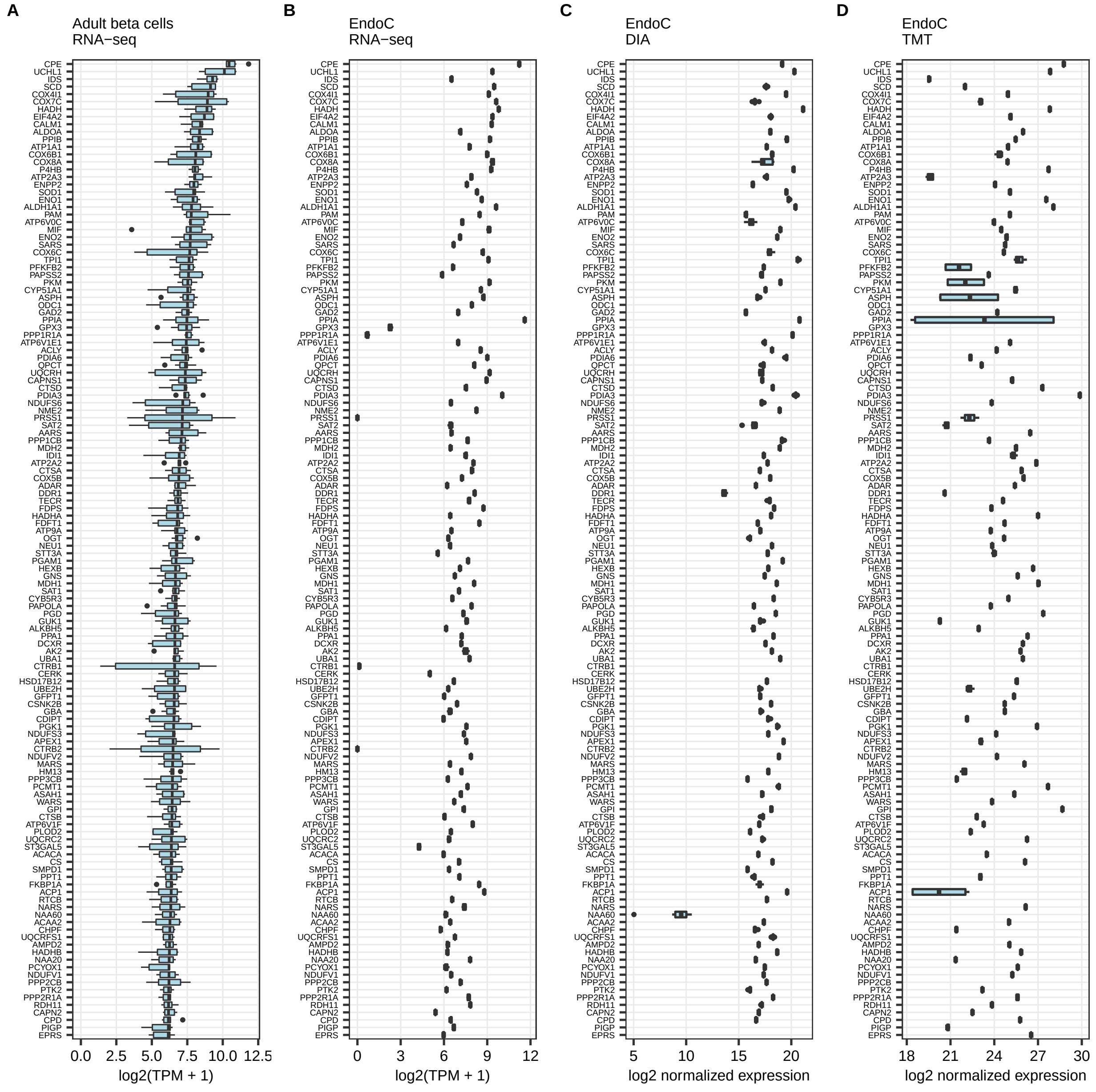

Fig. S8. Ion channels. Gene list was obtained from HGNC. **(A)** Gene expression in adult β cells (GSE67543, N=7, FACS sorted, at least 97% purity). **(B)** Gene expression in EndoC-βH1 cells treated with control siRNA at 72 hours (N=3). **(C)** Protein expression in DIA proteomics on cell lysate of EndoC-βH1 cells treated with control siRNA at 72 hours (N=12). **(D)** Protein expression in TMT proteomics on cell lysate of EndoC-βH1 cells treated with control siRNA at 72 hours (N=3). No boxplot for a protein in panels C and D indicates that the protein was either not detected or quantified in $<$ 3 samples.

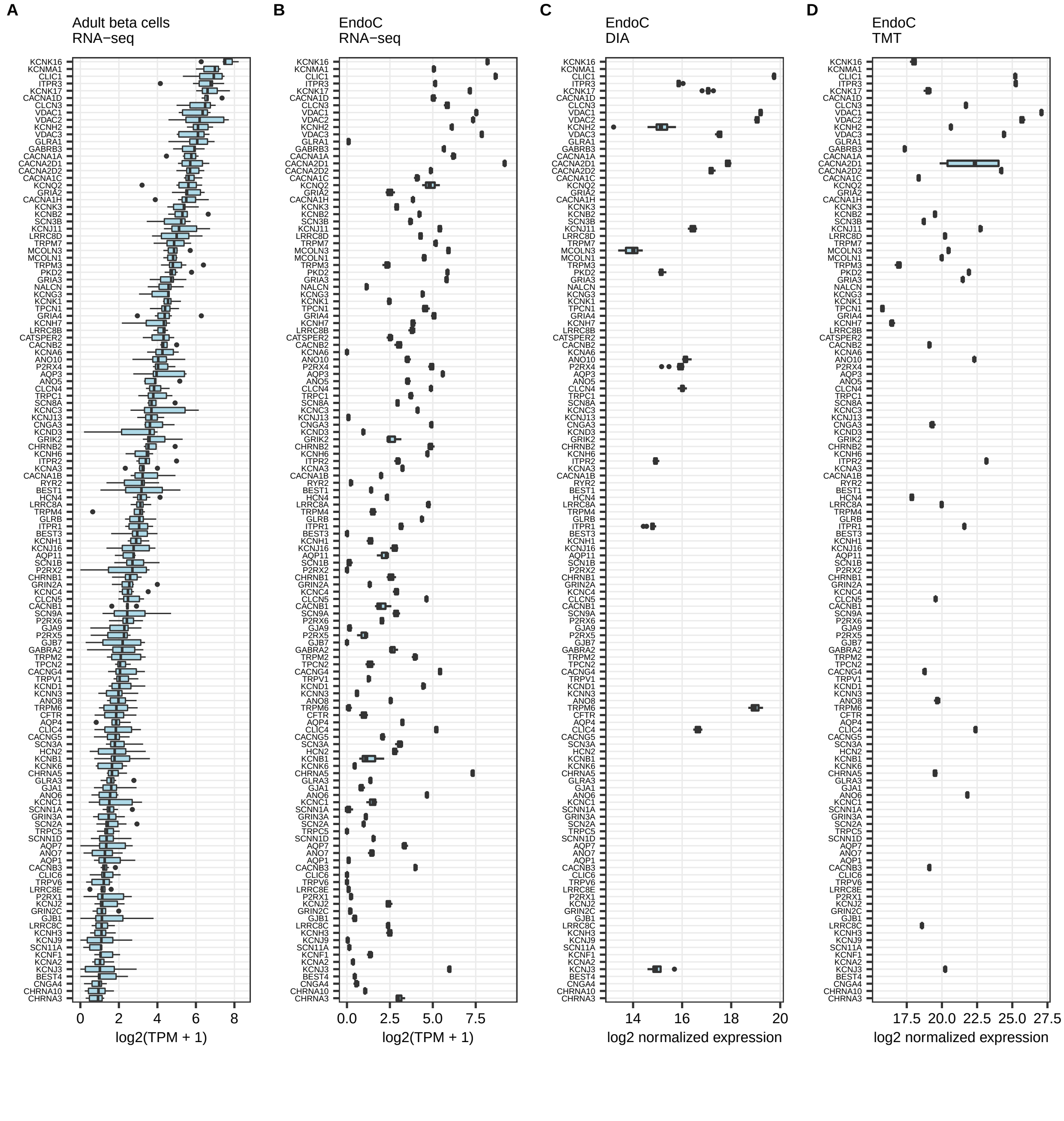

Fig. S9. G protein-coupled receptors. Gene list was obtained from HGNC. **(A)** Gene expression in adult β cells (GSE67543, N=7, FACS sorted, at least 97% purity). **(B)** Gene expression in EndoC-βH1 cells treated with control siRNA at 72 hours (N=3). **(C)** Protein expression in DIA proteomics on cell lysate of EndoC-βH1 cells treated with control siRNA at 72 hours (N=12). **(D)** Protein expression in TMT proteomics on cell lysate of EndoC-βH1 cells treated with control siRNA at 72 hours (N=3). No boxplot for a protein in panels C and D indicates that the protein was either not detected or quantified in < 3 samples.

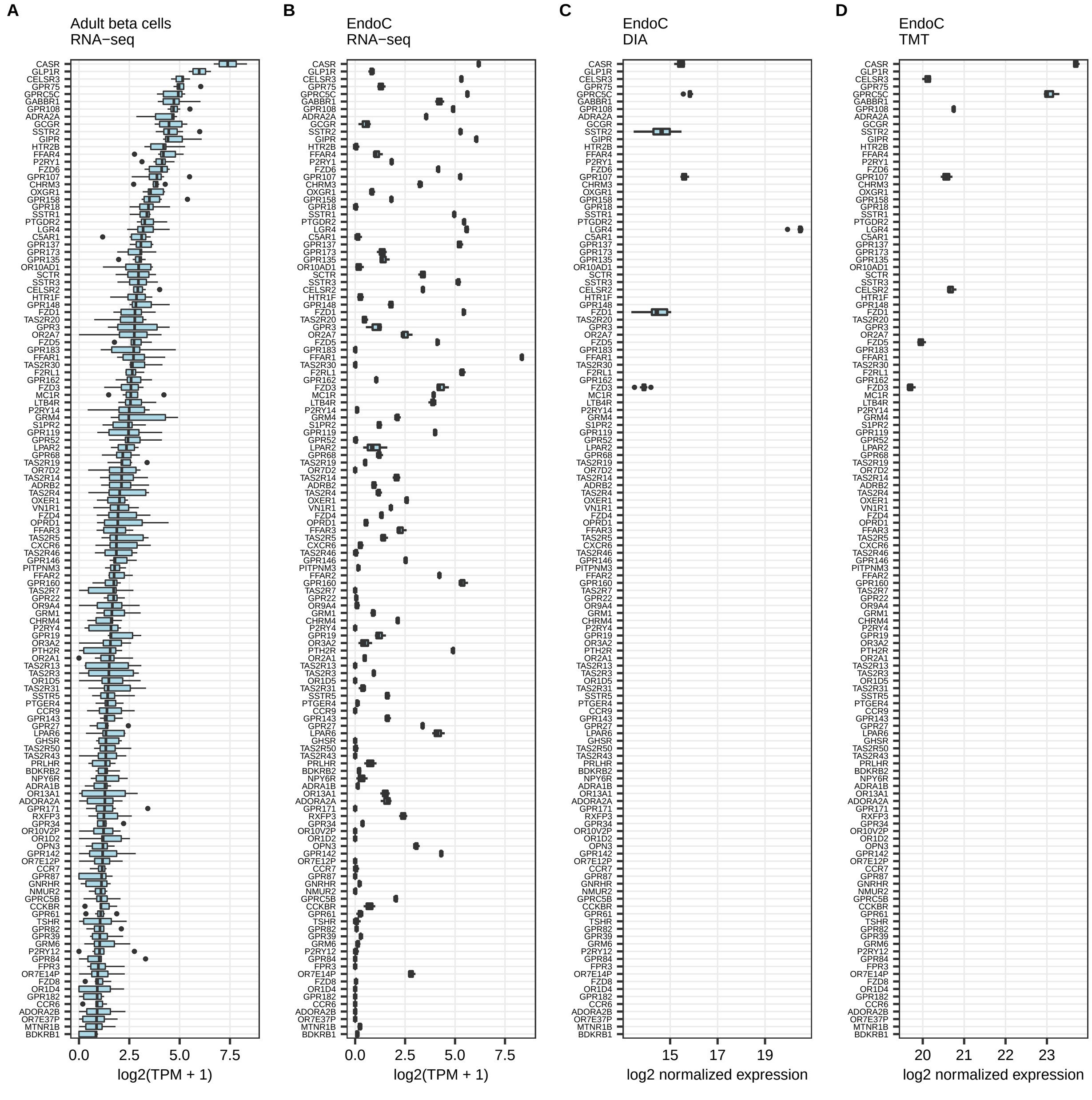

Fig. S10. Polyadenylated lncRNA. Gene list was obtained by cross-referencing HGNC with GENECODE v36 polyadenylation features. **(A)** lncRNA with median expression $>=$1 TPM in adult β cells (GSE67543, N=7, FACS sorted, at least 97% purity). **(B)** Expression of the corresponding genes in EndoC-βH1 cells treated with control siRNA at 72 hours (N=3).

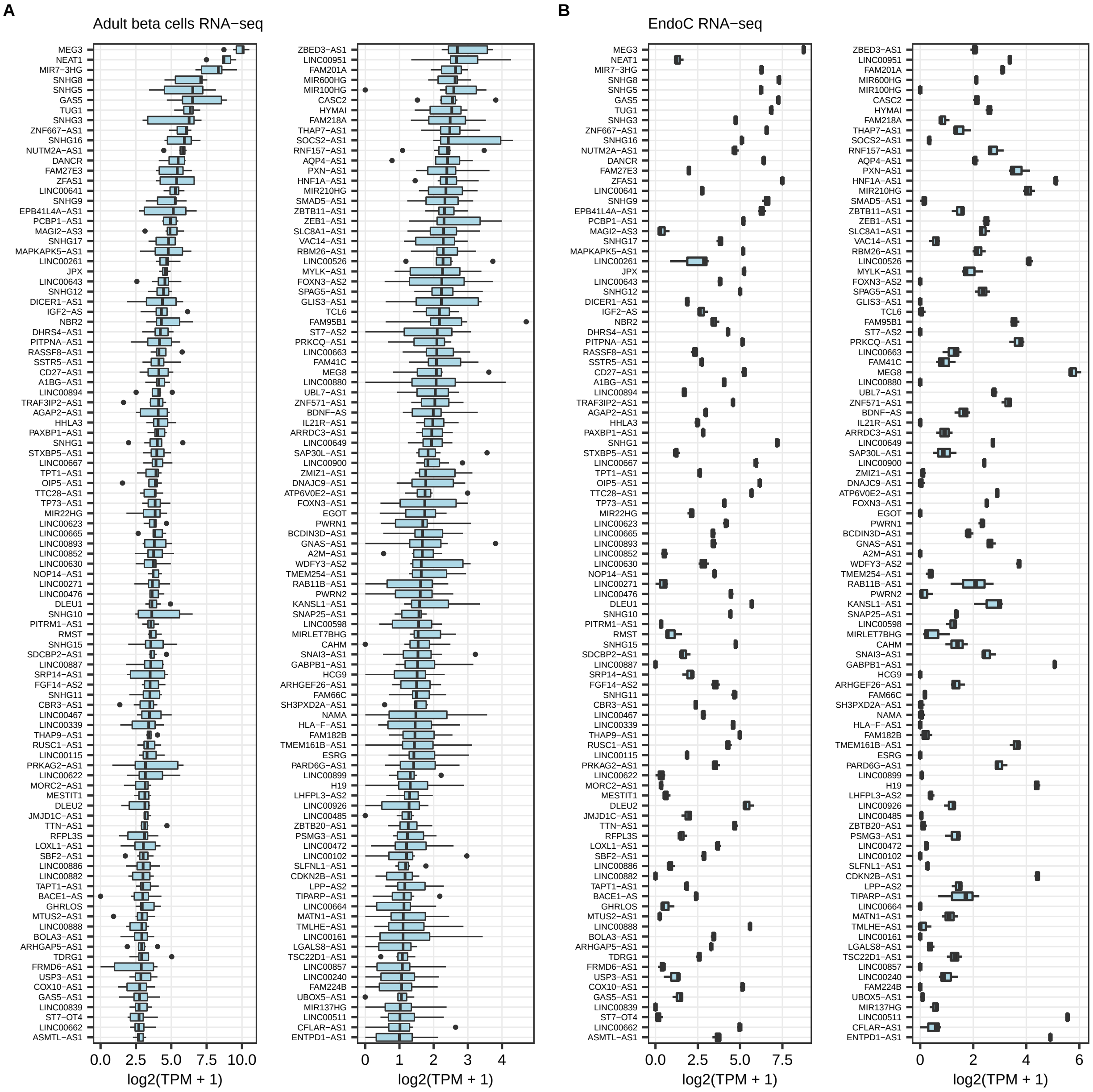

Fig. S11. Glucagon and somatostatin expression compared to insulin in single-cell RNA seq studies on human islets. Cells from non-diabetic donors in **(A)** GSE84133, **(B)** GSE81608, **(C)** E-MTAB-5061 and **(D)** GSE86469.

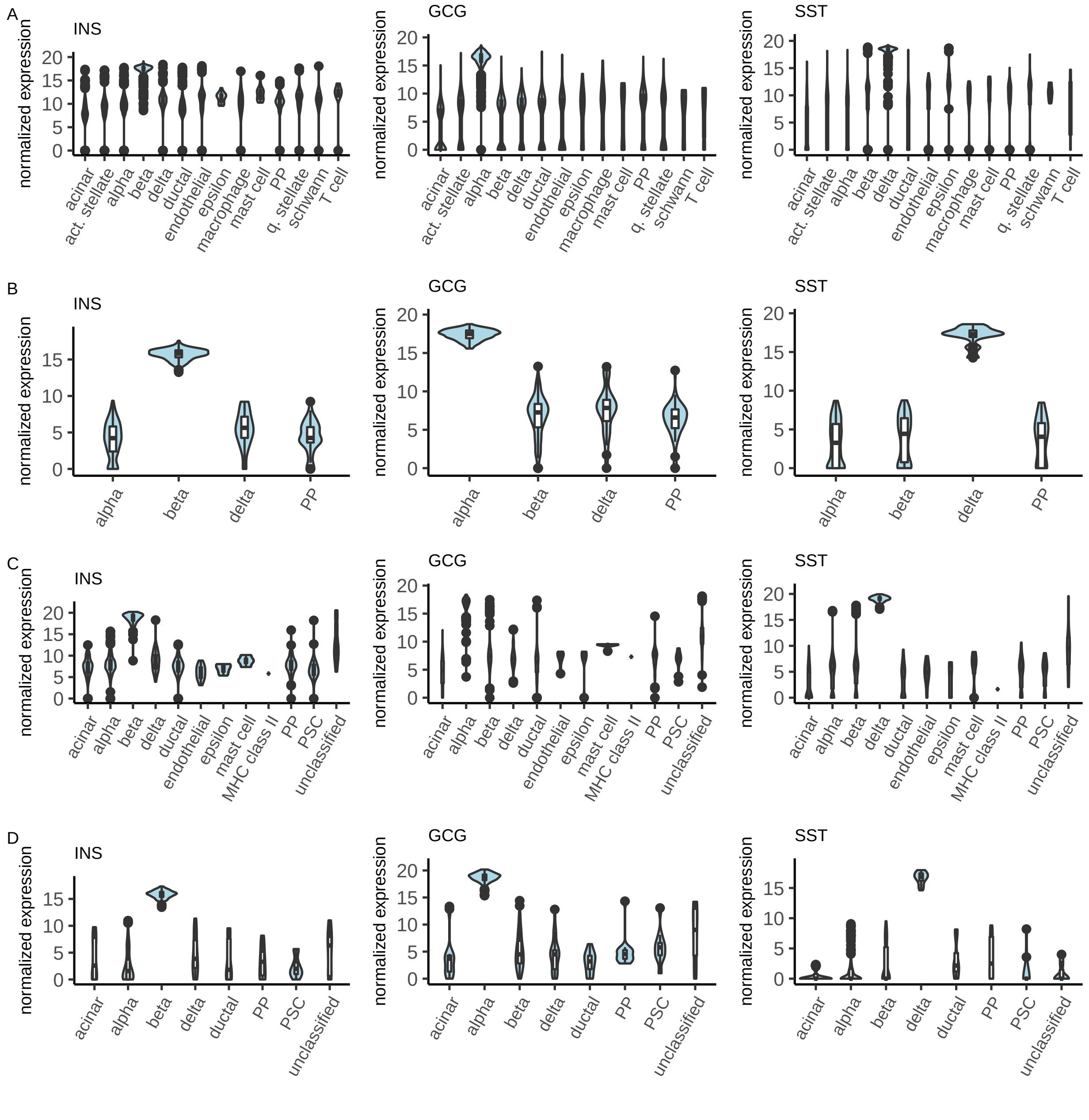

Fig. S12. EndoC-βH1 cells and adult β cells expressed α cell markers but at lower levels than α alpha cells. (A) Gene expression in adult α cells (GSE67543, N=7, FACS sorted, at least 97% purity). **(B)** Gene expression in adult β cells (GSE67543, N=7, FACS sorted, at least 97% purity). **(C)** Gene expression in EndoC-βH1 cells treated with control siRNA at 72 hours (N=3). **(D)** Protein expression in DIA proteomics on cell lysate of EndoC-βH1 cells treated with control siRNA at 72 hours (N=12). **(E)** Protein expression in TMT proteomics on cell lysate of EndoC-βH1 cells treated with control siRNA at 72 hours (N=3). No boxplot for a protein in panels C and D indicates that the protein was either not detected or quantified in $<$ 3 samples.

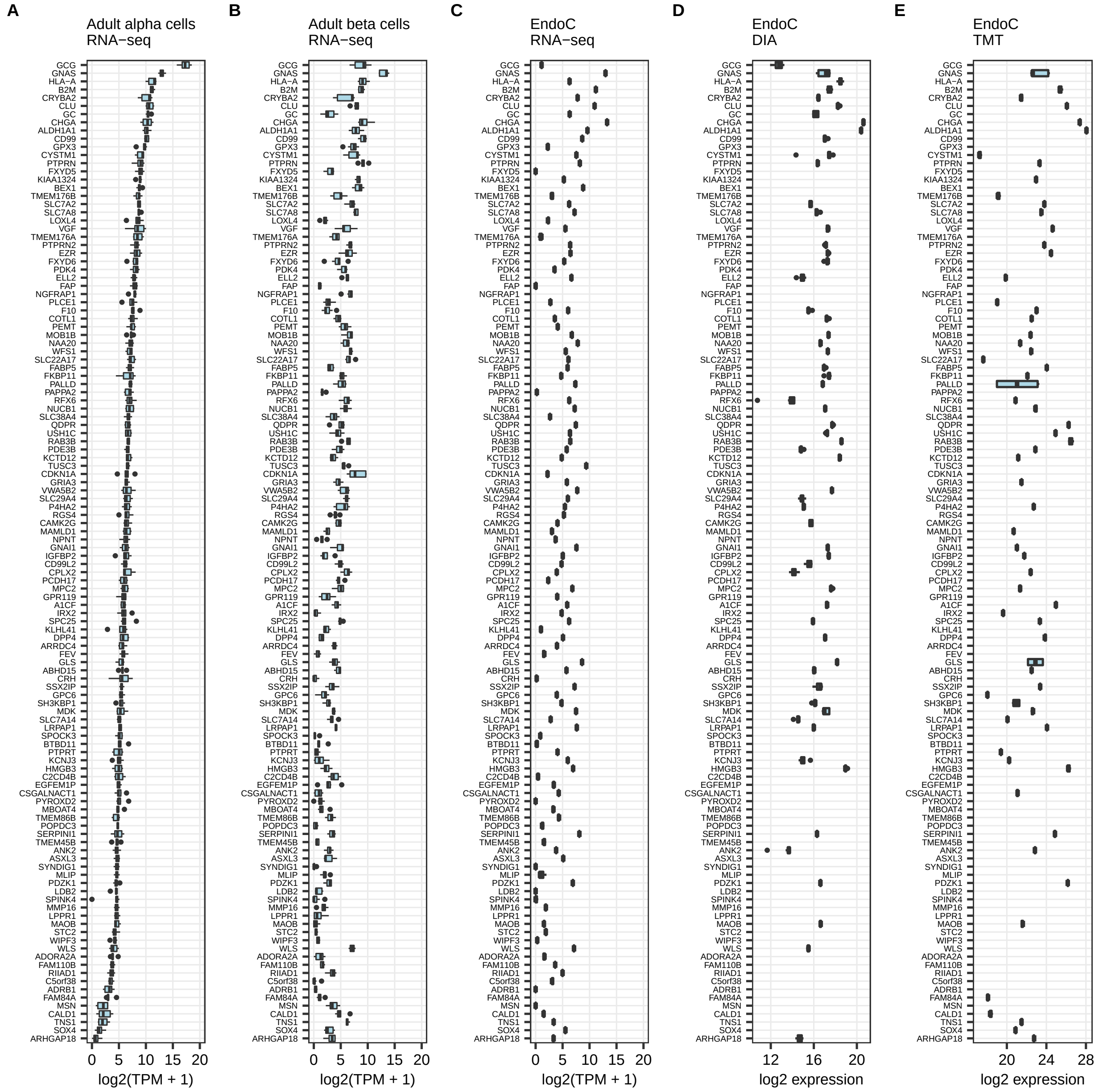

Fig. S13. EndoC-βH1 cells had reduced expression of progenitor markers. Endocrine progenitor markers were defined as markers of ECAD^{low}CD142^{neg}SUSD2^{pos} and ECAD^{low}CD142^{neg}SUSD2^{neg} in fetal pancreas at 9 weeks of gestation from Ramond et al. 2018 and median expression $<$ 1 TPM in adult β cells (GSE67543). Marker mRNA expression in **(A)** fetal pancreas 9 weeks, **(B)** adult β cells, **(C)** EndoC-βH1 cells treated with control siRNA at 72 hours (N=3). **(D)** Protein expression in DIA proteomics on cell lysate of EndoC-βH1 cells treated with control siRNA at 72 hours (N=12). **(E)** Protein expression in TMT proteomics on cell lysate of EndoC-βH1 cells treated with control siRNA at 72 hours (N=3). No boxplot for a protein in panels C and D indicates that the protein was either not detected or quantified in $<$ 3 samples.

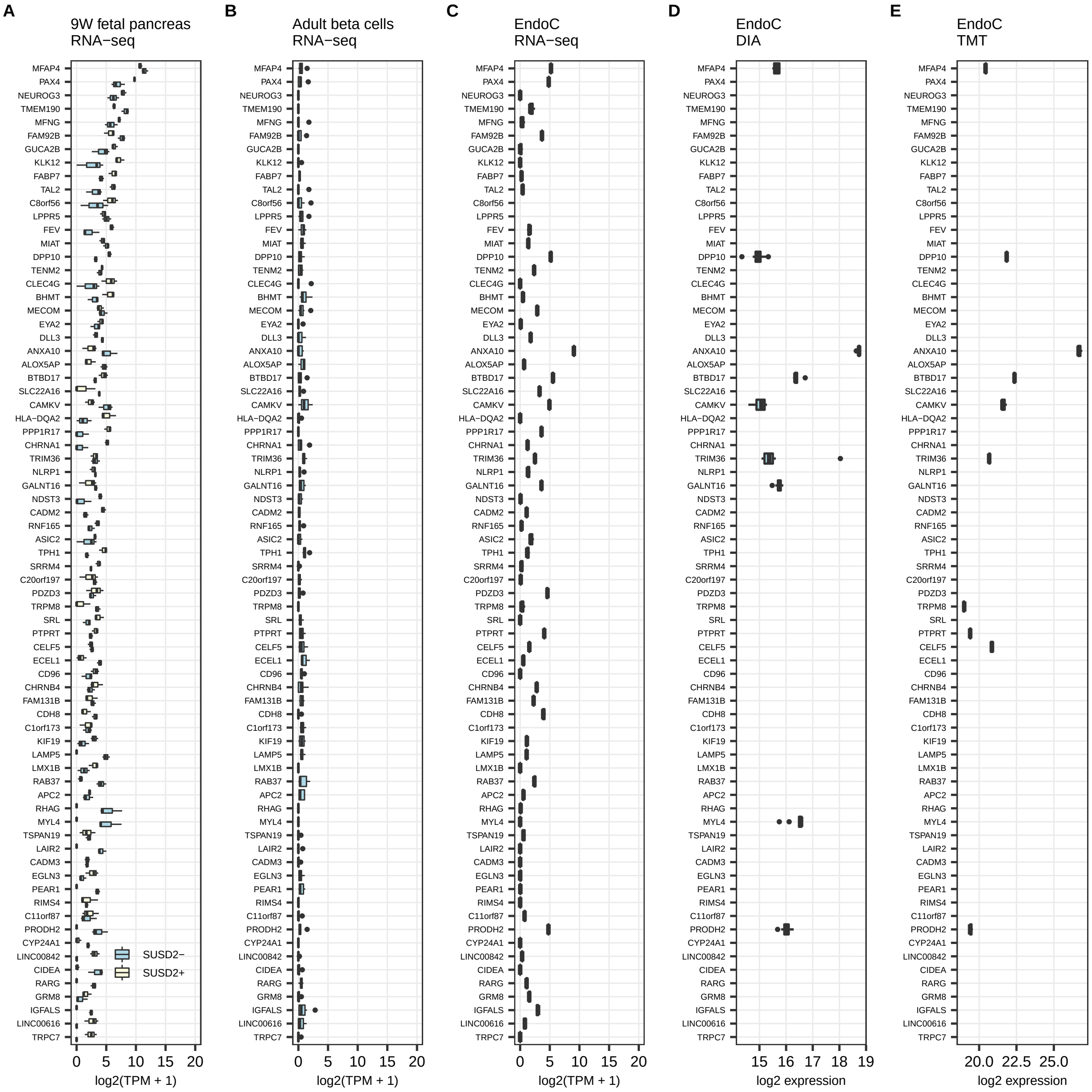

Fig. S14. EndoC-βH1 cells and adult β cells did not express fetal β cell markers. (A) Gene expression in fetal β cells (GSE67543, 12 to 18 weeks gestation, N=6). **(B)** Gene expression in adult β cells (GSE67543, N=7, FACS sorted, at least 97% purity). **(C)** Gene expression in EndoC-βH1 cells treated with control siRNA at 72 hours (N=3). **(D)** Protein expression in DIA proteomics on cell lysate of EndoC-βH1 cells treated with control siRNA at 72 hours (N=12). **(E)** Protein expression in TMT proteomics on cell lysate of EndoC-βH1 cells treated with control siRNA at 72 hours (N=3). No boxplot for a protein in panels C and D indicates that the protein was either not detected or quantified in $<$ 3 samples.

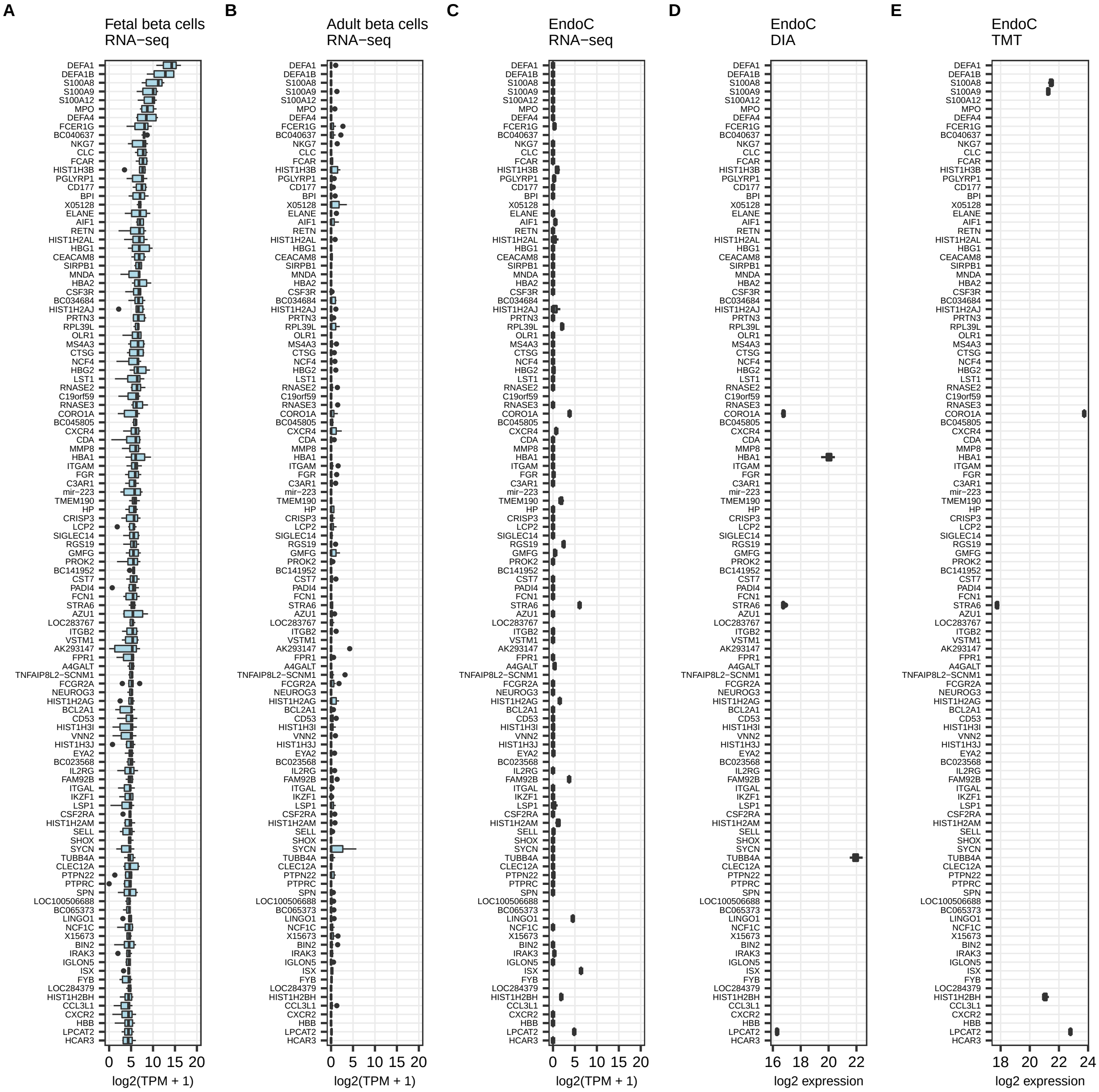

Fig. S15. EndoC-βH1 cells did not express acinar cell markers. (A) Gene expression in adult acinar cells (GSE57973, N= 2). **(B)** Gene expression in adult β cells (GSE57973, N=5). **(C)** Gene expression in EndoC-βH1 cells treated with control siRNA at 72 hours (N=3). **(D)** Protein expression in DIA proteomics on cell lysate of EndoC-βH1 cells treated with control siRNA at 72 hours (N=12). **(E)** Protein expression in TMT proteomics on cell lysate of EndoC-βH1 cells treated with control siRNA at 72 hours (N=3). No boxplot for a protein in panels C and D indicates that the protein was either not detected or quantified in $<$ 3 samples.

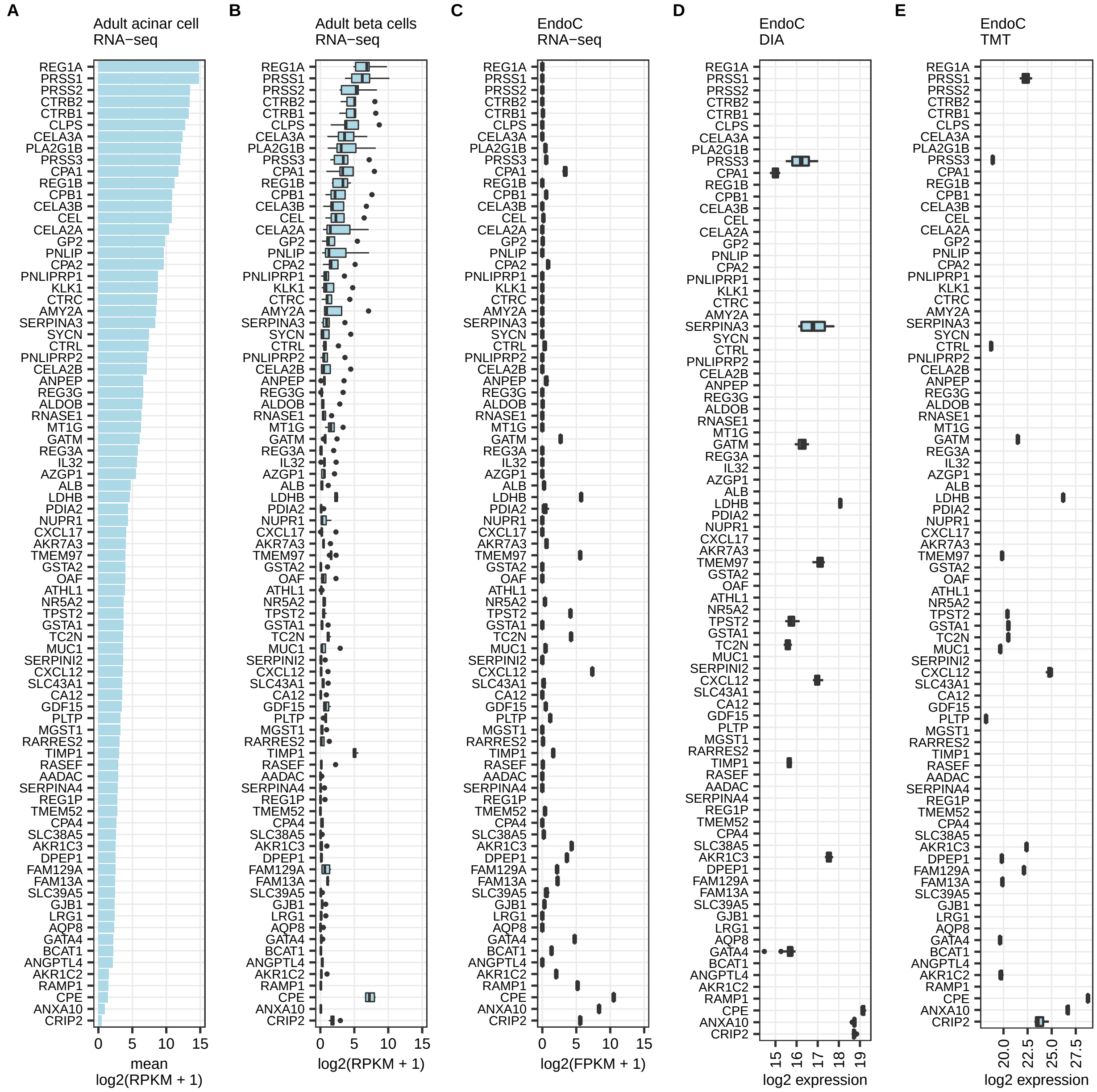

Fig. S16. EndoC-βH1 cells did not express ductal cell markers. (A) Gene expression in adult ductal cells (GSE57973, N= 2). **(B)** Gene expression in adult β cells (GSE57973, N=5). **(C)** Gene expression in EndoC-βH1 cells treated with control siRNA at 72 hours (N=3). **(D)** Protein expression in DIA proteomics on cell lysate of EndoC-βH1 cells treated with control siRNA at 72 hours (N=12). **(E)** Protein expression in TMT proteomics on cell lysate of EndoC-βH1 cells treated with control siRNA at 72 hours (N=3). No boxplot for a protein in panels C and D indicates that the protein was either not detected or quantified in $<$ 3 samples.

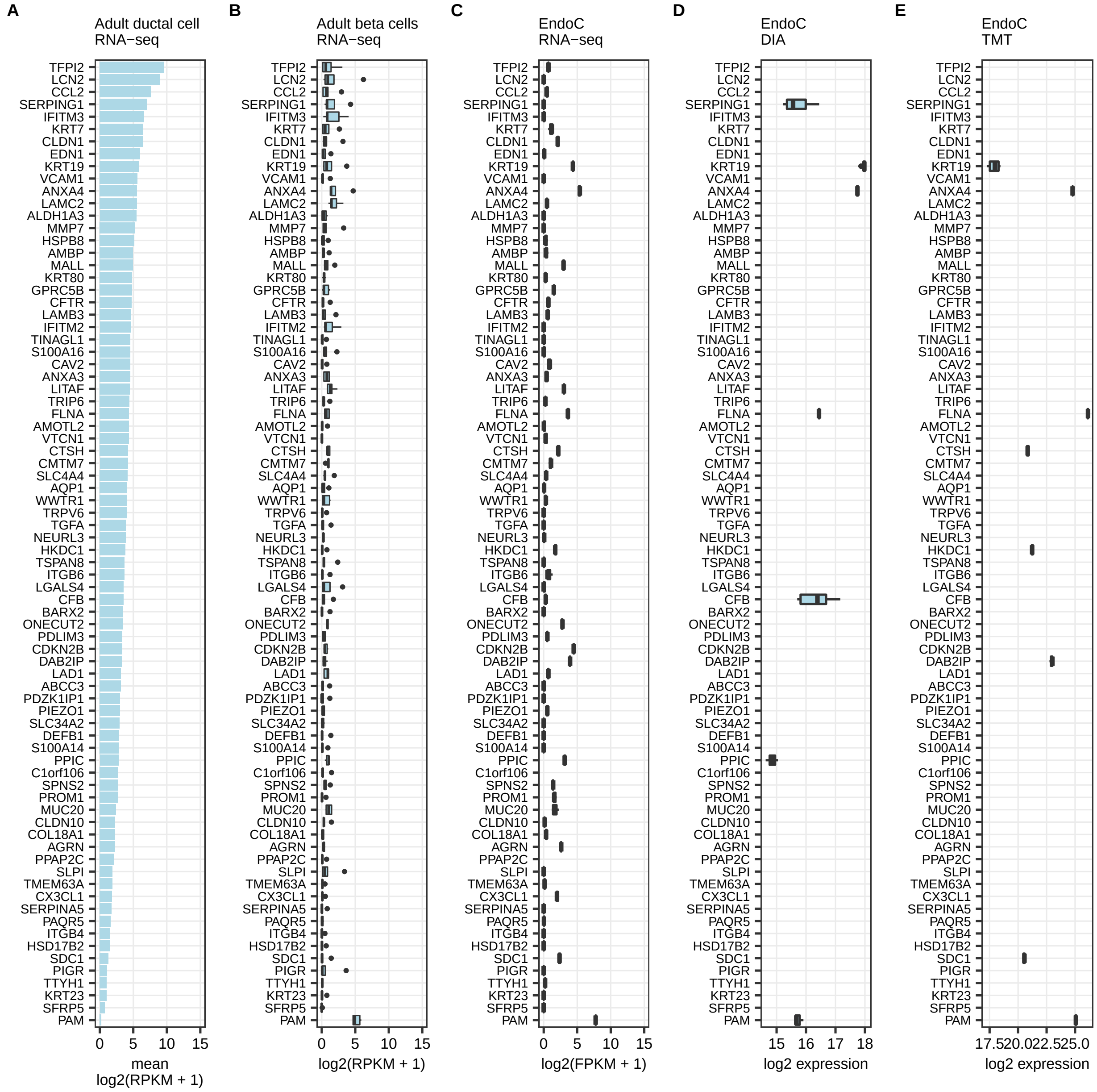

Fig. S17. Ectopic expression of extra-islet tissue markers was not observed in EndoC-βH1 cells. Tissue-specific genes (x=10) were extracted from supplementary data of Ryaboshapkina et al. 2019. Pancreas-specific genes were excluded from the analysis. **(A)** Median gene expression in the corresponding tissue with the highest expression in GTEx. N markers = 509. **(B)** Gene expression in adult β cells (GSE67543, N=7, FACS sorted, at least 97% purity). **(C)** Gene expression in EndoC-βH1 cells treated with control siRNA at 72 hours (N=3). **(D)** Protein expression in DIA proteomics on cell lysate of EndoC-βH1 cells treated with control siRNA at 72 hours (N=12). **(E)** Protein expression in TMT proteomics on cell lysate of EndoC-βH1 cells treated with control siRNA at 72 hours (N=3). No boxplot for a protein in panels C and D indicates that the protein was either not detected or quantified in $<$ 3 samples.

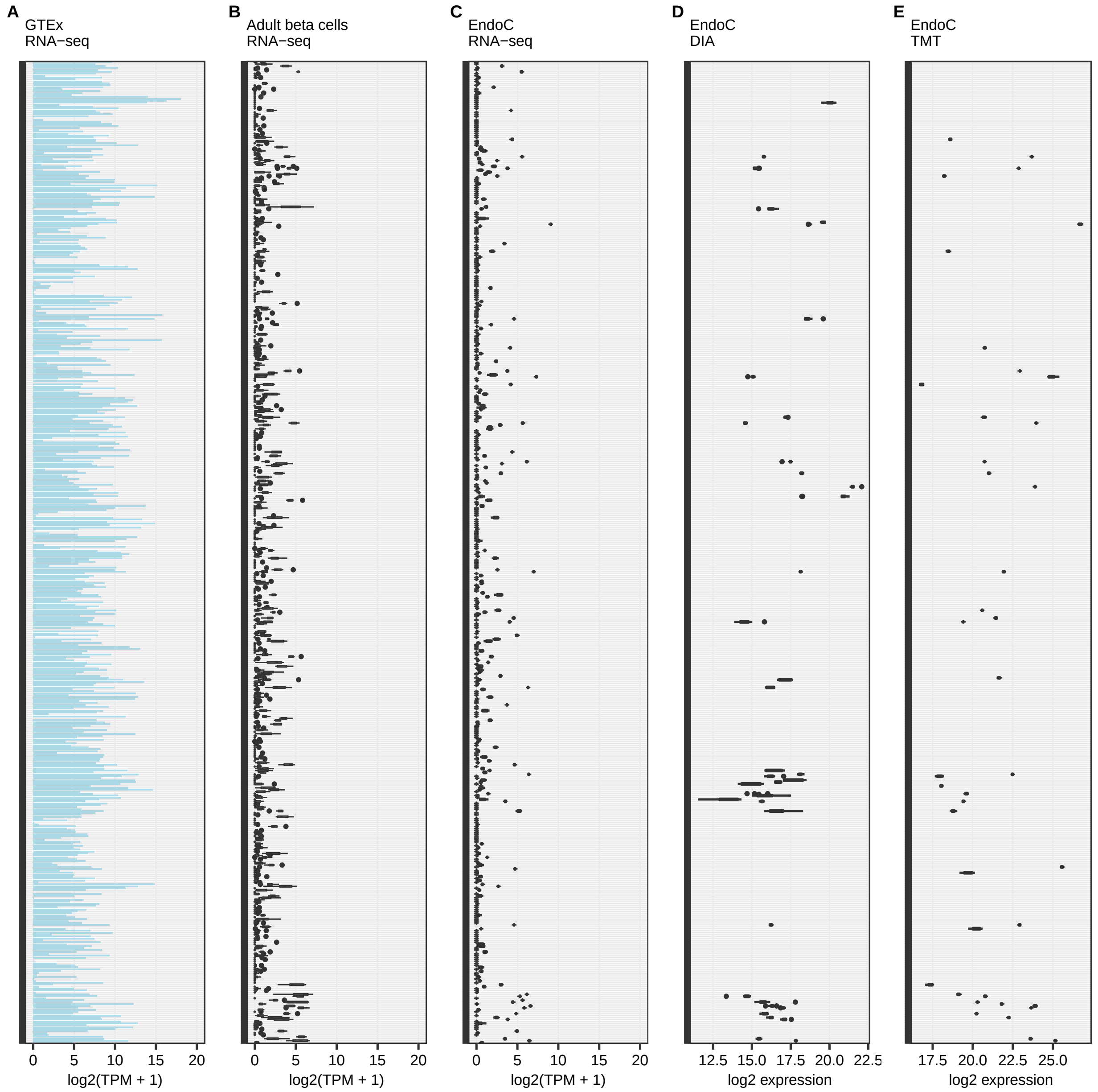

Fig S18. Potential chromogranin A bioactive peptides in secretome of EndoC-βH1 cells. CLUSTAL O(1.2.4) multiple sequence alignment of bioactive peptide sequences (yellow) vs peptide sequences detected in the DIA secretome experiment at 72h. Alignment was performed online with UniProt (https://www.uniprot.org/align/).

(A) EA-92

```
Reference EDSKEAEKSGEATDGARPQALPEPMQESKAEGNNQAPGEEEEEEEEEATNTHPPASLPSQKYPGPQAEGDSEGLSQGLVDREKGLSAEPGWQA
Peptide 1 -------------------------------------------------------------YPGPQAEGDSEGLSQGLVDREK----------
Peptide 2 -------------------------------------------------------------YPGPQAEGDSEGLSQGLVDR------------
Peptide 3 -----------------------------AEGNNQAPGEEEEEEEEEATNTHPPASLPSQK--------------------------------
Peptide 4 --------SGEATDGARPQALPEPMQESK----------------------------------------------------------------
Peptide 5 -----------------PQALPEPMQESK----------------------------------------------------------------
Peptide 6 ----EAEKSGEATDGARPQALPEPMQESK----------------------------------------------------------------
```

(B) Pancreastatin

```
Reference SEALAVDGAGKPGAEEAQDPEGKGEQEHSQQKEEEEEMAVVPQGLFRG
Peptide 1 SEALAVDGAGK-------------------------------------
Peptide 2 SEALAVDGAGKPGAEEAQDPEGK-------------------------
Peptide 3 --------------------------------EEEEEMAVVPQGLFR-
Peptide 4 -----------PGAEEAQDPEGK-------------------------
Peptide 5 SEALAVDGAGKPGAEEAQDPEGKGEQEHSQQK----------------
Peptide 6 -----------------------GEQEHSQQKEEEEEMAVVPQGLFR-
Peptide 7 -----------PGAEEAQDPEGKGEQEHSQQK----------------
Peptide 8 -----------------------GEQEHSQQK----------------
```

(C) SS-18

```
Reference SGELEQEEERLSKEWEDS
Peptide 1 SGELEQEEER--------
Peptide 2 SGELEQEEERLSK-----
```

(D) LF-19

```
Reference LEGQEEEEDNRDSSMKLSF
Peptide 1 LEGQEEEEDNR--------
Peptide 2 LEGQEEEEDNRDSSMK---
```

(E) GE-25

```
Reference GWRPSSREDSLEAGLPLQVRGYPEE
Peptide 1 -------EDSLEAGLPLQVR-----
Peptide 2 ---PSSREDSLEAGLPLQVR-----
```

(E) Vasostatins (vasostatin-2, potentially vasostatin-1)

```
Vasostatin-2 LPVNSPMNKGDTEVMKCIVEVISDTLSKPSPMPVSQECFETLRGDERILSILRHQNLLKELQDLALQGAKERAHQQKKHSGFEDELSEVLENQSSQAELKEAVEEPSSKDVME
Vasostatin-1 LPVNSPMNKGDTEVMKCIVEVISDTLSKPSPMPVSQECFETLRGDERILSILRHQNLLKELQDLALQGAKERAHQQ-------------------------------------
Peptide 1    ------------------------------------------------------------------------------HSGFEDELSEVLENQSSQAELK-------------
Peptide 2    -----------------------------------------------------------ELQDLALQGAK-------------------------------------------
Peptide 3    ----------------------------------------------------------------------------------------------------EAVEEPSSK----
Peptide 4    ------------------------------------------------------------------------------HSGFEDELSEVLENQSSQAELKEAVEEPSSK----
Peptide 5    -----------------------------------------------------------------------------KHSGFEDELSEVLENQSSQAELK-------------
Peptide 6    -----------------------------------------------------------ELQDLALQGAKER-----------------------------------------
Peptide 7    ---------GDTEVMK-------------------------------------------------------------------------------------------------
Peptide 8    -----------------------------------------------------HQNLLKELQDLALQGAK-------------------------------------------
```

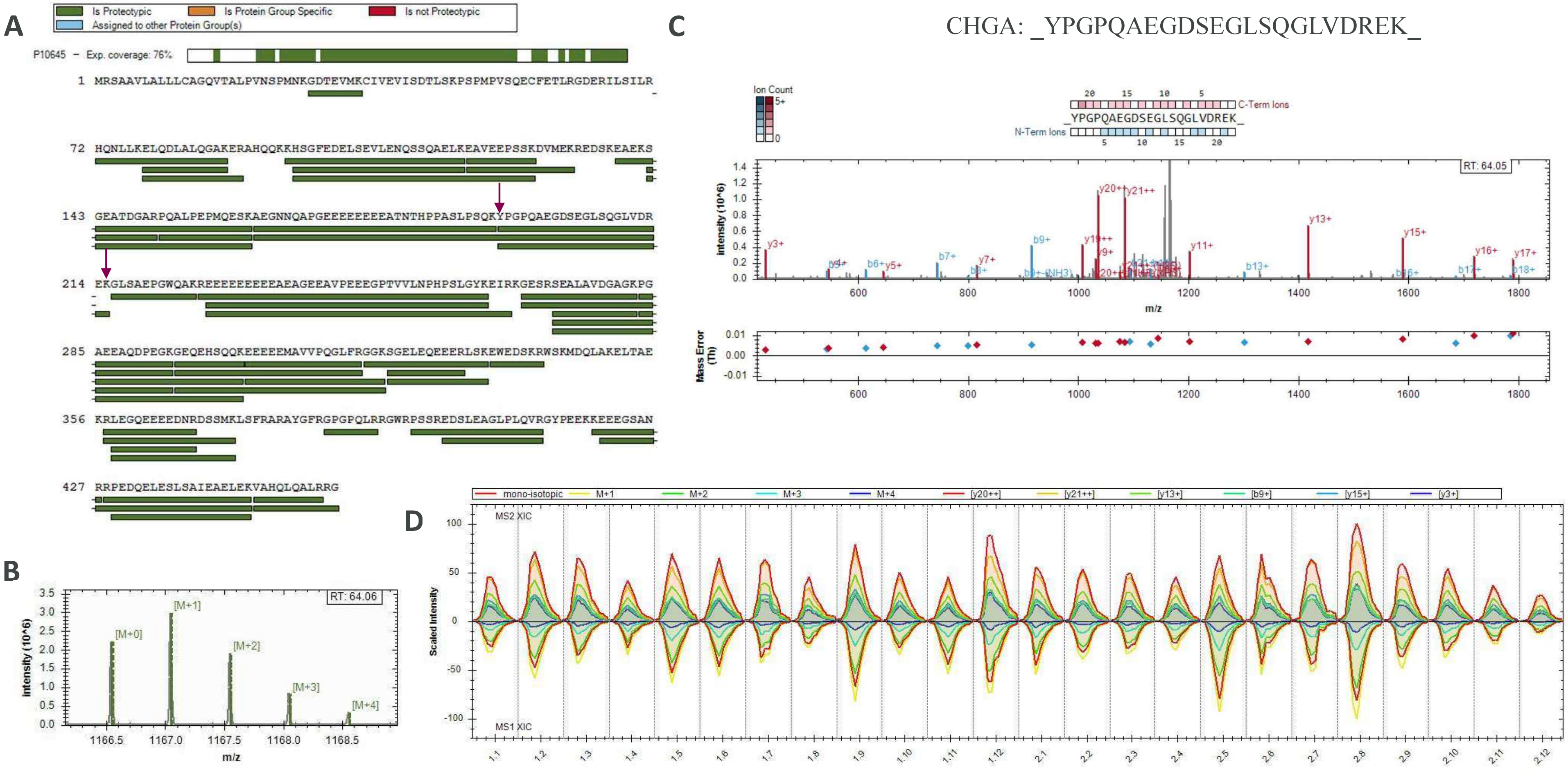

Fig. S19. Chromogranin A proteotypic peptides identified by DIA secretome proteomics experiment at 72h. Total protein coverage (**A**), MS1 isotopic envelope (**B**), MS/MS fragmentation match and mass error (**C**), and XIC of MS1 & MS2 for the conditions (**D**) are shown for each given peptide identified.

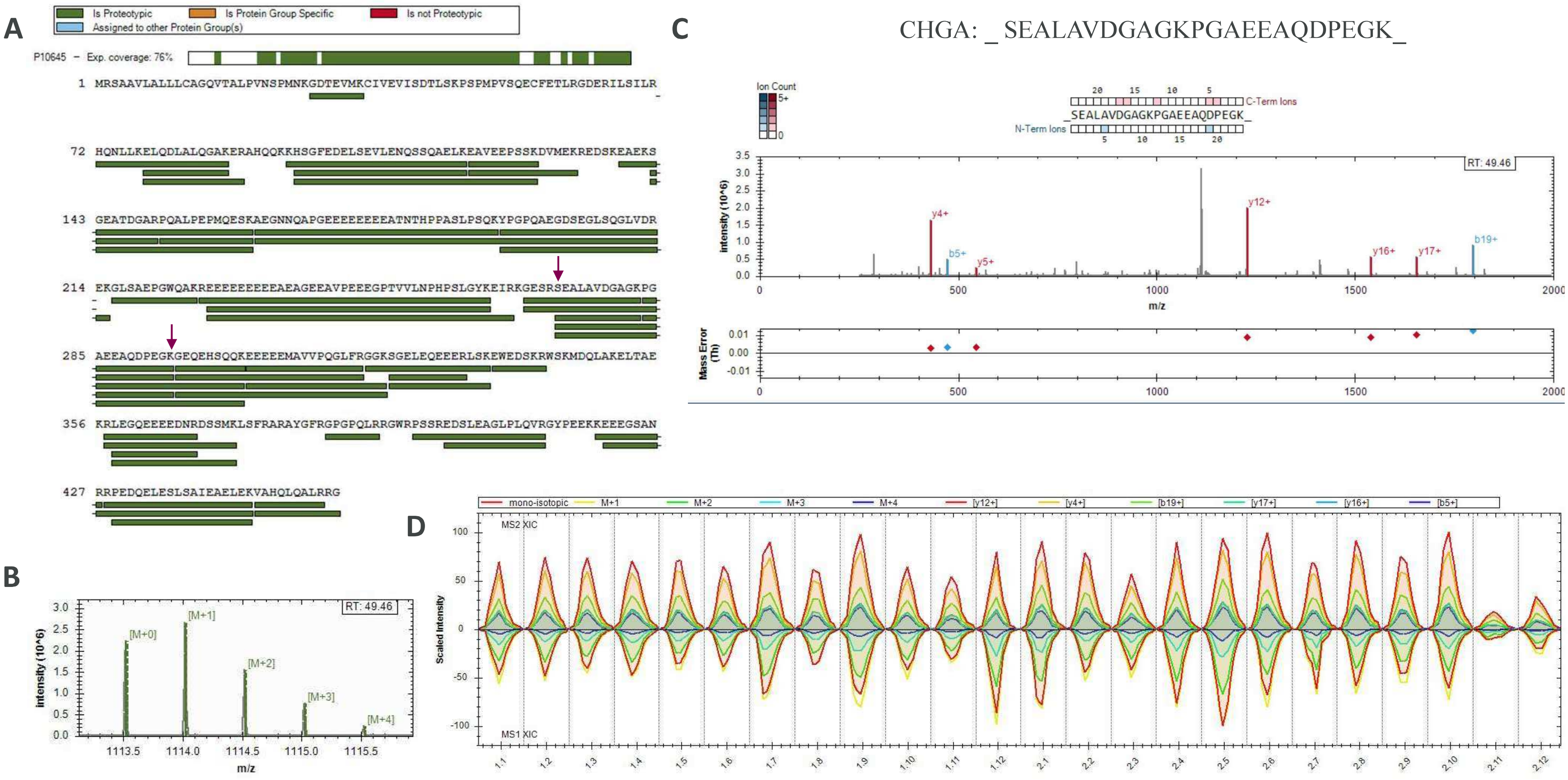

Fig. S20. Chromogranin A proteotypic peptides identified by DIA secretome proteomics experiment at 72h. Total protein coverage (**A**), MS1 isotopic envelope (**B**), MS/MS fragmentation match and mass error (**C**), and XIC of MS1 & MS2 for the conditions (**D**) are shown for each given peptide identified.

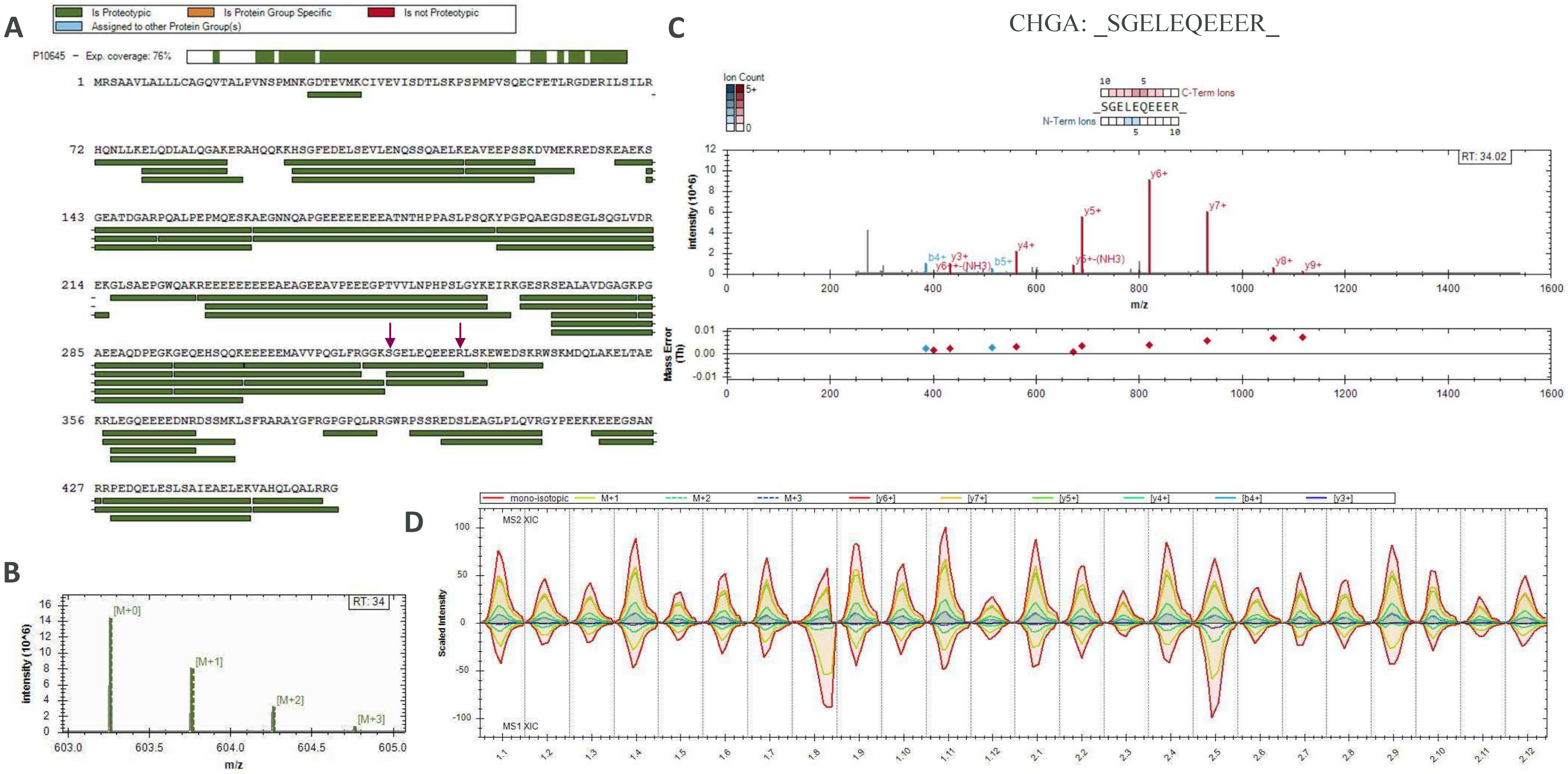

Fig. S21. Chromogranin A proteotypic peptides identified by DIA secretome proteomics experiment at 72h. Total protein coverage (**A**), MS1 isotopic envelope (**B**), MS/MS fragmentation match and mass error (**C**), and XIC of MS1 & MS2 for the conditions (**D**) are shown for each given peptide identified.

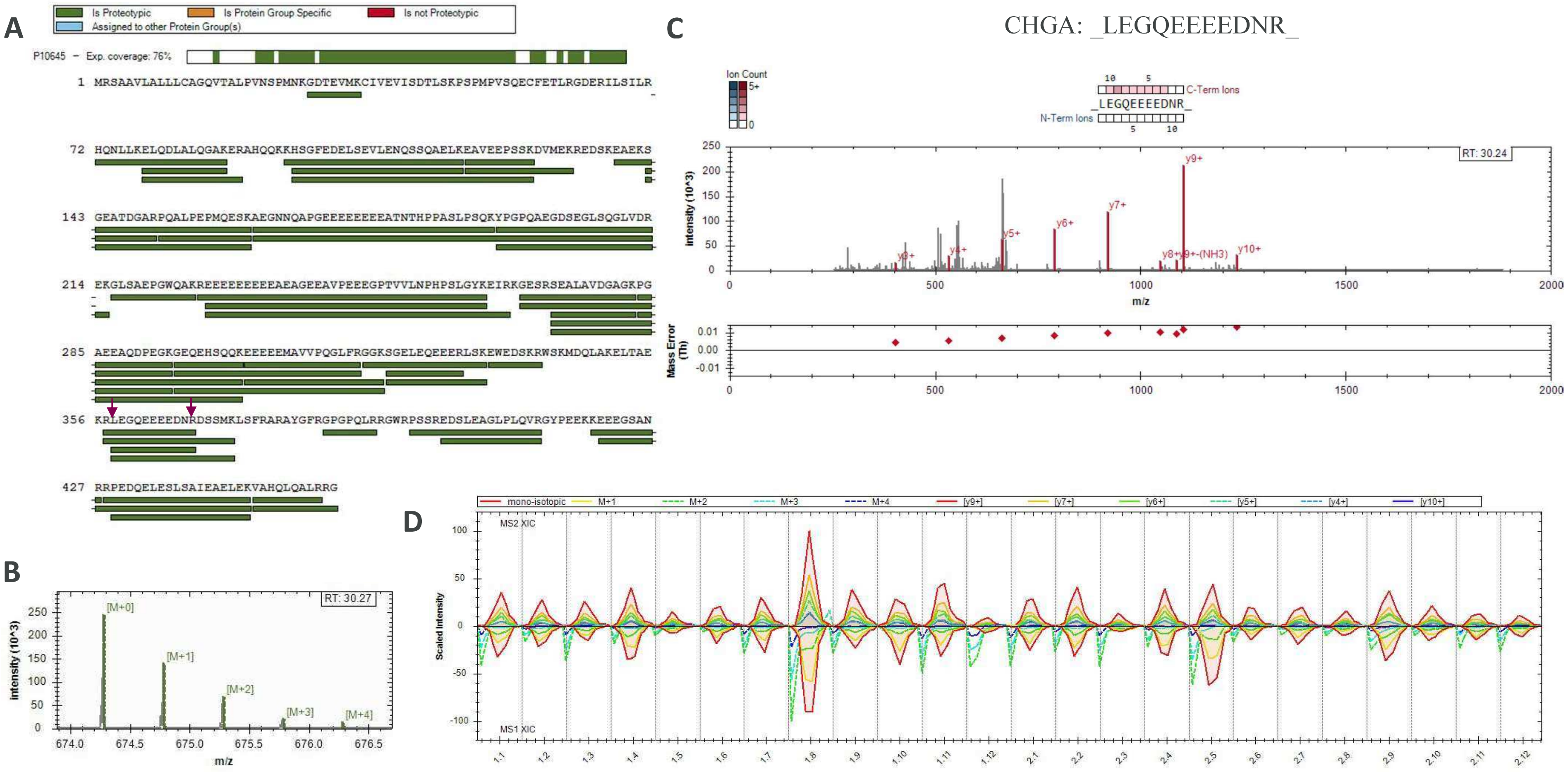

Fig. S22. Chromogranin A proteotypic peptides identified by DIA secretome proteomics experiment at 72h. Total protein coverage (**A**), MS1 isotopic envelope (**B**), MS/MS fragmentation match and mass error (**C**), and XIC of MS1 & MS2 for the conditions (**D**) are shown for each given peptide identified.

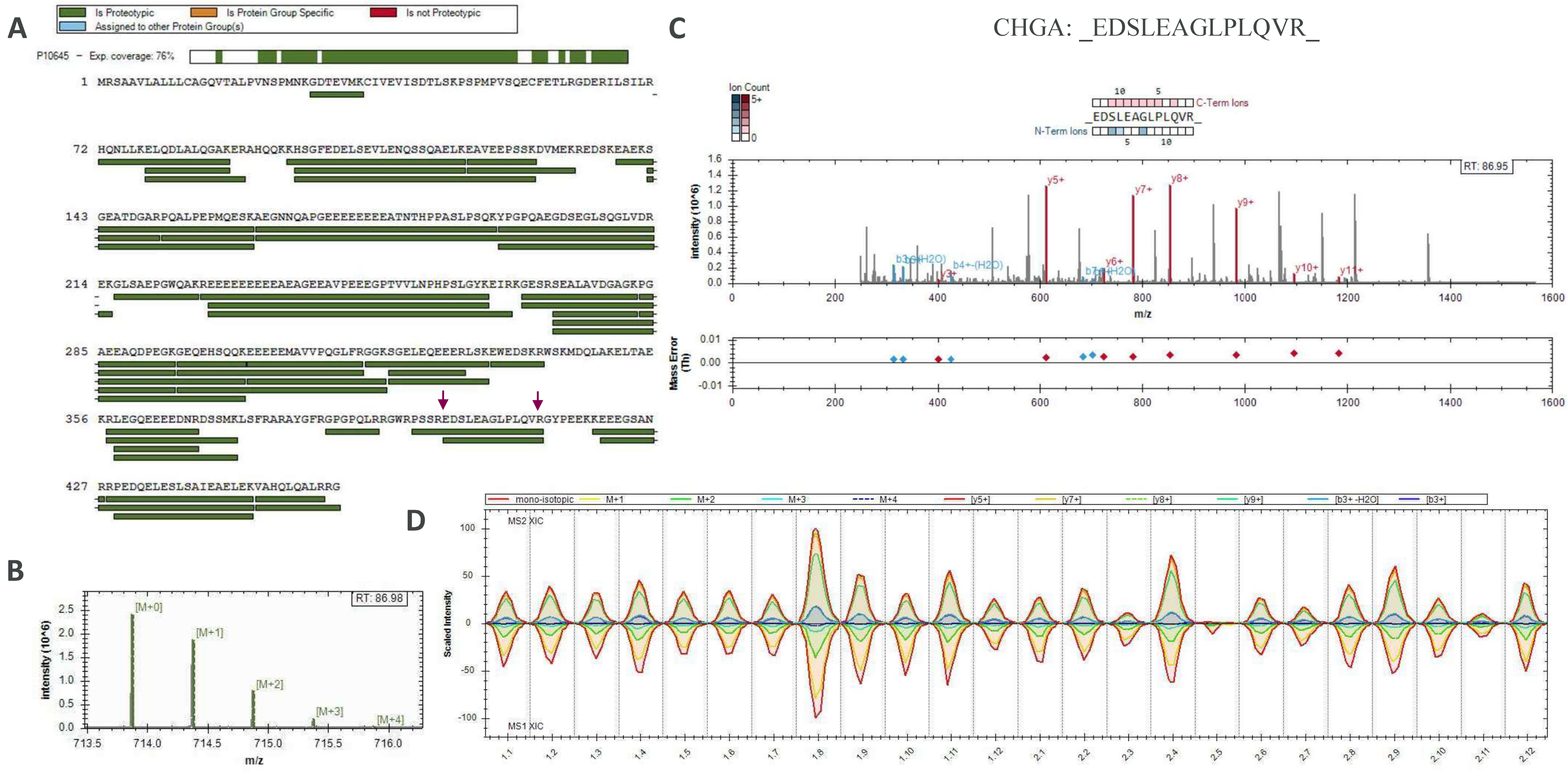

Fig. S23. Chromogranin A proteotypic peptides identified by DIA secretome proteomics experiment at 72h. Total protein coverage (**A**), MS1 isotopic envelope (**B**), MS/MS fragmentation match and mass error (**C**), and XIC of MS1 & MS2 for the conditions (**D**) are shown for each given peptide identified.

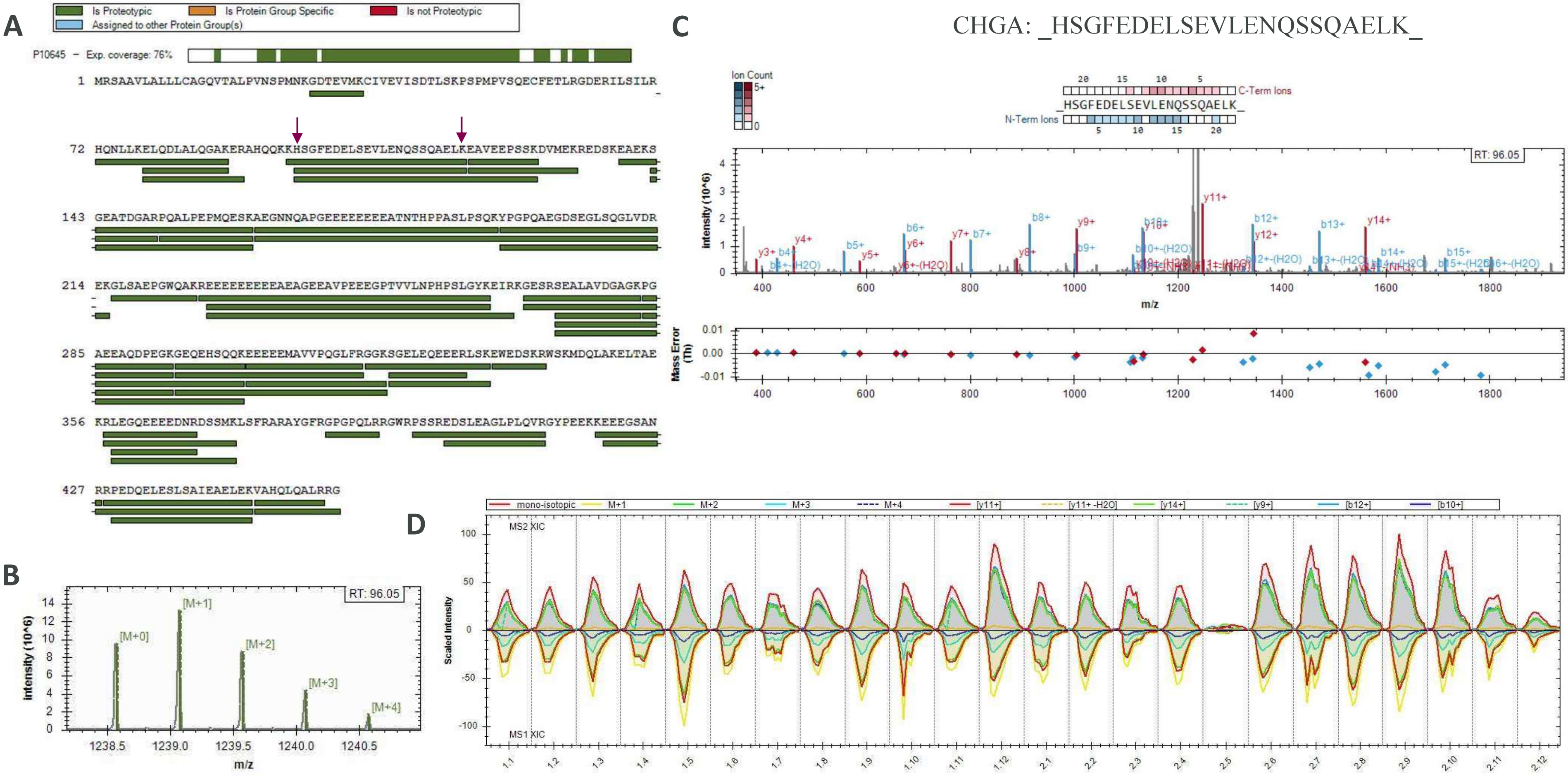

Fig. S24. Chromogranin A proteotypic peptides identified by DIA secretome proteomics experiment at 72h. Total protein coverage (**A**), MS1 isotopic envelope (**B**), MS/MS fragmentation match and mass error (**C**), and XIC of MS1 & MS2 for the conditions (**D**) are shown for each given peptide identified.

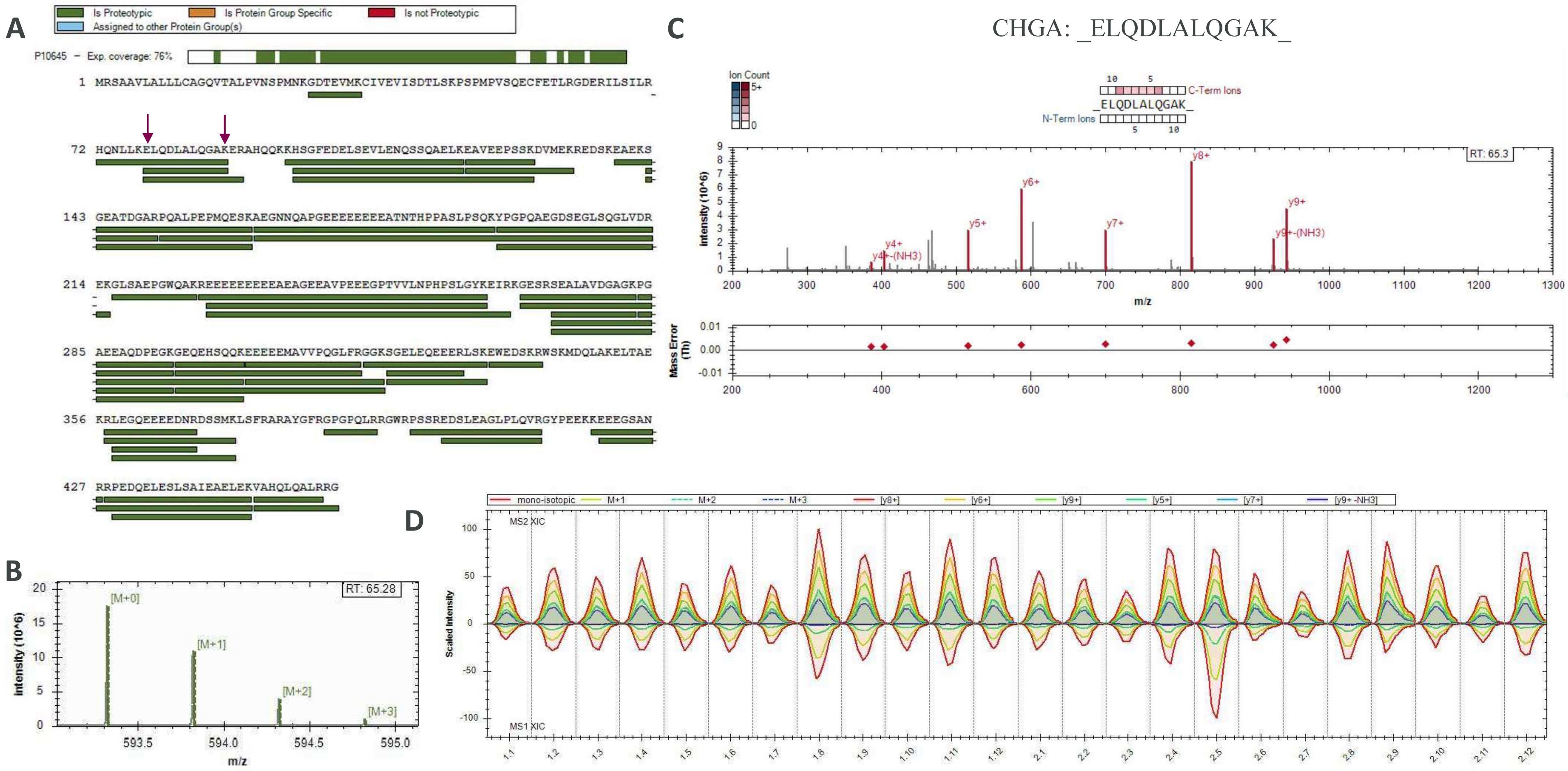

Fig. S25. Chromogranin A proteotypic peptides identified by DIA secretome proteomics experiment at 72h. Total protein coverage (**A**), MS1 isotopic envelope (**B**), MS/MS fragmentation match and mass error (**C**), and XIC of MS1 & MS2 for the conditions (**D**) are shown for each given peptide identified.

Fig S26. Potential chromogranin B bioactive peptides in secretome of EndoC-βH1 cells. CLUSTAL O(1.2.4) multiple sequence alignment of bioactive peptide sequences (yellow) vs peptide sequences detected in the DIA secretome experiment at 72h. Alignment was performed online with UniProt (https://www.uniprot.org/align/).

(A) CCB peptide

```
Reference SAEFPDFYDSEEPVSTHQEAENEKDRADQTVLTEDEKKELENLAAMDLELQKIAEKF
Peptide 1 ------------------------DRADQTVLTEDEK--------------------
Peptide 2 --------------------------ADQTVLTEDEK--------------------
Peptide 3 --------------------------------------ELENLAAMDLELQK-----
Peptide 4 ------------------------DRADQTVLTEDEKK-------------------
```

(B) GAWK peptide

```
Reference FLGEGHHRVQENQMDKARRHPQGAWKELDRNYLNYGEEGAPGKWQQQGDLQDTKENREEARFQDKQYSSHHTAE
Peptide 1 -------------------------------------------WQQQGDLQDTK--------------------
Peptide 2 ------------------------------NYLNYGEEGAPGK-------------------------------
Peptide 3 --------------------------ELDRNYLNYGEEGAPGK-------------------------------
Peptide 4 FLGEGHHR------------------------------------------------------------------
```

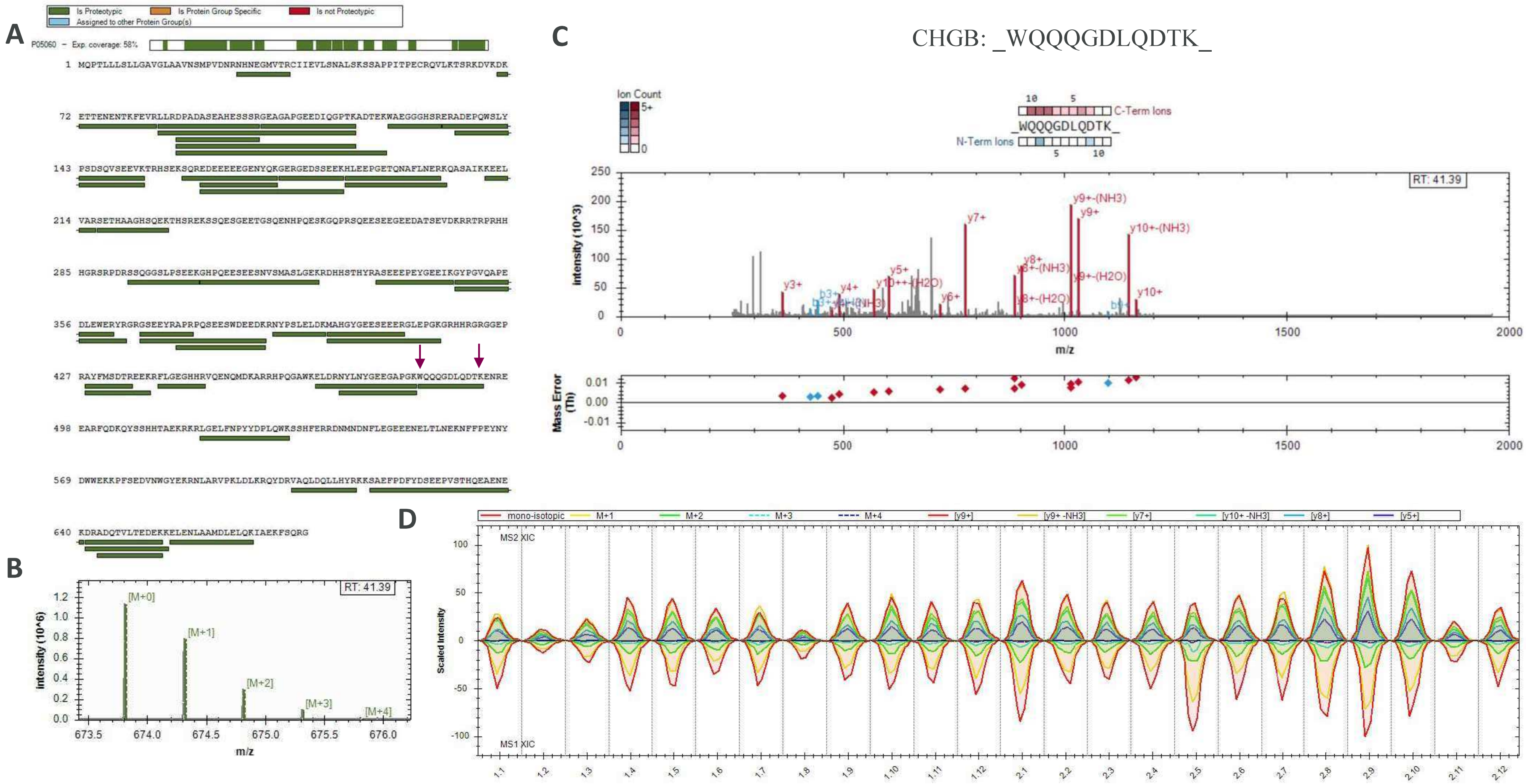

Fig. S27. Chromogranin B proteotypic peptides identified in PXD027913. Total protein coverage (**A**), MS1 isotopic envelope (**B**), MS/MS fragmentation match and mass error (**C**), and XIC of MS1 & MS2 for the conditions (**D**) are shown for each given peptide identified.

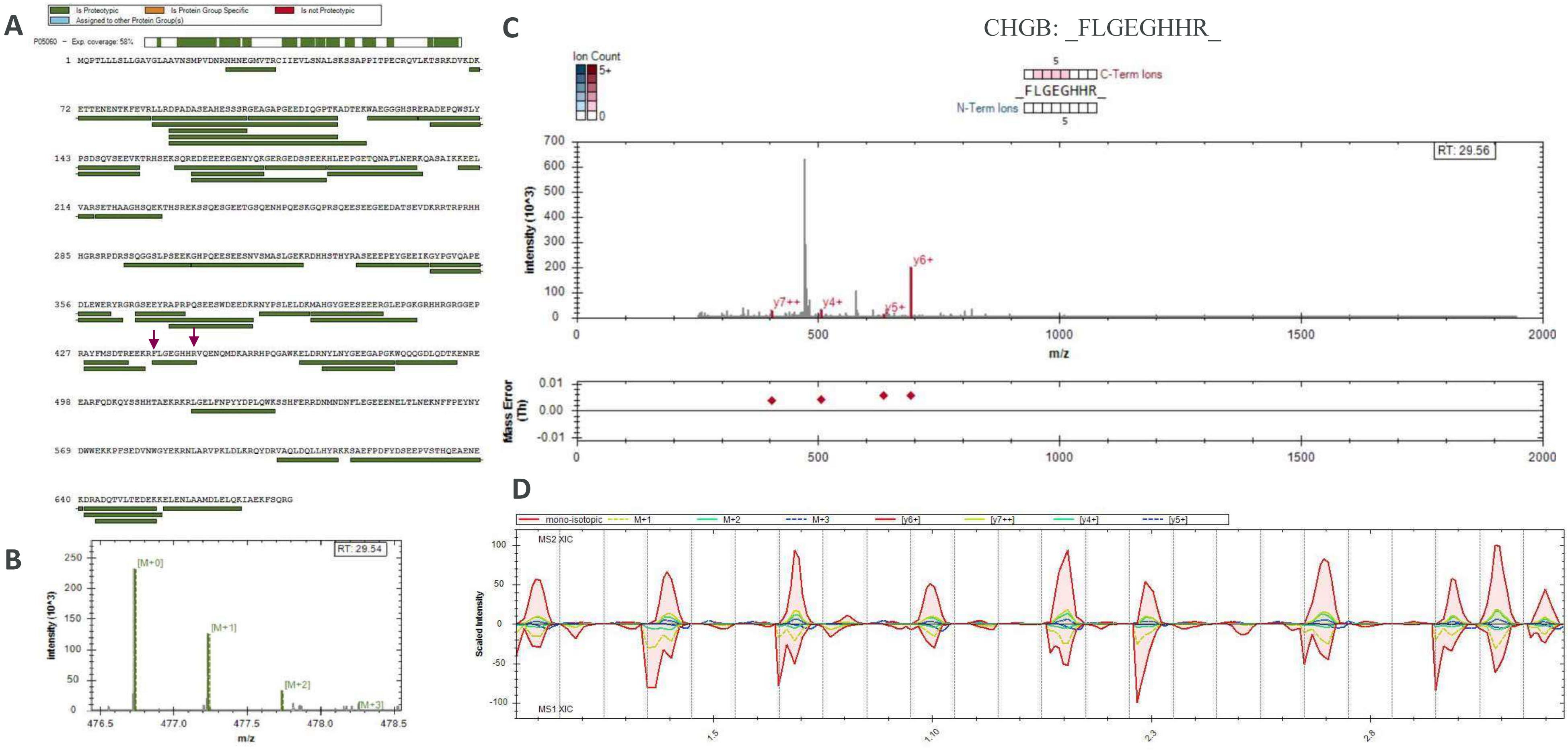

Fig. S28. Chromogranin B proteotypic peptides identified in PXD027913. Total protein coverage (**A**), MS1 isotopic envelope (**B**), MS/MS fragmentation match and mass error (**C**), and XIC of MS1 & MS2 for the conditions (**D**) are shown for each given peptide identified.

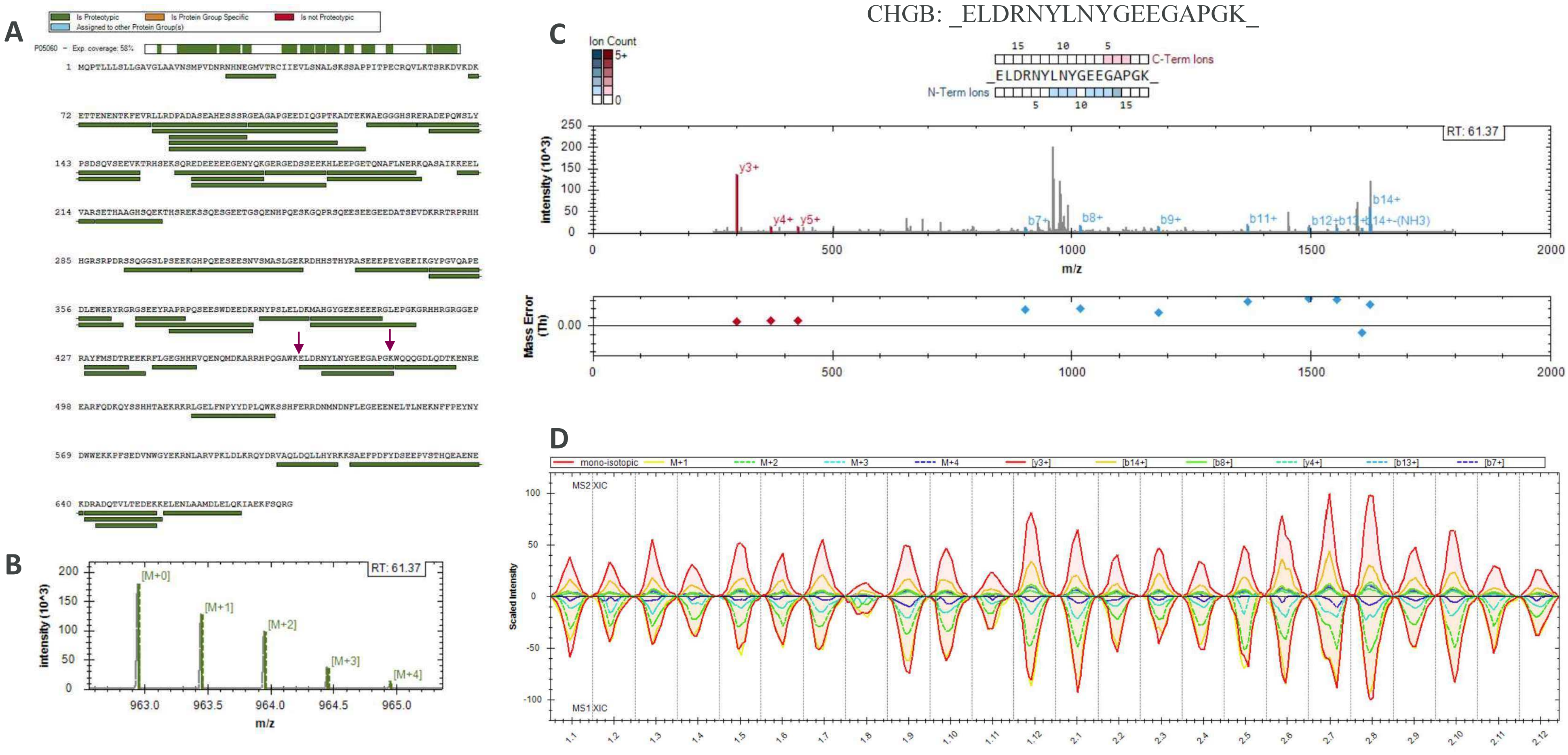

Fig. S29. Chromogranin B proteotypic peptides identified in PXD027913. Total protein coverage (**A**), MS1 isotopic envelope (**B**), MS/MS fragmentation match and mass error (**C**), and XIC of MS1 & MS2 for the conditions (**D**) are shown for each given peptide identified.

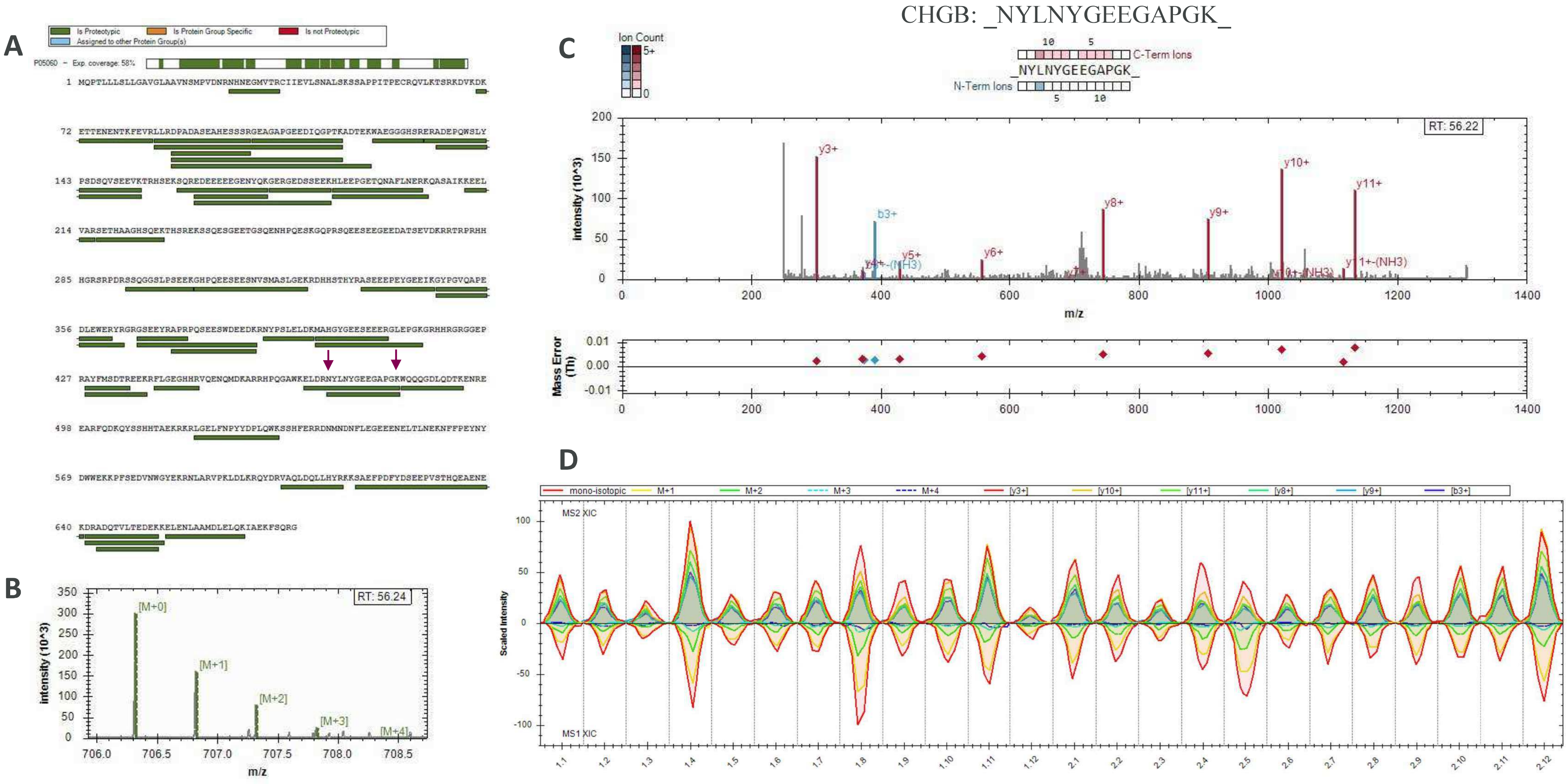

Fig. S30. Chromogranin B proteotypic peptides identified in PXD027913. Total protein coverage (**A**), MS1 isotopic envelope (**B**), MS/MS fragmentation match and mass error (**C**), and XIC of MS1 & MS2 for the conditions (**D**) are shown for each given peptide identified.

CHGB: _ELENLAAMDLELQK_

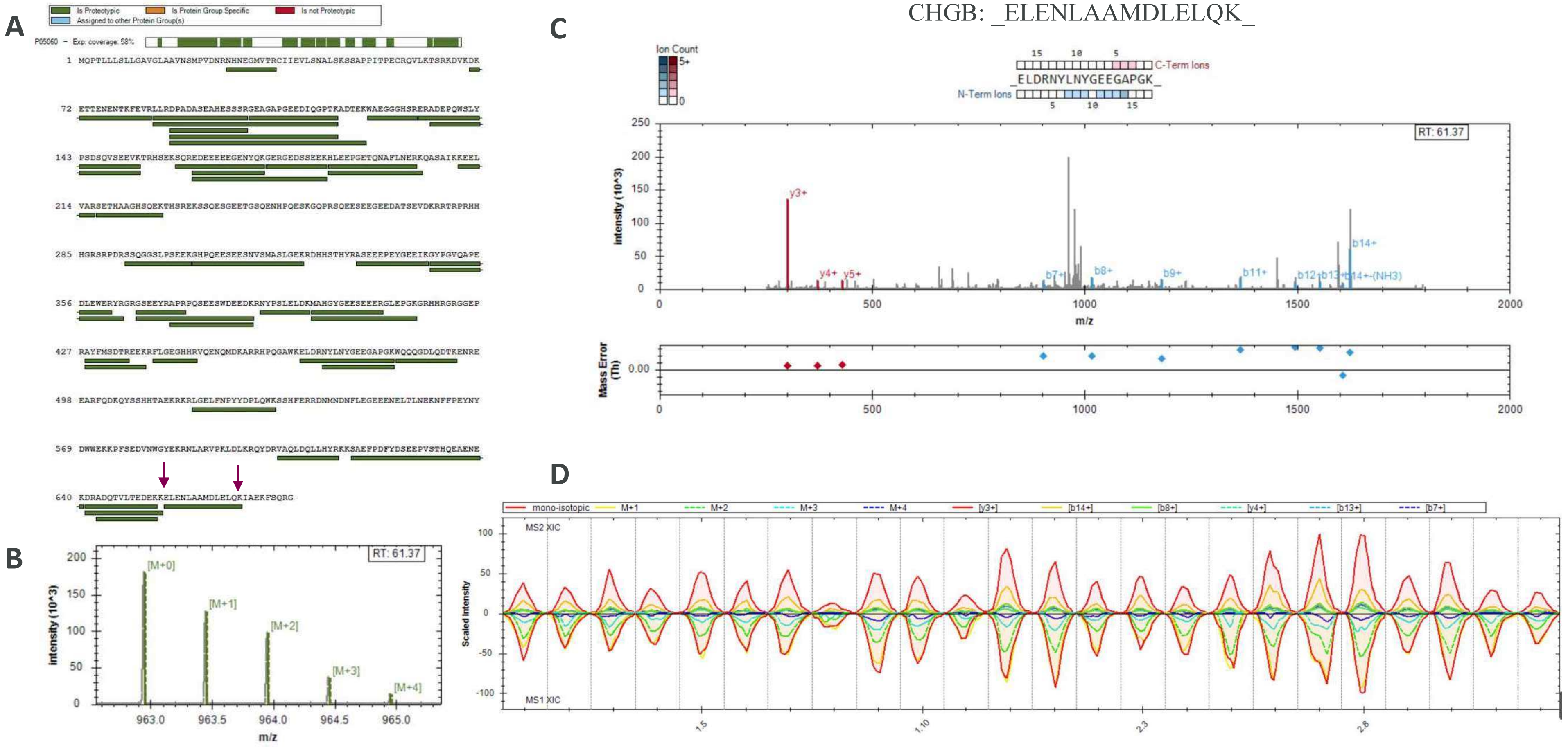

Fig. S31. Chromogranin B proteotypic peptides identified in PXD027913. Total protein coverage (**A**), MS1 isotopic envelope (**B**), MS/MS fragmentation match and mass error (**C**), and XIC of MS1 & MS2 for the conditions (**D**) are shown for each given peptide identified.

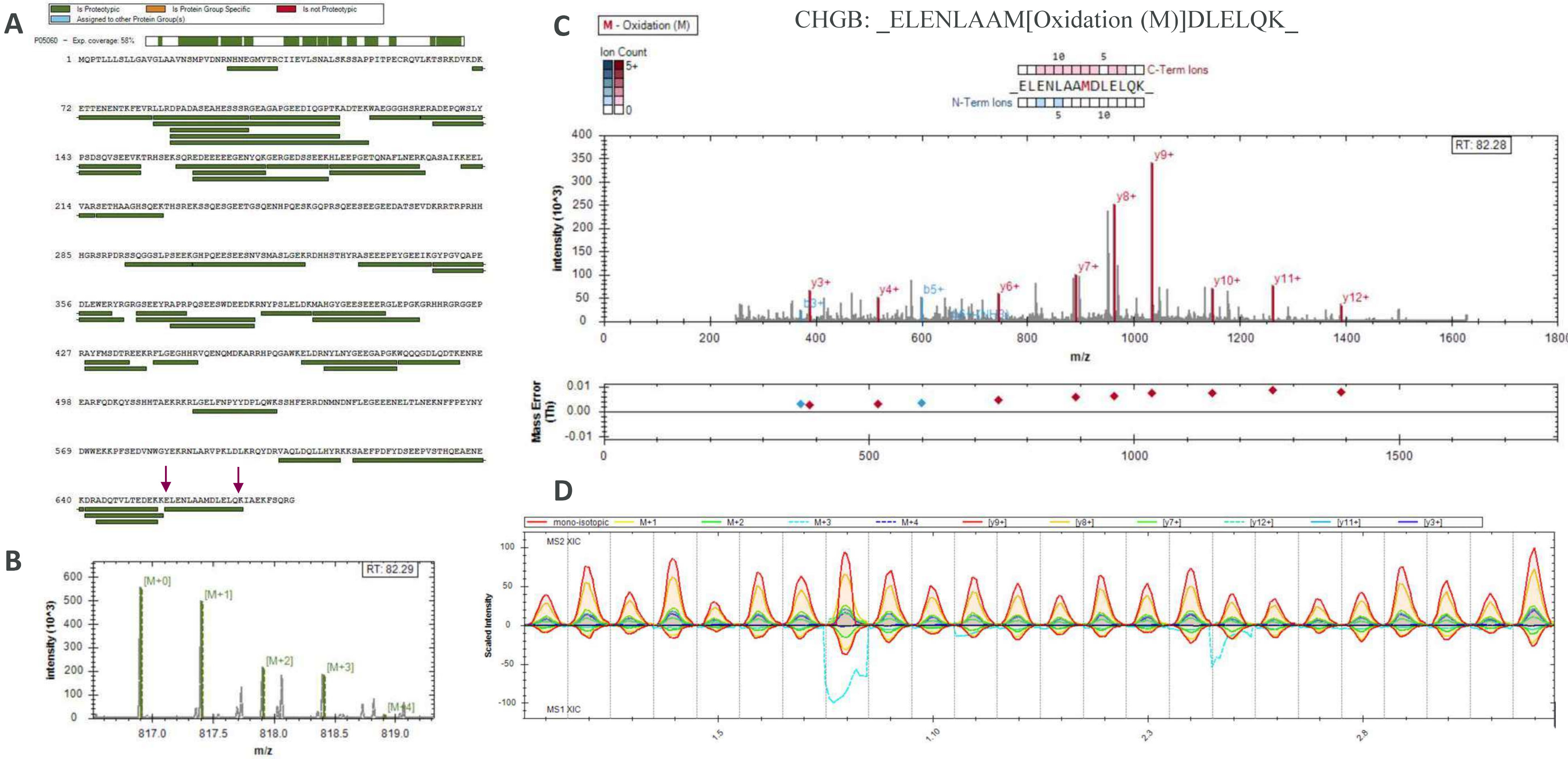

Fig. S32. Chromogranin B proteotypic peptides identified in PXD027913. Total protein coverage (**A**), MS1 isotopic envelope (**B**), MS/MS fragmentation match and mass error (**C**), and XIC of MS1 & MS2 for the conditions (**D**) are shown for each given peptide identified.

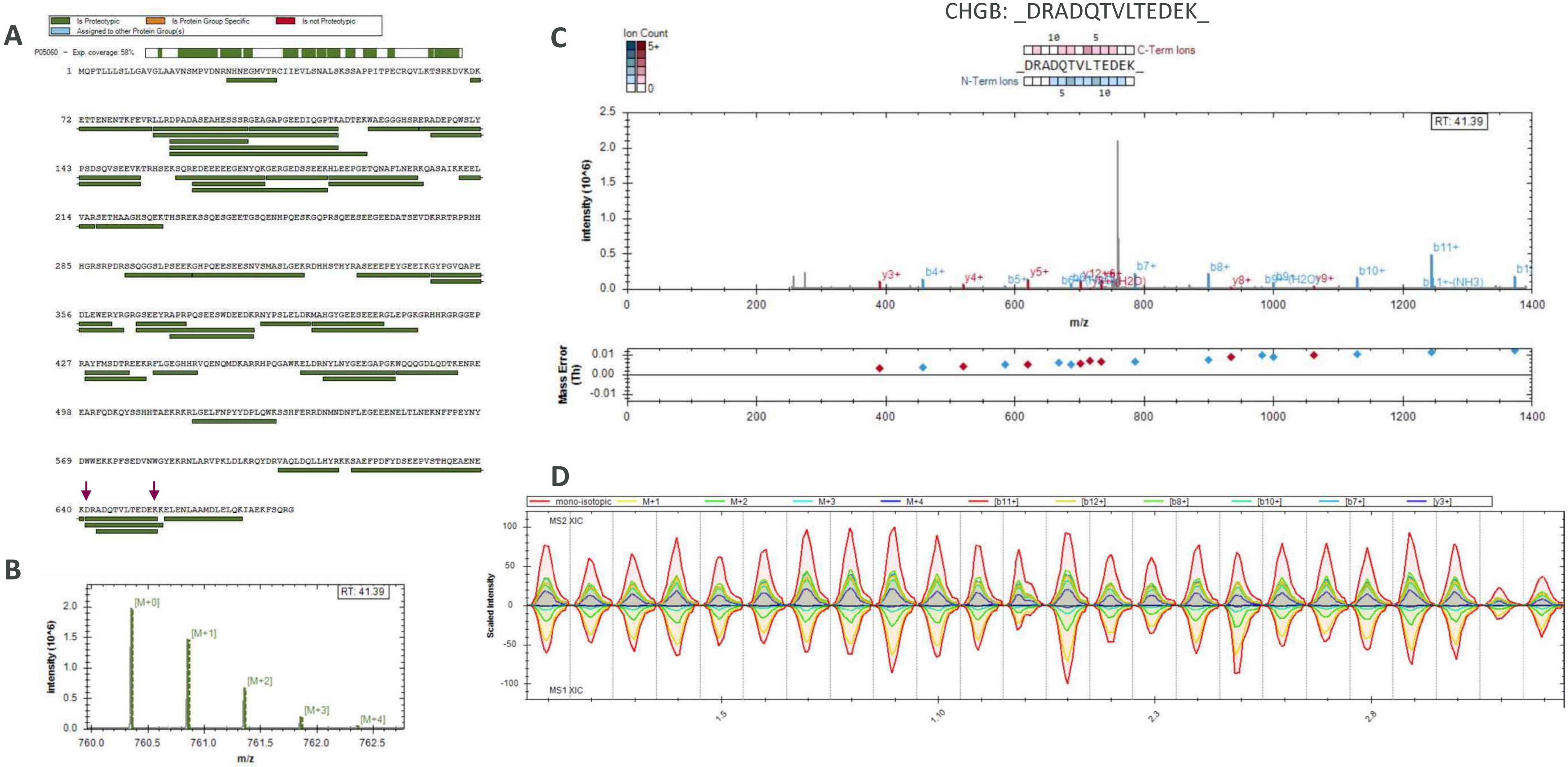

Fig. S33. Chromogranin B proteotypic peptides identified in PXD027913. Total protein coverage (**A**), MS1 isotopic envelope (**B**), MS/MS fragmentation match and mass error (**C**), and XIC of MS1 & MS2 for the conditions (**D**) are shown for each given peptide identified.

Fig S34. Potential VGF bioactive peptides in secretome of EndoC-βH1 cells. CLUSTAL O(1.2.4) multiple sequence alignment of bioactive peptide sequences (yellow) vs peptide sequences detected in the DIA secretome experiment at 72h. Alignment was performed online with UniProt (https://www.uniprot.org/align/).

(A) Neuroendocrine regulatory peptide-1

```
Reference RPESALLGGSEAGERLLQQGLAQVEA
Peptide 1 RPESALLGGSEAGER-----------
Peptide 2 -PESALLGGSEAGER-----------
```

(B) Neuroendocrine regulatory peptide-2

```
Reference QAEATRQAAAQEERLADLASDLLLQYLLQGGARQRGLG
Peptide 1 --------------LADLASDLLLQYLLQGGAR-----
```

(C) TLQP-62

```
Reference TLQPPSALRRRHYHHALPPSRHYPGREAQARRAQEEAEAEERRLQEQEELENYIEHVLLRRP
Peptide 1 ------------------------------------------RLQEQEELENYIEHVLLR--
Peptide 2 -------------------------------RAQEEAEAEER--------------------
Peptide 3 --------------------------------AQEEAEAEER--------------------
Peptide 4 -------------------------------------------LQEQEELENYIEHVLLR--
Peptide 5 --------------------------------AQEEAEAEERR-------------------
Peptide 6 -------------------------------------------LQEQEELENYIEHVLLRR-
Peptide 7 -------------------------------RAQEEAEAEERR-------------------
```

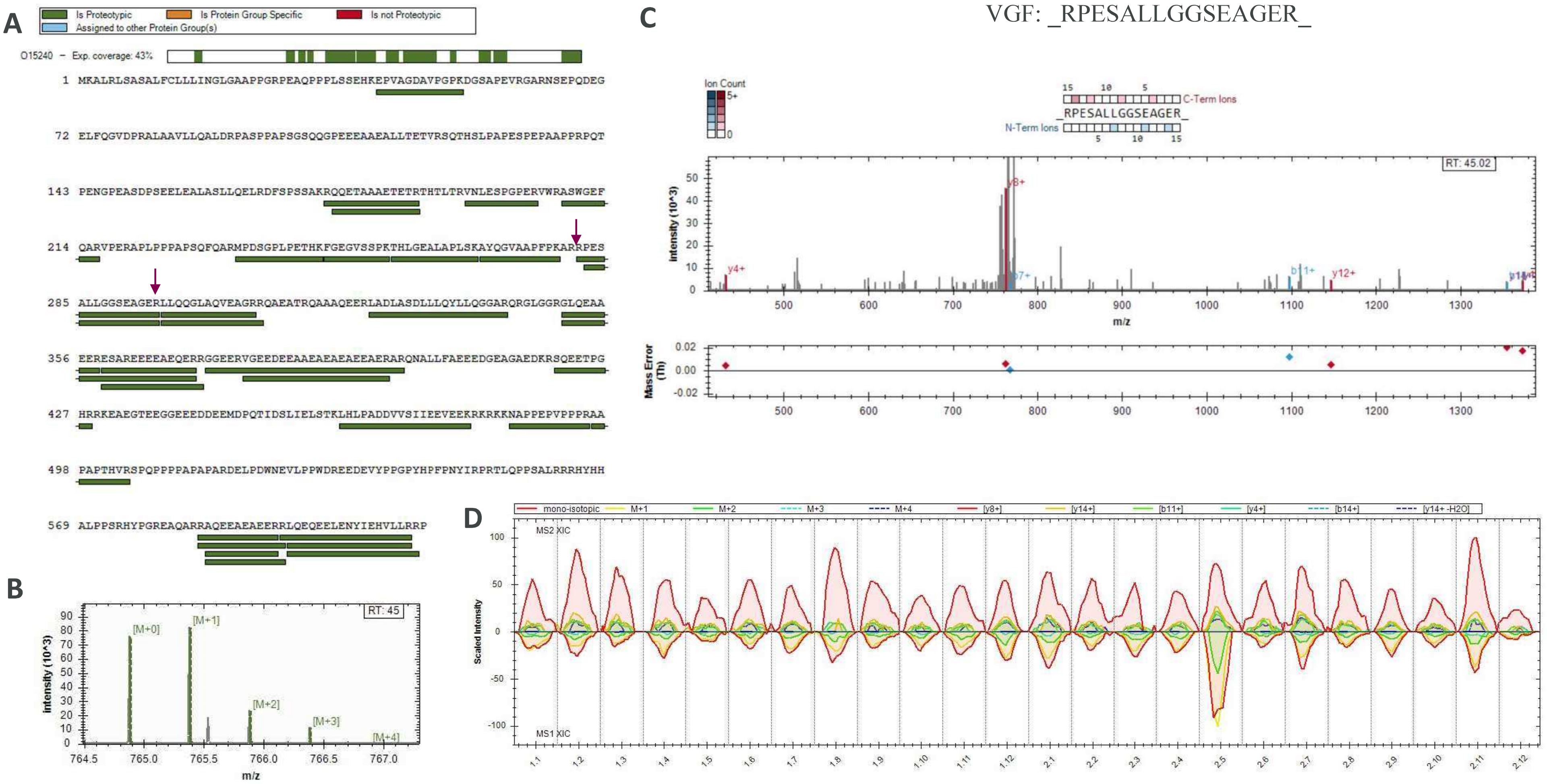

Fig. S35. VGF proteotypic peptides identified in PXD027913. Total protein coverage (**A**), MS1 isotopic envelope (**B**), MS/MS fragmentation match and mass error (**C**), and XIC of MS1 & MS2 for the conditions (**D**) are shown for each given peptide identified.

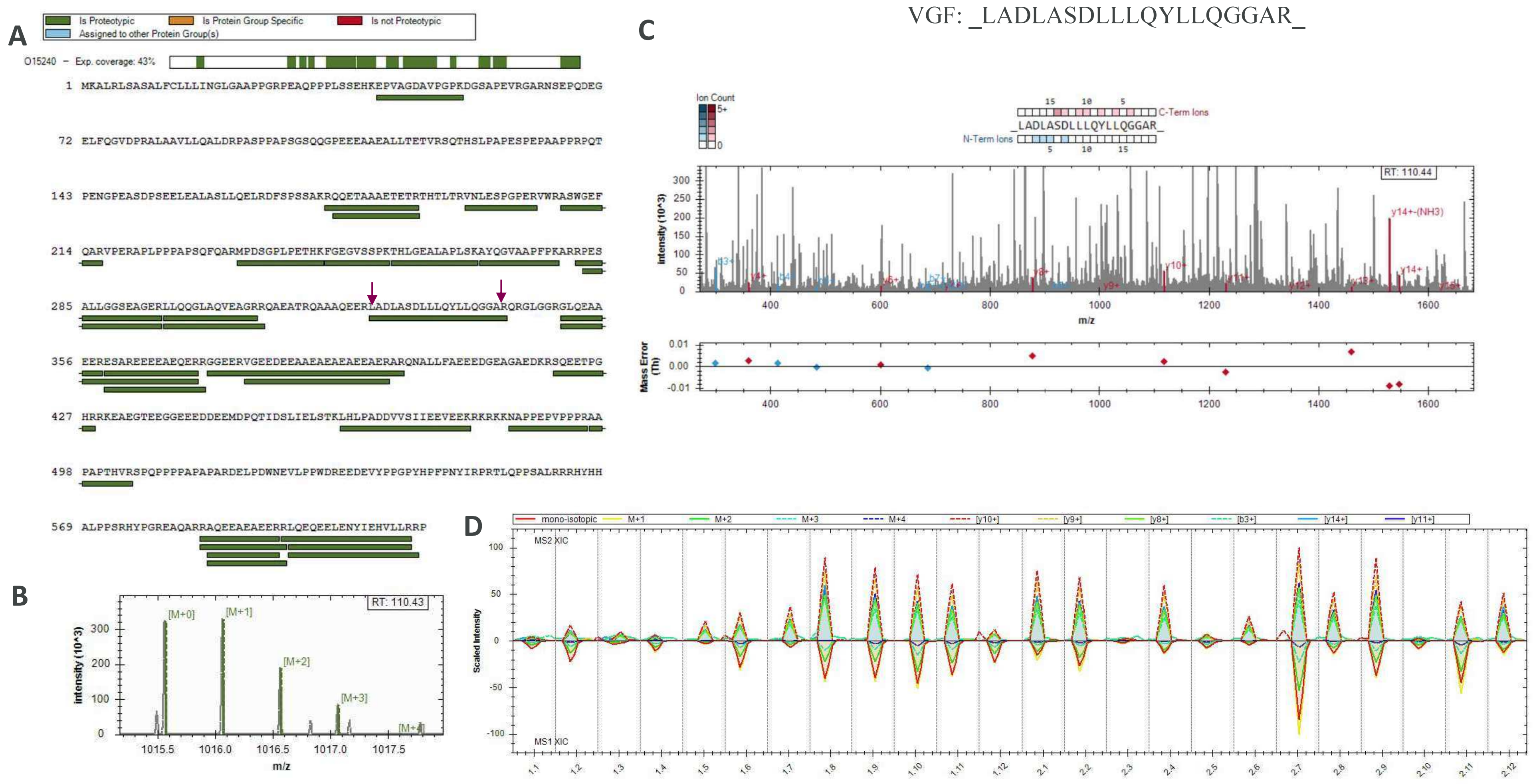

Fig. S36. VGF proteotypic peptides identified in PXD027913. Total protein coverage (**A**), MS1 isotopic envelope (**B**), MS/MS fragmentation match and mass error (**C**), and XIC of MS1 & MS2 for the conditions (**D**) are shown for each given peptide identified.

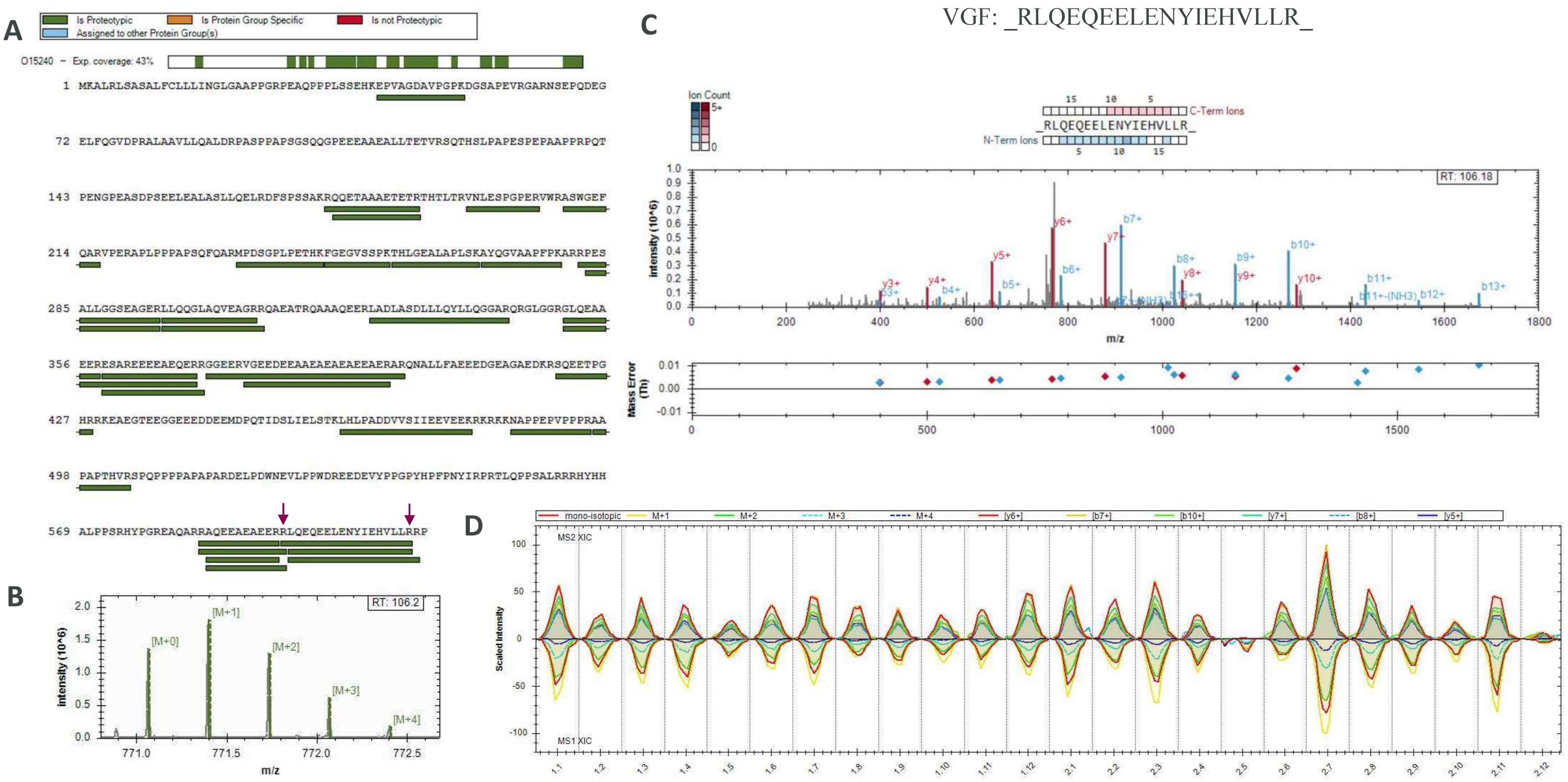

Fig. S37. VGF proteotypic peptides identified in PXD027913. Total protein coverage (**A**), MS1 isotopic envelope (**B**), MS/MS fragmentation match and mass error (**C**), and XIC of MS1 & MS2 for the conditions (**D**) are shown for each given peptide identified.

Fig S38. Potential bioactive peptides from other proteins in secretome of EndoC-βH1 cells. CLUSTAL O(1.2.4) multiple sequence alignment of bioactive peptide sequences (yellow) vs peptide sequences detected in the DIA secretome experiment at 72h. Alignment was performed online with UniProt (https://www.uniprot.org/align/).

(A) C-terminal peptide from Secretogranin-5

```
Reference SVPHFSDEDKDPE
Peptide 1 SVPHFSDEDK---
```

(B) Manserin from Secretogranin-2

```
Reference VPGQGSSEDDLQEEEQIEQAIKEHLNQGSSQETDKLAPVS
Peptide 1 VPGQGSSEDDLQEEEQIEQAIK------------------
Peptide 2 VPGQGSSEDDLQEEEQIEQAIKEHLNQGSSQETDK-----
Peptide 3 ----------------------EHLNQGSSQETDK-----
```

(C) NEUG(55-78) from Neurogranin

```
Reference GPGPGGPGGAGVARGGAGGGPSGD
Peptide 1 GPGPGGPGGAGVAR----------
```

(D) Thymosin alpha 1 from Prothymosin alpha

```
Reference SDAAVDTSSEITTKDLKEKKEVVEEAEN
Peptide 1 SDAAVDTSSEITTKDLK-----------
```

(E) Thymopoietin from TMPO

```
Reference PEFLEDPSVLTKDKLKSELVANNVTLPAGEQRKDVYVQLYLQHLTARNR
Peptide 1 ---------------------------------DVYVQLYLQHLTAR–
Peptide 2 ----------------SELVANNVTLPAGEQRK----------------
Peptide 3 ----------------SELVANNVTLPAGEQR-----------------
Peptide 4 PEFLEDPSVLTK-------------------------------------
```

(F) CNP-53 from C-type natriuretic peptide

```
Reference DLRVDTKSRAAWARLLQEHPNARKYKGANKKGLSKGCFGLKLDRIGSMSGLGC
Peptide 1 --------------LLQEHPNAR------------------------------
```

(G) Preptin from Insulin-like growth factor 2

```
Reference DVSTPPTVLPDNFPRYPVGKFFQYDTWKQSTQRL
Peptide 1 --------------------FFQYDTWK------
```

(H) Short peptide from Alpha-1-antitrypsin

```
Reference MFLEAIPMSIPPEVKFNKPFVFLMIEQNTKSPLFMGKVVNPTQK
Peptide 1 ------------------------------SPLFMGK-------
Peptide 2 ------------------PFVFLMIEQNTK--------------
```

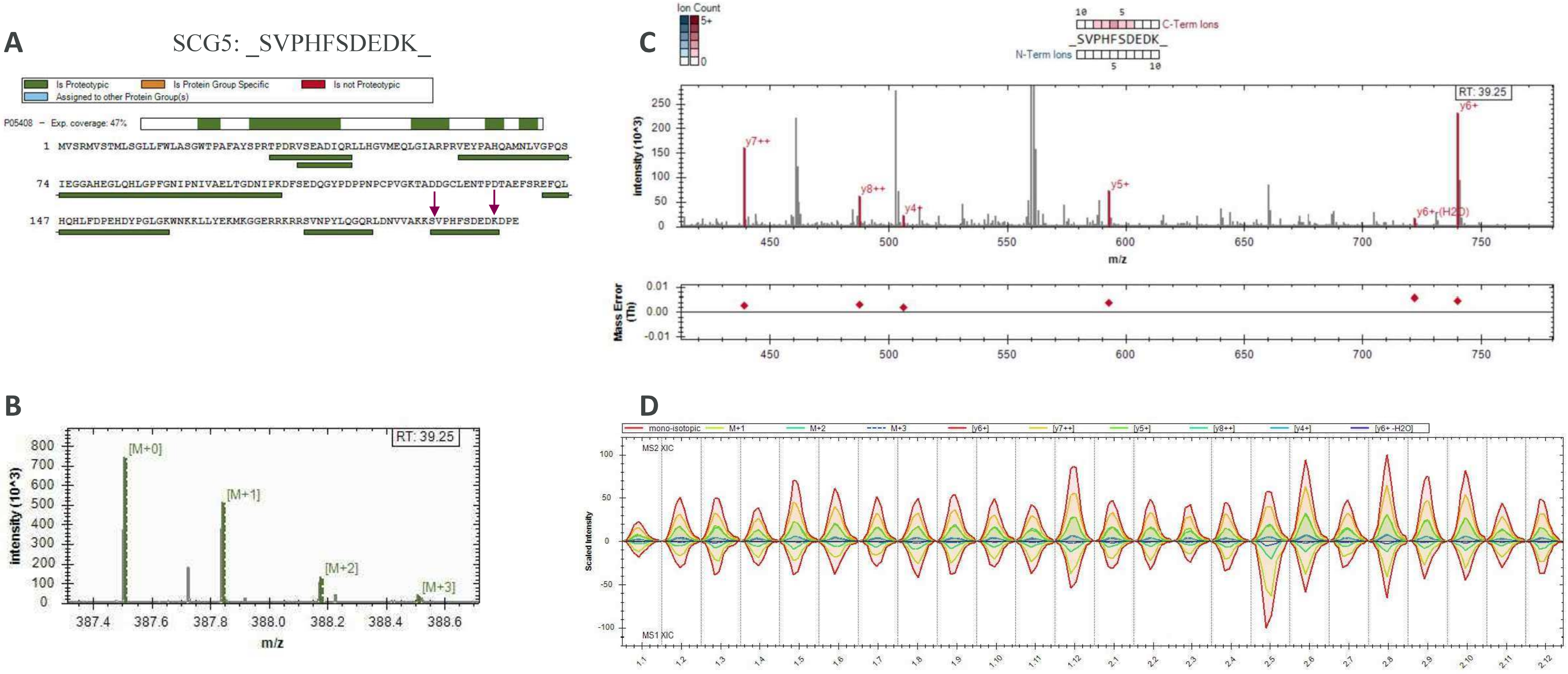

Fig. S39. Proteotypic peptide corresponding to C-terminal peptide of SCG5 identified in PXD027913. Total protein coverage (**A**), MS1 isotopic envelope (**B**), MS/MS fragmentation match and mass error (**C**), and XIC of MS1 & MS2 for the conditions (**D**) are shown for each given peptide identified.

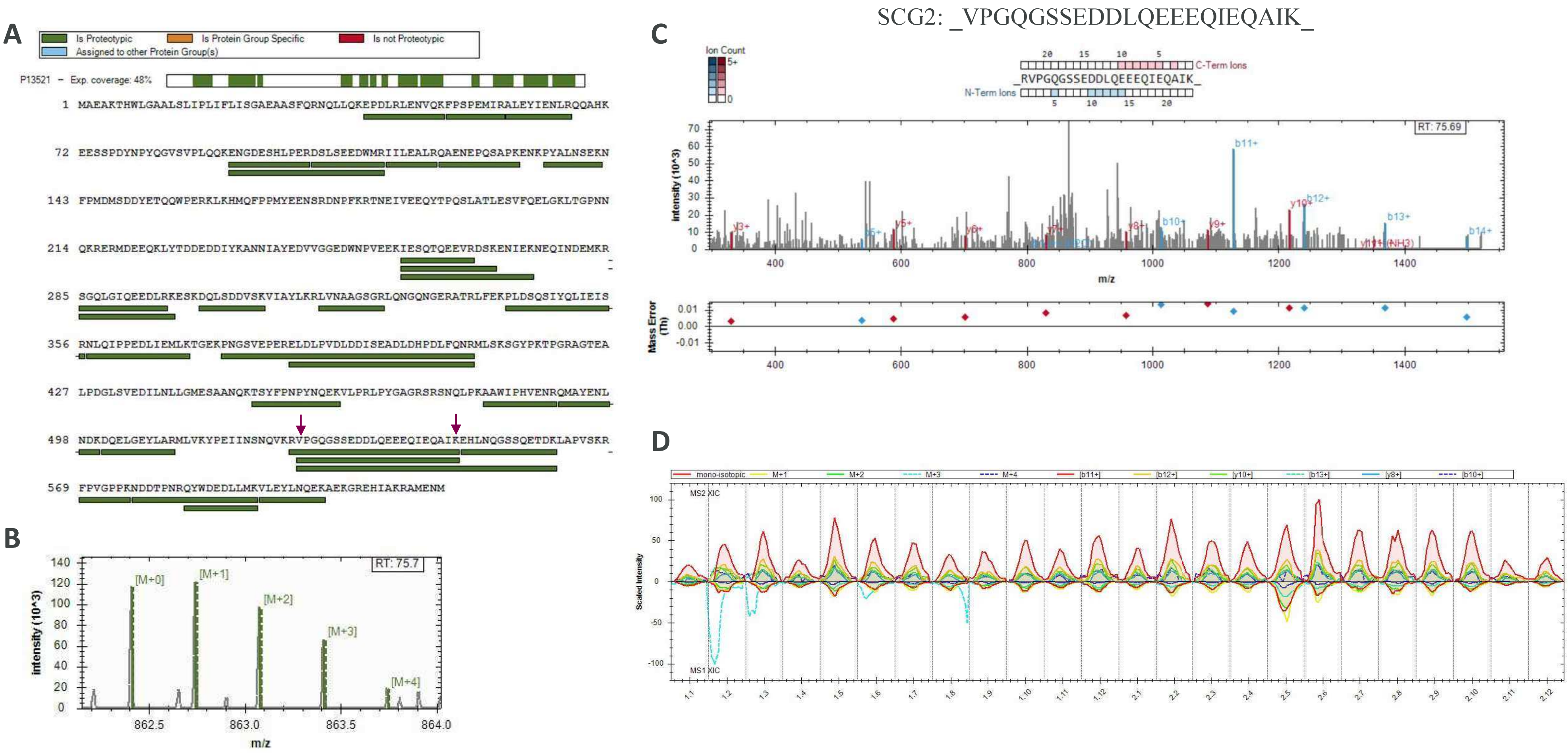

Fig. S40. Proteotypic peptides corresponding to Manserin of SCG2 identified in PXD027913. Total protein coverage (**A**), MS1 isotopic envelope (**B**), MS/MS fragmentation match and mass error (**C**), and XIC of MS1 & MS2 for the conditions (**D**) are shown for each given peptide identified.

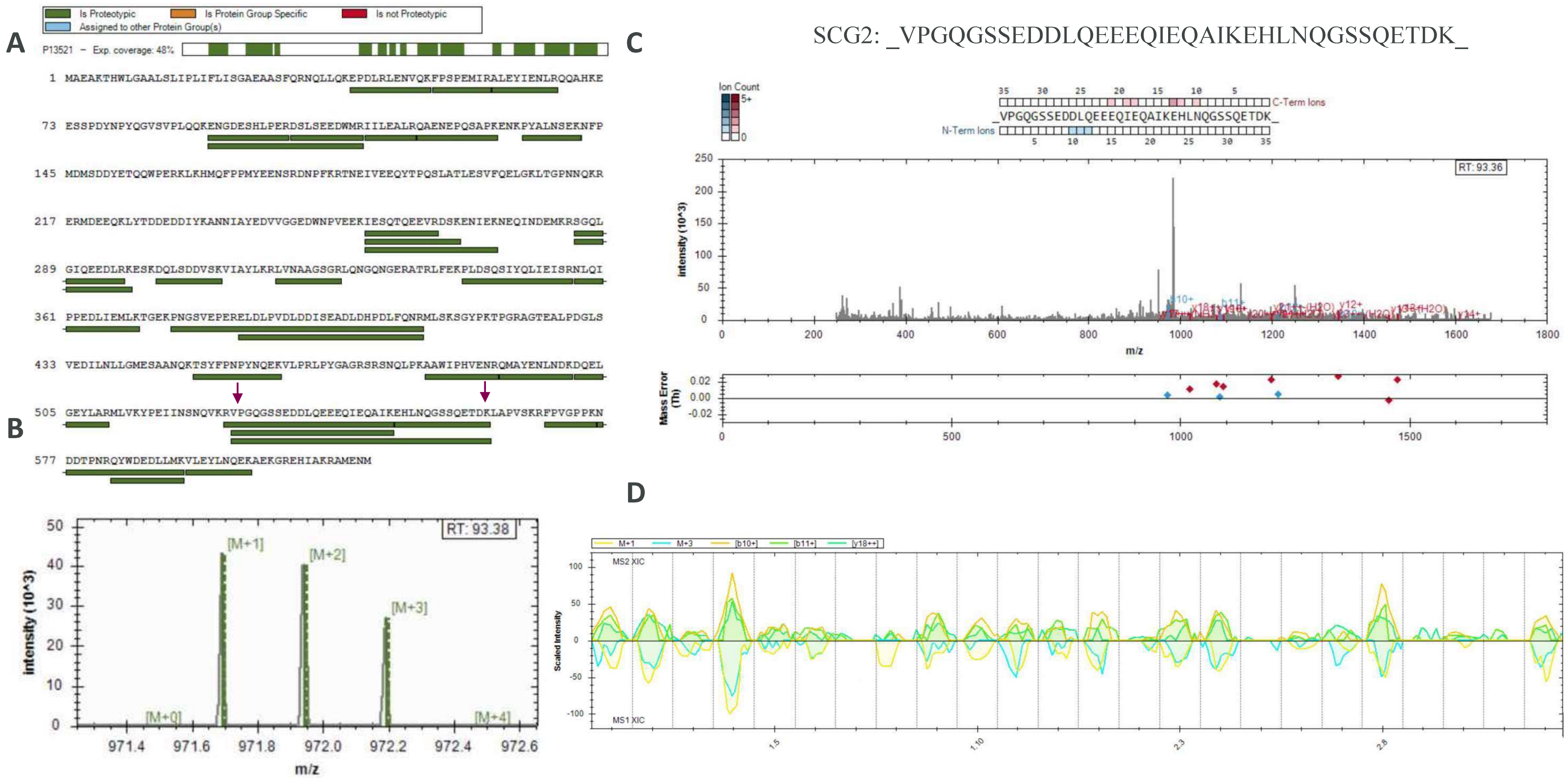

Fig. S41. Proteotypic peptides corresponding to Manserin of SCG2 identified in PXD027913. Total protein coverage (**A**), MS1 isotopic envelope (**B**), MS/MS fragmentation match and mass error (**C**), and XIC of MS1 & MS2 for the conditions (**D**) are shown for each given peptide identified.

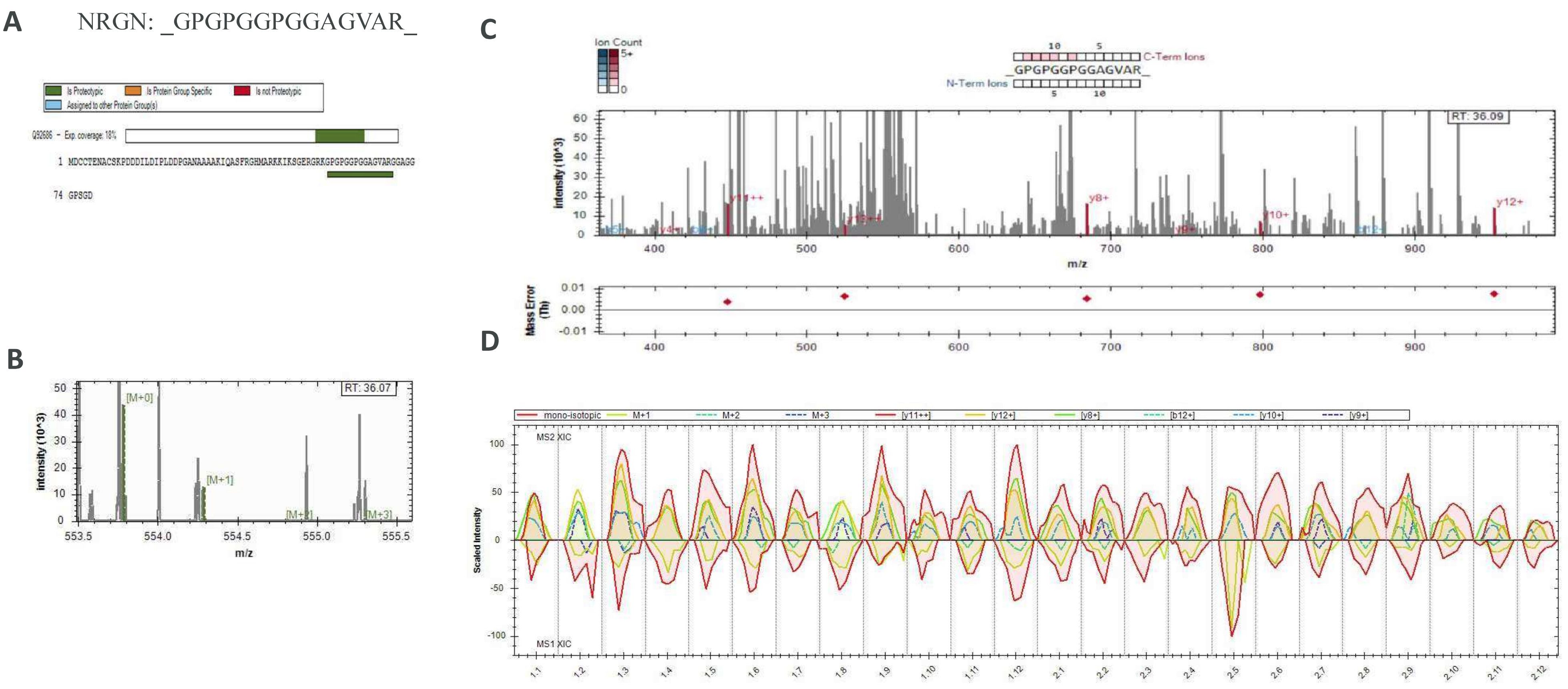

Fig. S42. Proteotypic peptide corresponding to NEUG(55-78) from Neurogranin identified in PXD027913. Total protein coverage (**A**), MS1 isotopic envelope (**B**), MS/MS fragmentation match and mass error (**C**), and XIC of MS1 & MS2 for the conditions (**D**) are shown for each given peptide identified.

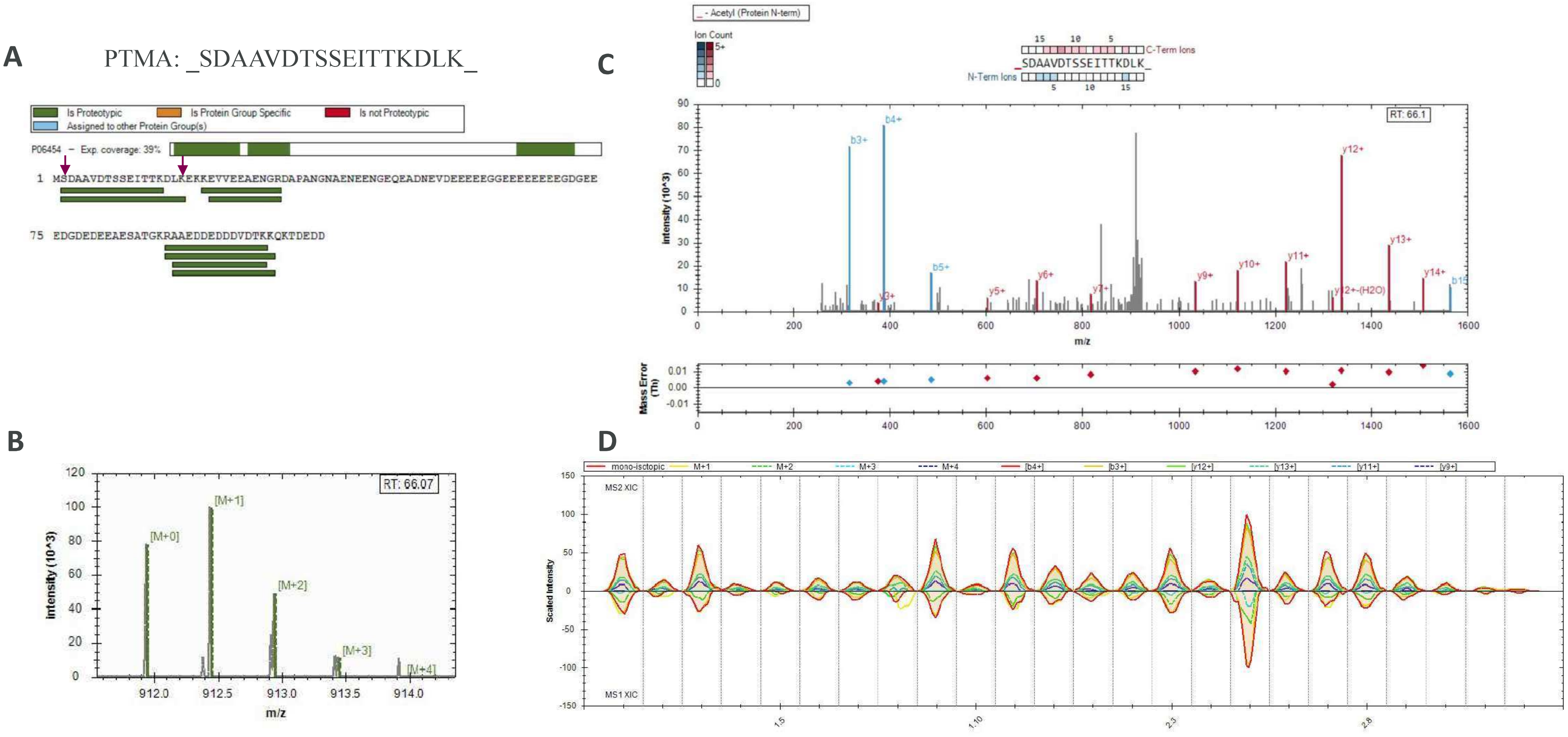

Fig. S43. Proteotypic peptide corresponding to Thymosin alpha 1 identified in PXD027913. Total protein coverage (**A**), MS1 isotopic envelope (**B**), MS/MS fragmentation match and mass error (**C**), and XIC of MS1 & MS2 for the conditions (**D**) are shown for each given peptide identified.

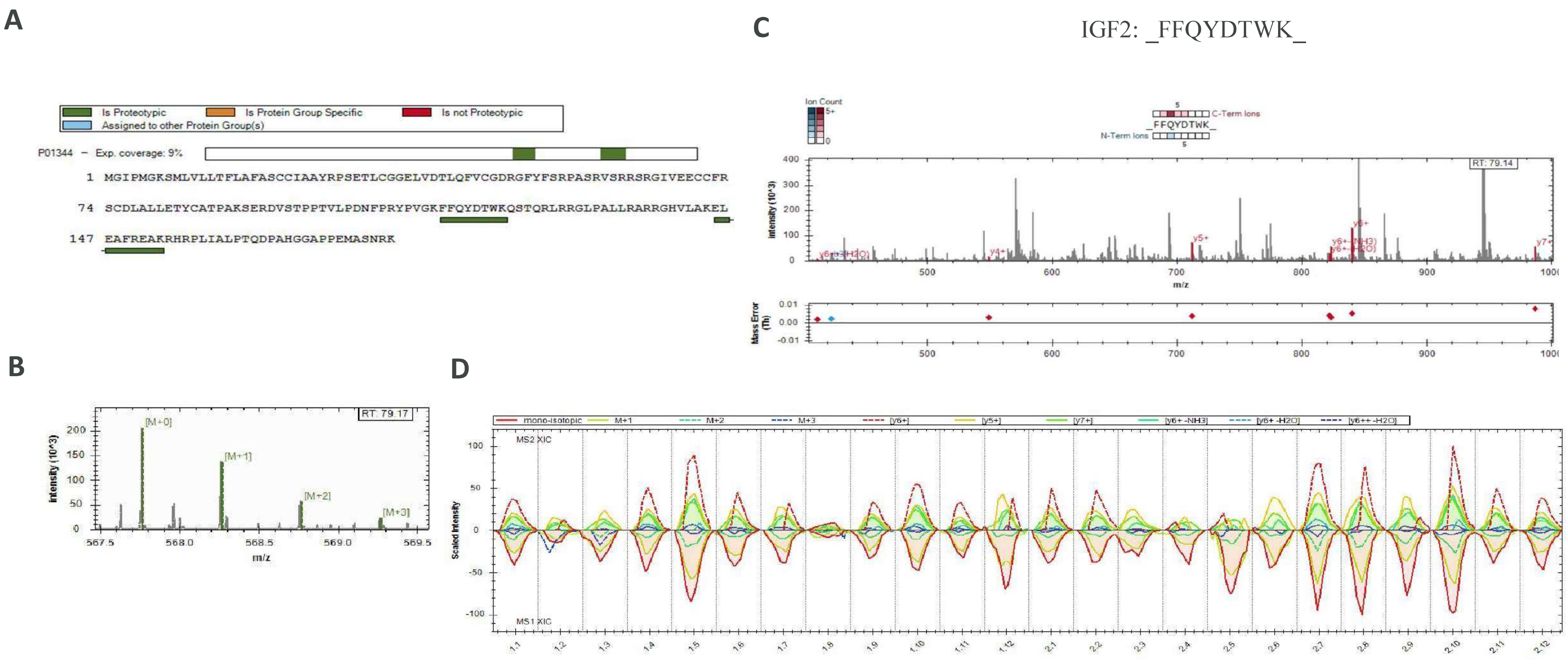

Fig. S44. Proteotypic peptide corresponding to preptin in DIA secretome proteomics experiment at 72h. Total protein coverage (**A**), MS1 isotopic envelope (**B**), MS/MS fragmentation match and mass error (**C**), and XIC of MS1 & MS2 for the conditions (**D**) are shown for each given peptide identified.

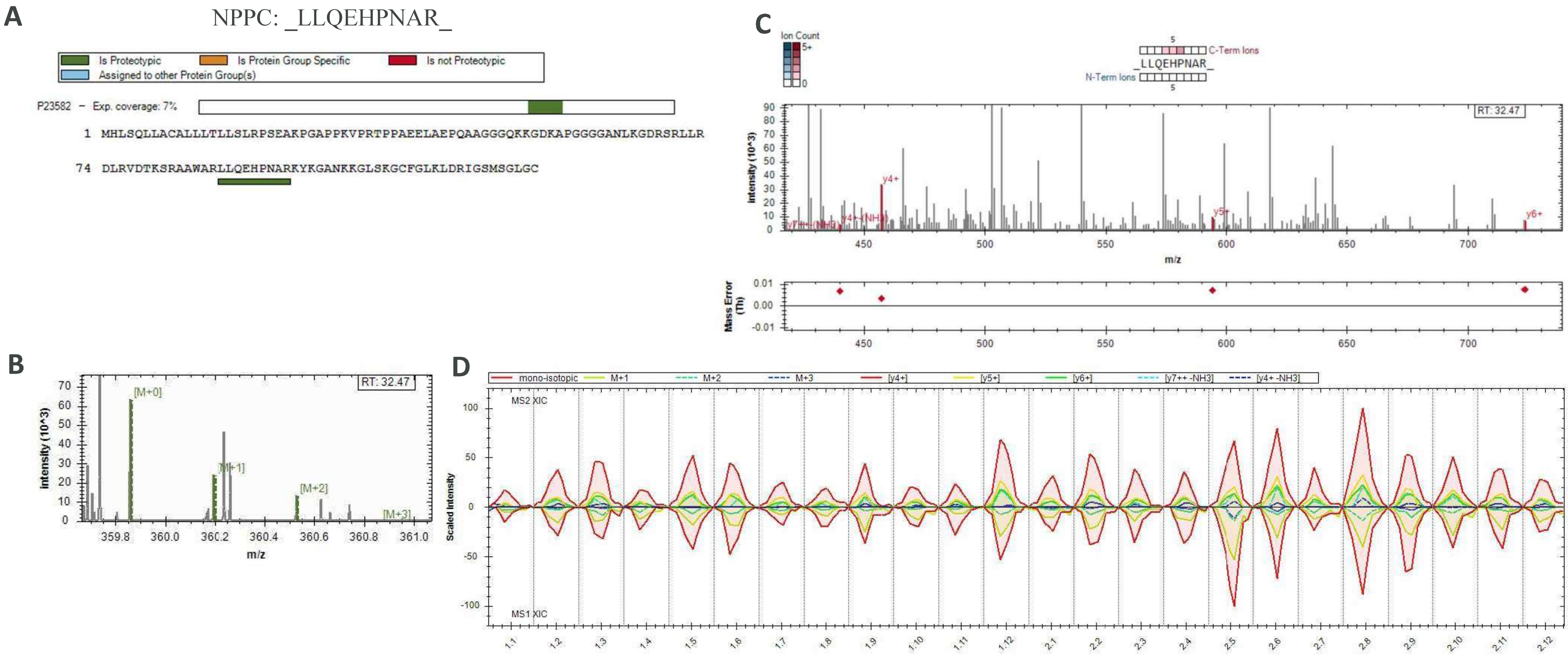

Fig. S45. Proteotypic peptide corresponding to CNP-53 from C-type natriuretic peptide in DIA secretome proteomics experiment at 72h. Total protein coverage (**A**), MS1 isotopic envelope (**B**), MS/MS fragmentation match and mass error (**C**), and XIC of MS1 & MS2 for the conditions (**D**) are shown for each given peptide identified.

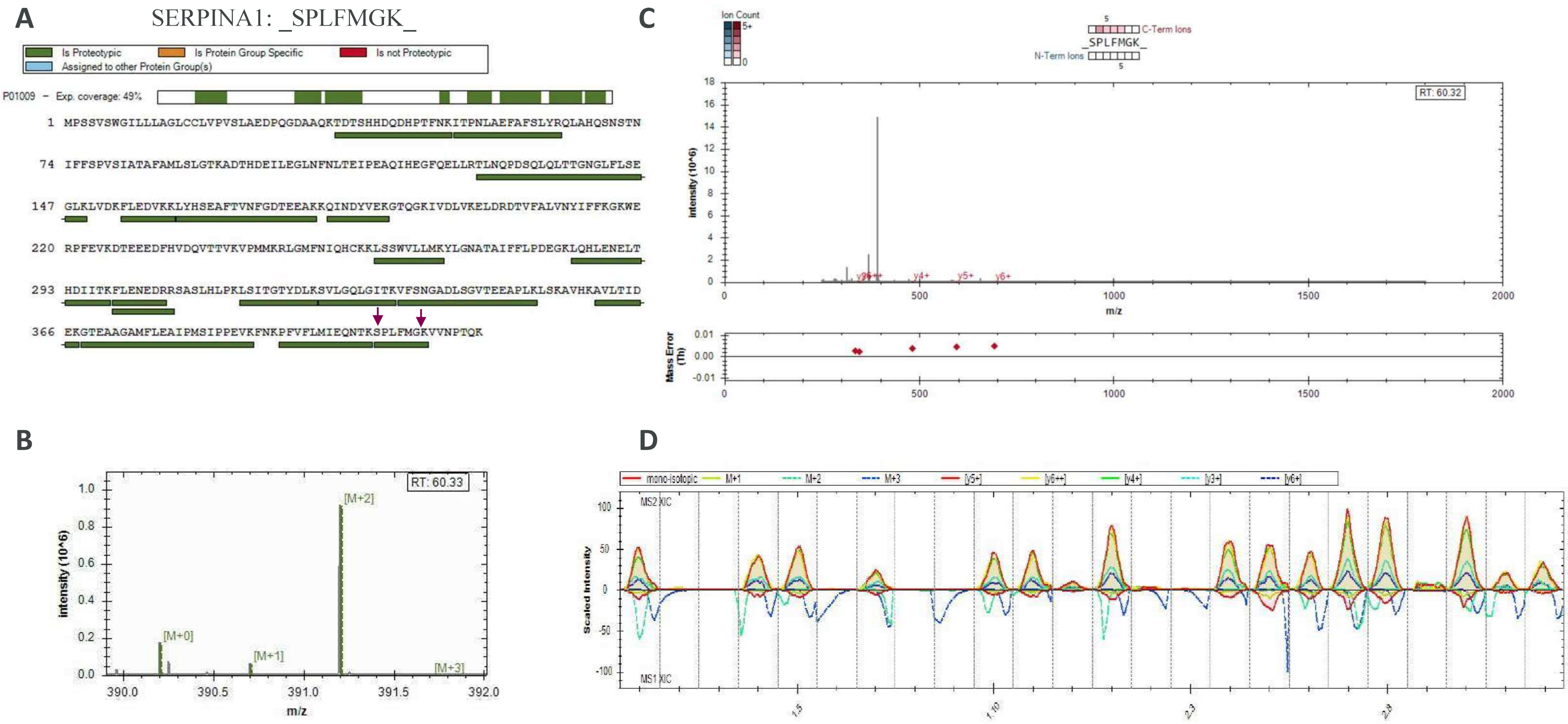

Fig. S46. Proteotypic peptides corresponding to SERPINA1 short peptide in DIA secretome proteomics experiment at 72h. Total protein coverage (**A**), MS1 isotopic envelope (**B**), MS/MS fragmentation match and mass error (**C**), and XIC of MS1 & MS2 for the conditions (**D**) are shown for each given peptide identified.

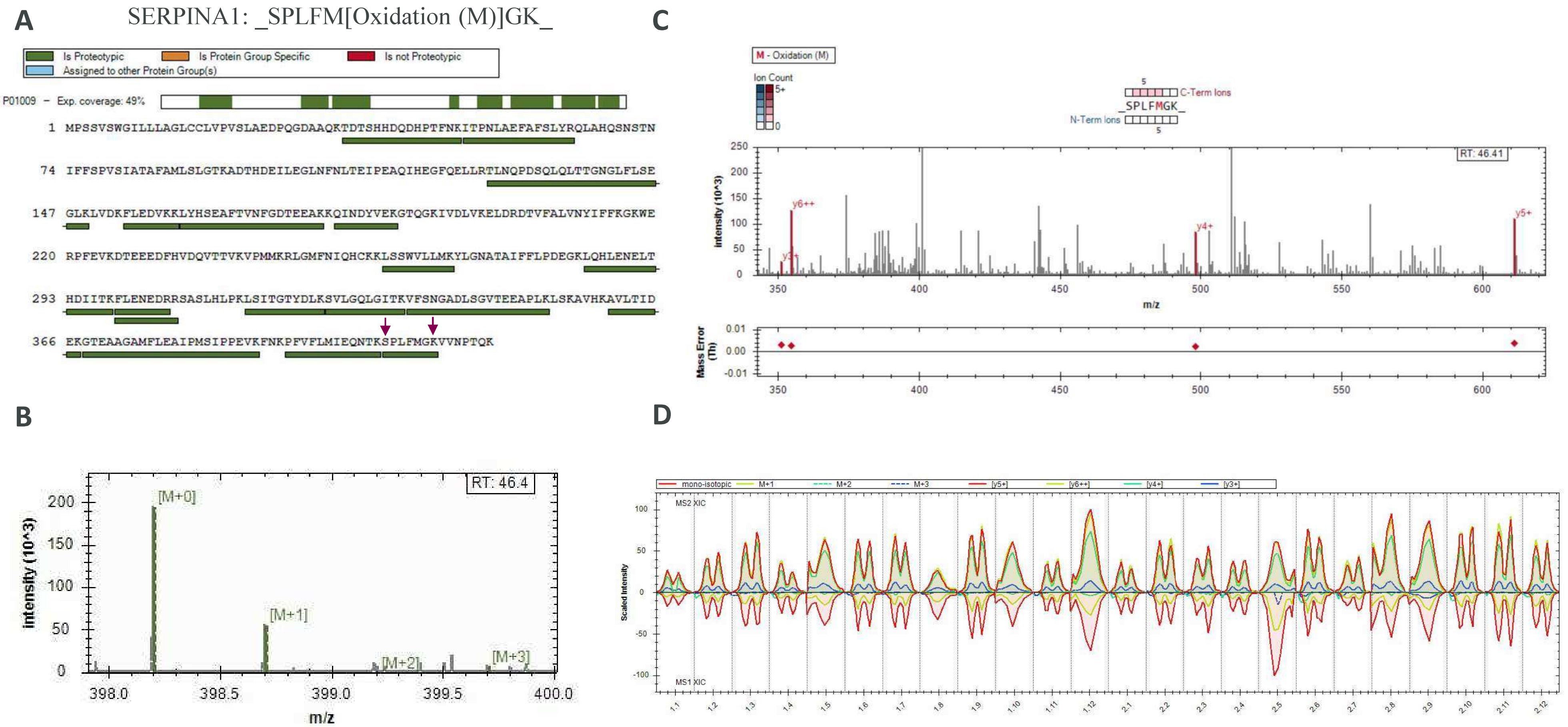

Fig. S47. Proteotypic peptides corresponding to SERPINA1 short peptide in DIA secretome proteomics experiment at 72h. Total protein coverage (**A**), MS1 isotopic envelope (**B**), MS/MS fragmentation match and mass error (**C**), and XIC of MS1 & MS2 for the conditions (**D**) are shown for each given peptide identified.

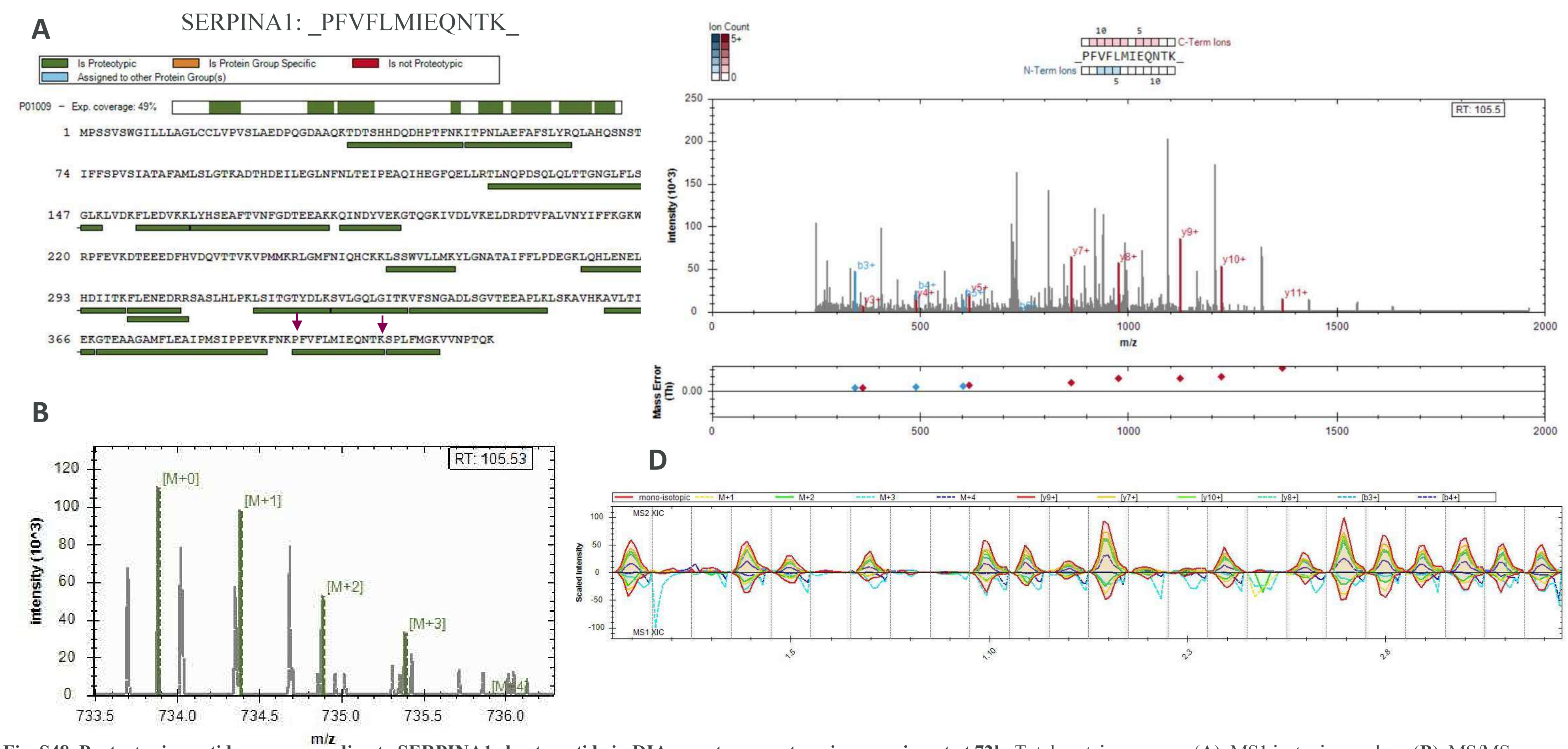

Fig. S48. Proteotypic peptides corresponding to SERPINA1 short peptide in DIA secretome proteomics experiment at 72h. Total protein coverage (**A**), MS1 isotopic envelope (**B**), MS/MS fragmentation match and mass error (**C**), and XIC of MS1 & MS2 for the conditions (**D**) are shown for each given peptide identified.

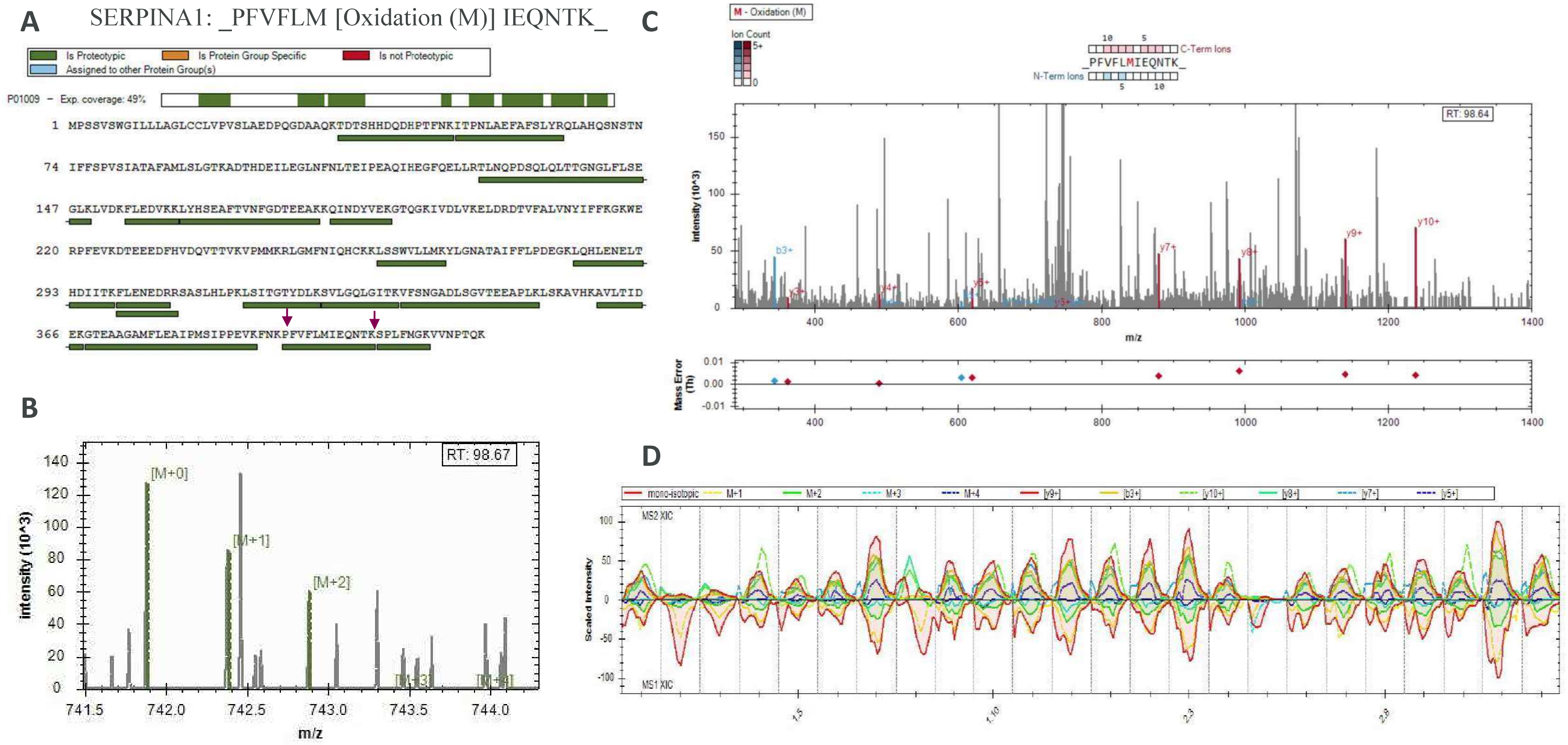

Fig. S49. Proteotypic peptides corresponding to SERPINA1 short peptide in DIA secretome proteomics experiment at 72h. Total protein coverage (**A**), MS1 isotopic envelope (**B**), MS/MS fragmentation match and mass error (**C**), and XIC of MS1 & MS2 for the conditions (**D**) are shown for each given peptide identified.

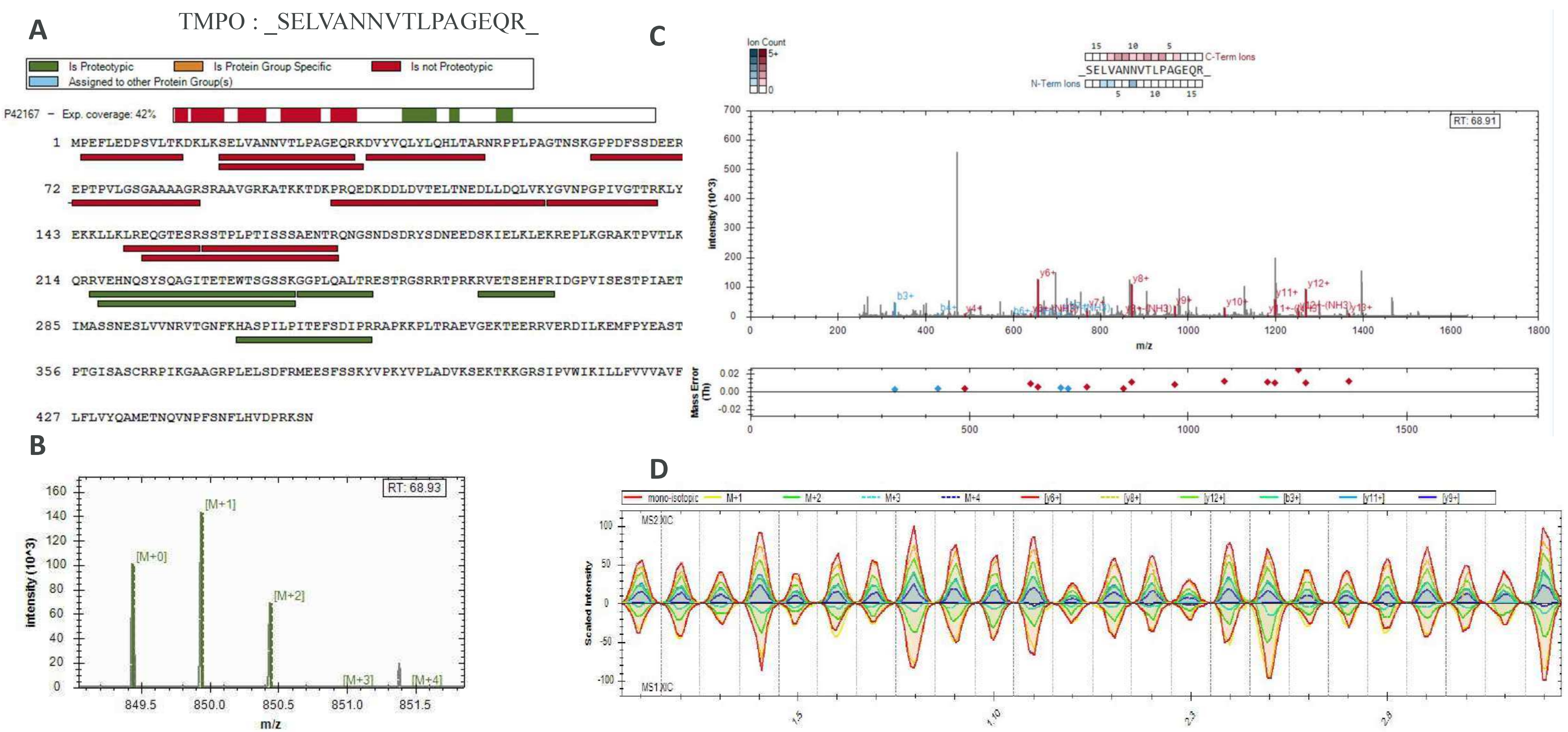

Fig. S50. Proteotypic peptides corresponding to Thymopoietin in DIA secretome proteomics experiment at 72h. Total protein coverage (**A**), MS1 isotopic envelope (**B**), MS/MS fragmentation match and mass error (**C**), and XIC of MS1 & MS2 for the conditions (**D**) are shown for each given peptide identified.

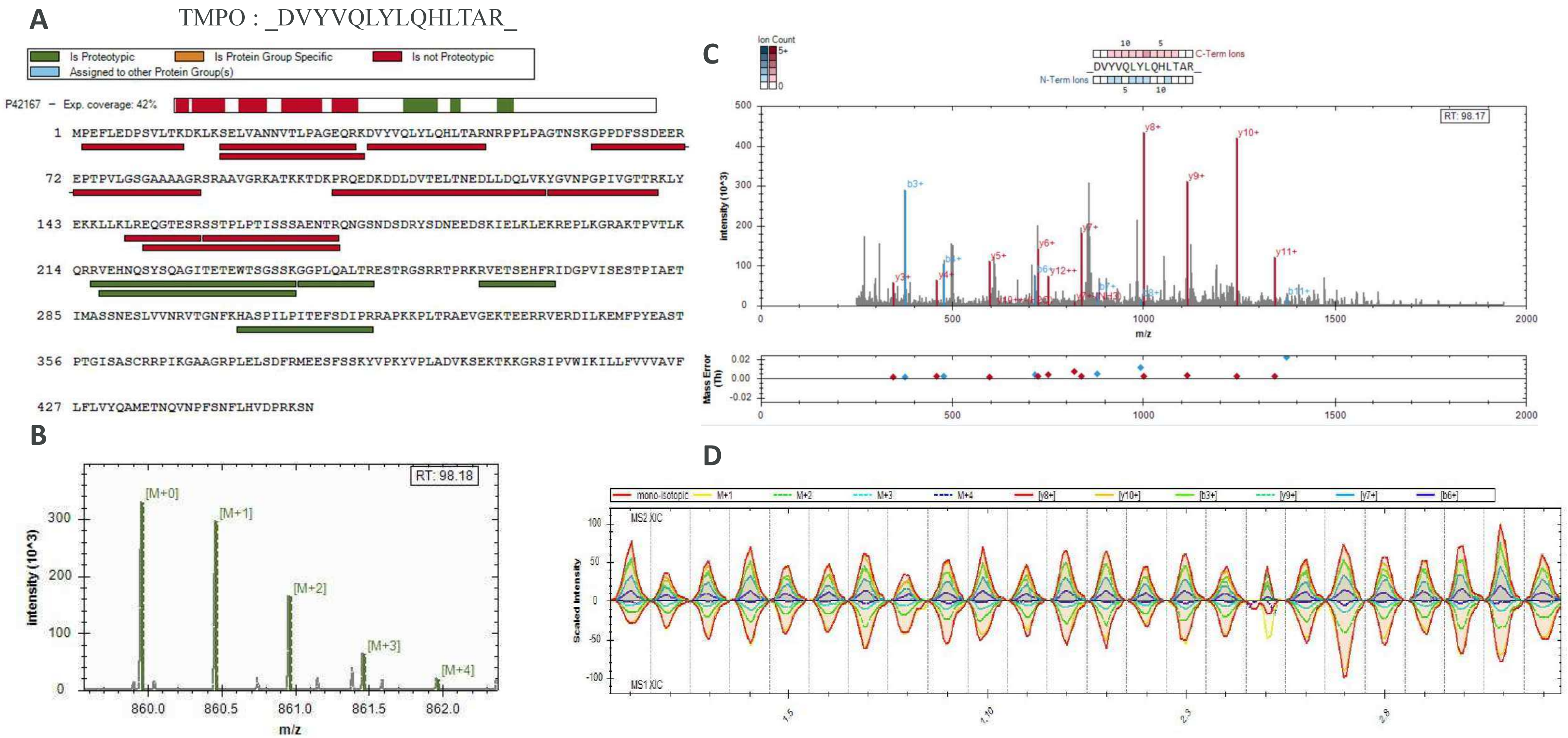

Fig. S51. Proteotypic peptides corresponding to Thymopoietin in DIA secretome proteomics experiment at 72h. Total protein coverage (**A**), MS1 isotopic envelope (**B**), MS/MS fragmentation match and mass error (**C**), and XIC of MS1 & MS2 for the conditions (**D**) are shown for each given peptide identified.

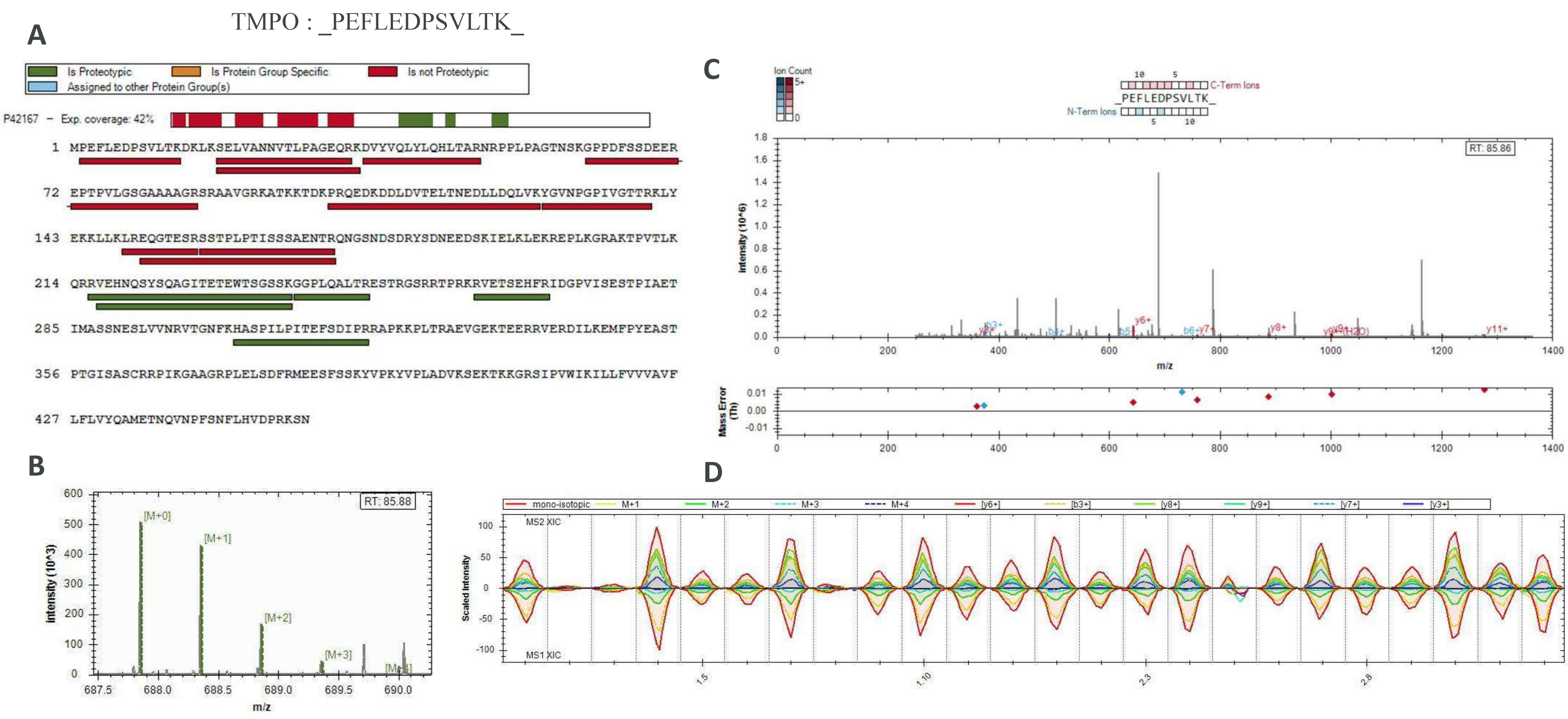

Fig. S52. Proteotypic peptides corresponding to Thymopoietin in DIA secretome proteomics experiment at 72h. Total protein coverage (**A**), MS1 isotopic envelope (**B**), MS/MS fragmentation match and mass error (**C**), and XIC of MS1 & MS2 for the conditions (**D**) are shown for each given peptide identified.

Table S4. Top 150 secreted proteins. Proteins are ordered by decreasing median normalized expression abundance in untransfected EndoC-βH1 cells at day 0 in PXD027920. N=3.

rank	UniProt	Symbol	rank	UniProt	Symbol	rank	UniProt	Symbol
1	Q8N1F7	NUP93	51	P23528	CFL1	101	P09936	UCHL1
2	P10645	CHGA	52	Q02790	FKBP4	102	P12814	ACTN1
3	P04264	KRT1	53	P53396	ACLY	103	P67936	TPM4
4	P60709	ACTB	54	P40925	MDH1	104	O43707	ACTN4
5	P02790	HPX	55	P02751	FN1	105	Q8NCW5	NAXE
6	P05787	KRT8	56	P01859	IGHG2	106	P26038	MSN
7	P02774	GC	57	Q92797	SYMPK	107	Q86YZ3	HRNR
8	P49257	LMAN1	58	P14625	HSP90B1	108	P00387	CYB5R3
9	P04259	KRT6B	59	P07737	PFN1	109	Q92820	GGH
10	P01857	IGHG1	60	P20700	LMNB1	110	P55327	TPD52
11	P01861	IGHG4	61	P08727	KRT19	111	P47755	CAPZA2
12	O14497	ARID1A	62	P30040	ERP29	112	P15531	NME1
13	P02533	KRT14	63	P61970	NUTF2	113	P00492	HPRT1
14	P01834	IGKC	64	P04406	GAPDH	114	P61916	NPC2
15	P60174	TPI1	65	P13010	XRCC5	115	Q8IV08	PLD3
16	P08670	VIM	66	P10599	TXN	116	Q99497	PARK7
17	Q15078	CDK5R1	67	Q15365	PCBP1	117	P26447	S100A4
18	P62937	PPIA	68	P27797	CALR	118	P07942	LAMB1
19	P35527	KRT9	69	P04080	CSTB	119	Q9UJ72	ANXA10
20	P62805	H4C1	70	P05783	KRT18	120	P25398	RPS12
21	P13645	KRT10	71	P01040	CSTA	121	Q9HC38	GLOD4
22	P55884	EIF3B	72	P35030	PRSS3	122	Q15293	RCN1
23	P35908	KRT2	73	P61769	B2M	123	P14618	PKM
24	Q14624	ITIH4	74	P29401	TKT	124	O00469	PLOD2
25	P04792	HSPB1	75	Q06830	PRDX1	125	P07954	FH
26	P00441	SOD1	76	P11142	HSPA8	126	P62826	RAN
27	P20930	FLG	77	P18669	PGAM1	127	P50991	CCT4
28	P62736	ACTA2	78	P06733	ENO1	128	O94760	DDAH1
29	P81605	DCD	79	P07237	P4HB	129	P13929	ENO3
30	P02766	TTR	80	P12273	PIP	130	Q15363	TMED2
31	P62258	YWHAE	81	P30048	PRDX3	131	P09104	ENO2
32	O00159	MYO1C	82	P00352	ALDH1A1	132	Q16775	HAGH
33	P16870	CPE	83	Q969H8	MYDGF	133	P25705	ATP5F1A
34	P23284	PPIB	84	P61956	SUMO2	134	Q02383	SEMG2
35	P04279	SEMG1	85	P22626	HNRNPA2B1	135	P06312	IGKV4-1
36	Q03181	PPARD	86	P04075	ALDOA	136	P48637	GSS
37	P14174	MIF	87	P07108	DBI	137	P50395	GDI2
38	P63104	YWHAZ	88	P08195	SLC3A2	138	Q71U36	TUBA1A
39	P31946	YWHAB	89	P09651	HNRNPA1	139	P61457	PCBD1
40	P26885	FKBP2	90	Q9NR12	PDLIM7	140	P11021	HSPA5
41	P40926	MDH2	91	P01308	INS	141	Q15286	RAB35
42	P01876	IGHA1	92	P09622	DLD	142	P05387	RPLP2
43	P13521	SCG2	93	Q04760	GLO1	143	P68371	TUBB4B
44	P61604	HSPE1	94	P06756	ITGAV	144	Q13404	UBE2V1
45	P13647	KRT5	95	Q9BRA2	TXNDC17	145	P09417	QDPR
46	Q16836	HADH	96	Q6VVX0	CYP2R1	146	P09382	LGALS1
47	P13674	P4HA1	97	P01860	IGHG3	147	Q96AY3	FKBP10
48	P24593	IGFBP5	98	P30046	DDT	148	P06744	GPI
49	P43307	SSR1	99	P62277	RPS13	149	Q9Y2B0	CNPY2
50	P30101	PDIA3	100	P07195	LDHB	150	P10412	H1-4
